# Rossby wave-modulated orbital precipitation anomalies in the Asia-Pacific region

**DOI:** 10.1038/s41467-026-74368-3

**Published:** 2026-06-16

**Authors:** Zhaojie Yu, Lina Song, Zhimin Jian, Mahyar Mohtadi, Yair Rosenthal, Kyung-Sook Yun, Xiaojie Tang, Lei Zhang, Yuanlong Li, Mingmei Xie, Peng Hu, Zehua Song, Franck Bassinot, Christophe Colin, Shiming Wan

**Affiliations:** 1 Institute of Oceanology, Chinese Academy of Sciences, Qingdao, China; 2 Laboratory for Marine Geology, Qingdao Marine Science and Technology Center, Qingdao, China; 3 Laboratory for Ocean Dynamics and Climate, Qingdao Marine Science and Technology Center, Qingdao, China; 4 State Key Laboratory of Marine Geology, Tongji University, Shanghai, China; 5 MARUM-Center for Marine Environmental Sciences, University of Bremen, Bremen, Germany; 6 Department of Earth and Planetary Sciences, Rutgers University, New Brunswick, NJ USA; 7 Center for Climate Physics, Institute for Basic Science, Busan, South Korea; 8 Pusan National University, Busan, South Korea; 9 Université Paris-Saclay, CNRS, GEOPS, Orsay, France; 10 State Key Laboratory of Tropical Oceanography, South China Sea Institute of Oceanology, Chinese Academy of Sciences, Guangzhou, China; 11 Frontier Science Center for Deep Ocean Multispheres and Earth System, Physical Oceanography Laboratory, Ocean University of China, Qingdao, China; 12 Department of Atmospheric Sciences, Yunnan University, Kunming, China; 13 LSCE/IPSL, CEA-CNRS-UVSQ, Université Paris-Saclay, Gif-sur-Yvette, France

**Keywords:** Palaeoclimate, Palaeoceanography

## Abstract

Orbital-scale dynamics of Indo-Pacific Warm Pool (IPWP), Earth’s dominant heat and moisture source, exert far-reaching yet incompletely understood influences on global hydroclimate. Here, by synthesizing sedimentary proxies with transient simulations, we identify a coherent, banded precipitation anomaly across the Asia-Pacific region that cannot be explained by regional dynamics alone. We show that seasonal deep convection over the IPWP excites planetary Rossby waves. On orbital timescales, their integrated effects are consistent with precessional forcing, while precession simultaneously modulates the large-scale atmospheric background state that influences their spatial organization. Guided by summer circulation, the Rossby-wave-related responses extend poleward and reorganize large-scale moisture transport and precipitation across the mid- and high-latitudes. The cumulative wave response and background-state modulation provide a robust dynamical linkage between tropical convection and extratropical hydroclimate on orbital timescales. This mechanism offers a physically consistent interpretation for how low-latitude orbital forcing imprints hydroclimate variability across Pan-Asia via planetary-wave dynamics.

## Abstract (web summary)

The study reveals that orbital-driven convection in the Indo-Pacific Warm Pool generates planetary Rossby waves. These waves propagate poleward, organizing a coherent, banded precipitation anomaly pattern across the Asia-Pacific region.

## Introduction

Global warming has altered the characteristics of hydrological circulation, leading to changes in regional precipitation patterns, frequency, and intensity^1^. A notable trend is the increase in precipitation over mid- to high-latitudes and tropical regions, while subtropical areas experience a decline in rainfall^1,2^. This phenomenon contributes to a scenario in which dry regions become drier and wet regions wetter^1,2^. The intensified contrast between dry and wet conditions may exacerbate imbalances in the global water cycle, leading to ecological degradation and an increase in extreme weather events, thereby threatening biodiversity and human livelihoods^3^. The future evolution of these hydroclimatic changes remains uncertain. Given modern climate observations spanning a limited timeframe, paleoclimate studies are essential for understanding the historical evolution, distribution, and underlying mechanisms of precipitation.

The Asia-Pacific region is recognized as one of the most prominent monsoon zones globally, exhibiting complex spatial heterogeneity in past precipitation changes^4^. For instance, a tripole precipitation pattern has been identified in eastern China during the last deglaciation, where the middle Yangtze River region exhibited distinct wet conditions compared to South and North China^5^. Furthermore, marine sedimentary proxies that reconstruct the historical South Asian summer monsoon and precipitation intensity lag local summer insolation by 8-9 kyr at the precession band^6,7^. In contrast, records of the East Asian summer monsoon precipitation, represented by Chinese stalagmites records, show a lag of 1 to 2 kyr relative to local summer insolation^4,8^. These spatial differences in precipitation may be attributed to land-sea thermal contrasts^4^, interactions between monsoon circulation and the westerly jet stream^5^, and/or variations in low-latitude moisture and energy transport^6^. However, a consensus on this issue has yet to be achieved. To better understand the spatial distribution of precipitation anomalies, it is essential to combine paleo-precipitation records with model simulations for comparative analysis.

**Fig. 1:**
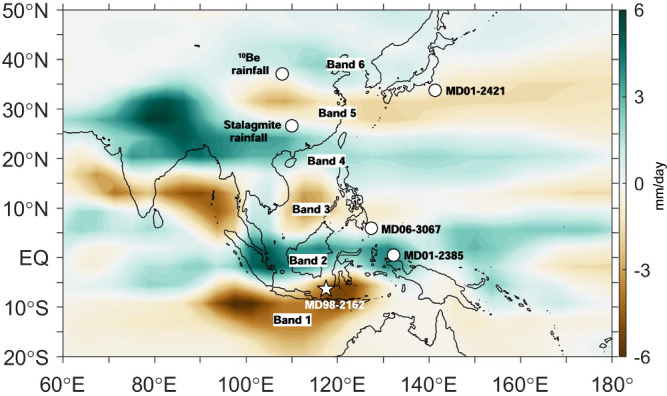
A hexapole pattern of precession-induced summer precipitation changes. Average summer (JJA) precipitation anomalies (mm/day) corresponding to the minimum minus maximum precession since 800 ka simulated by CESM 1.2 in the Asia-Pacific region. Band 1-6 indicate the hexapole banding areas. Stars and circles represent the locations of the paleo-precipitation records.

This study presents mineralogical evidence from core MD98-2162 south of the Makassar Strait to reconstruct the precipitation history on the adjacent islands over the past 150 kyr (Fig. 1). The weathering proxy of this core, derived from the key region of the Indo-Pacific Warm Pool (IPWP), differs from marine precipitation proxies in its controlling factors, thereby providing a new perspective on revealing the history of precipitation on surrounding islands^9^. We then analyzed precipitation results from transient model simulations and compiled a series of precipitation records spanning from low- to high-latitudes to more broadly assess the hydrological response to orbital forcing in the Asia-Pacific region (Methods, Figs. S1–4 and Table S1). Further, we use these results to develop a broader concept, whereby precession-regulated convection in the IPWP generates planetary-scale Rossby waves and associated circulation responses extending poleward, modulating the precipitation anomalies across the Asia-Pacific region.

## Results and discussion

### Reconstructed and modeled precipitation dynamics in the Asia-Pacific region

Clay mineral ratios are generally determined by lithology, climate-related weathering intensity, and sediment transfer^10^. Southeast Asian tropical islands are primarily composed of ‌mafic-to-intermediate volcanic rocks^11^. In the specific case of tropical islands weathering, ‌intense chemical weathering of basaltic rocks under warm, humid tropical conditions favors the formation of smectite‌, whereas ‌the enrichment of illite and chlorite suggests mechanical erosion of metamorphic and crystalline bedrock during arid intervals‌^9,10,12^. Studies combining proxy reconstructions and model simulations also support this conclusion by showing that variations in clay mineral composition of marine sediments in the Indo-Pacific region, especially the fluctuations in smectite, are directly related to the precipitation changes on the nearby volcanic islands^9,12,13^. Additionally, an increase in precipitation can enhance the weathering rate of the drainage and the sediment transport efficiency, thereby reducing the residence time available for intense chemical weathering^9,12,13^. This, in turn, facilitates the more efficient transportation of weakly weathered fine-grained clay minerals. Hence, clay minerals in core MD98-2162 could serve as robust proxies for precipitation-related weathering intensity on adjacent volcanic islands.

**Fig. 2:**
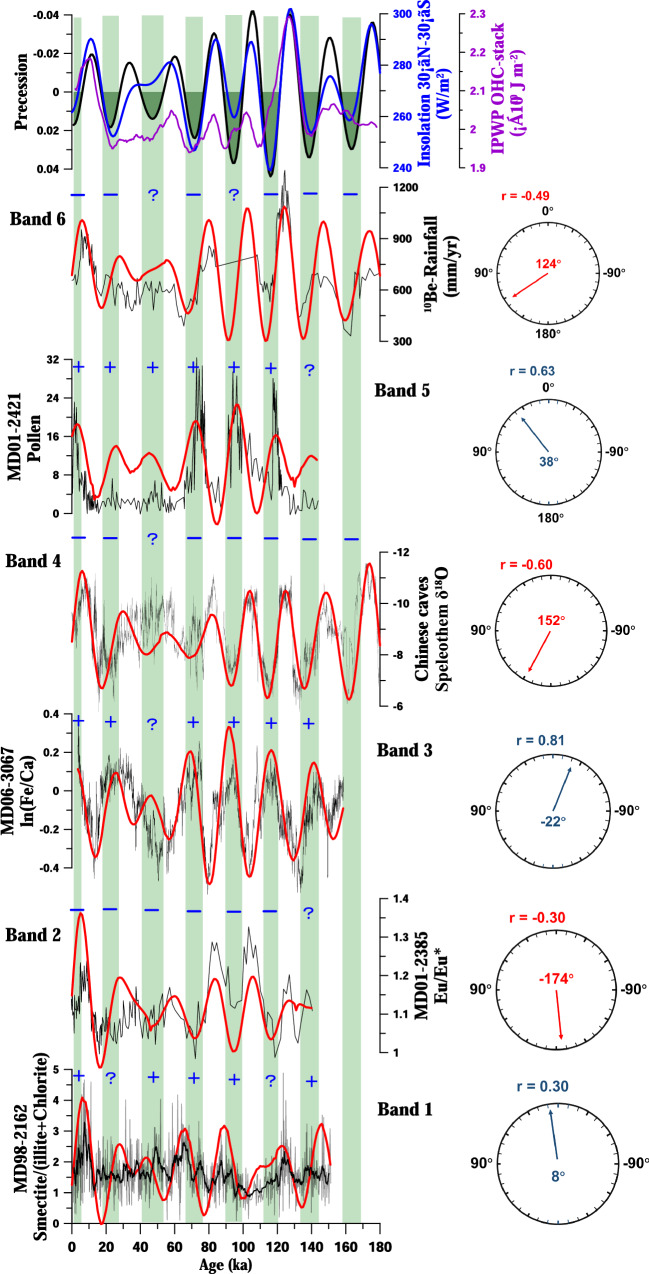
Comparisons of precession-paced precipitation changes from each Band. (1) Smectite/(illite+chlorite) ratios in MD98-2162 (this study); (2) Eu/Eu* ratios in MD01-2385^9^; (3) Ln(Fe/Ca) ratios in MD06-3067^58^; (4) Stalagmite δ^18^O from Sanbao Cave^8^; (5) Pollen from MD01-2421^59^; (6) ^10^Be of Chinese loess^17^, and (7) Stacked IPWP upper ocean heat content (OHC)^14^, insolation gradient from 30 °N to 30 °S and precession^15^. Note the precession axis is inverted. The red curves above each record are the filtered precession-cycle curves. The upward direction of y-axis indicates more precipitation for records in Bands 1-6. The degrees and r values noted on the phase wheel indicate the phase angle and correlation coefficient between the precession and each record during the 23-kyr cycle.

The smectite/(illite+chlorite) ratio in MD98-2162 shows higher values (more precipitation), generally corresponding to higher precession, lower IPWP upper OHC and interhemispheric insolation gradient (30°N-30°S; Fig. 2). Notable exceptions occur at ~25 and 115 ka, where obliquity minima (~29 and 111 ka) appear to decouple the expected precession-insolation relationship^14,15^. This observation is ‌exactly out of phase to the findings of previous studies^9,14,16^, which demonstrated that strong precipitation in the IPWP corresponds to lower precession, higher IPWP upper OHC and interhemispheric insolation gradient‌. Further, a combination of 18 precipitation records across the Asia-Pacific region reveals a distinct latitudinal banded pattern of hydroclimate responses (Methods, Figs. S1–4 and Table S1). Specifically, precipitation records from Bands 1, 3, and 5 roughly exhibit precipitation maxima during higher precession/lower IPWP upper OHC and interhemispheric insolation gradient intervals (Fig. 2 and S1), whereas Bands 2, 4, and 6 show opposite trends (Figs. 2 and S2–3). Although individual proxy indicators inherently exhibit distinct limitations, the coherent latitudinal progression of precession phases evident across 18 independent precipitation records (including marine sediments, speleothems, loess, and pollen sequences), collectively reinforce the credibility of our findings. We note that the phase-wheel diagram (Fig. 2) is intended to illustrate the broad pattern of precession-band phase relationships across records, rather than to support sub-kyr and/or precise interpretation of individual phase leads or lags. The general latitudinal trend in the composite appears consistent across multiple records, but the absolute timing of each record is subject to chronological uncertainties and site‑specific biases (Methods). Readers are cautioned against over‑interpreting small differences in phase angles.

In addition to the compiled records, transient simulations spanning the past 800 kyr (Methods) reveal a striking hexapole, latitudinally banded structure in summer precipitation anomalies across the Asia–Pacific region (60°–180°E, 20°S–40°N), characterized by alternating positive-negative phases with ~10°–15° latitudinal spacing (Fig. 1). The magnitude of summer precipitation anomalies in the extratropics (~6 mm/day) is comparable to the climatological mean (<6.75 mm/day; Fig. S1). This hexapole pattern agrees well with our proxy compilations (Figs. S2–3 and Table S1), validating the model’s ability to capture orbital-scale hydroclimate dynamics. Band-pass filtering of simulated summer precipitation indicates that precession predominantly dominates summer precipitation changes within the banded region, accounting for more than 80% of the variance (Fig. S5). The gradient of summer insolation between the two hemispheres is significantly anti-correlated with precession (r = -0.90, Fig. 2)^17,18^. In contrast, obliquity-driven summer precipitation anomaly exhibits an opposite phase and an amplitude of only 1/6 relative to that of precession (Fig. S6a, b), suggesting a relatively weak impact. Further, eccentricity also exerts weak amplitude modulation of precipitation anomalies within the banded domain (Fig. S6c). Our subsequent analysis, therefore, focuses on the physical mechanisms underlying precession’s control of the observed hexapole banding.

### Rossby wave impacts on band-shaped precipitation anomaly

Modern climate observations have identified a meridional tripole pattern in summer precipitation on an interannual timescale, which extends from the northwestern Pacific to eastern East Asia and is known as the Pacific-Japan (PJ) pattern^19^ (Fig. S7). Its dynamical mechanism is attributed to meridional Rossby waves excited by the convective anomalies over the western Pacific warm pool (10°N–20°N, 120°E–160°E). Within the Matsuno-Gill atmospheric response theory framework^20,21^, the latent heat release associated with deep convection in the warm pool is an effective thermal source that provides energy to the atmosphere. This process could subsequently induce anomalies in atmospheric geopotential height and ultimately excite Rossby waves with a typical symmetric structure on both sides of convective activity. These Rossby waves, coupled with moisture transport by the summer monsoon, collectively establish the PJ pattern^22,23^. In our preindustrial (PI) control simulation, summer precipitation anomalies during the last 100 years also exhibit characteristics closely resembling the modern PJ pattern (Fig. S7). The PI control also realistically reproduces the large-scale boreal summer monsoon circulation and precipitation: IPWP rainfall pattern agrees well with CMAP observations (Fig. S8), the 200 hPa subtropical jet (35–40°N, ~30–35 m/s), 850 hPa southwesterly monsoon flow, and the monsoon seasonal cycle are also well represented (Fig. S9), despite modest regional amplitude biases. These results further confirm the model’s capability to realistically simulate the band-shaped precipitation anomalies. However, whether precession-dominated precipitation banding anomalies are modulated by Rossby waves in a manner analogous to the PJ pattern remains to be rigorously verified.

**Fig. 3:**
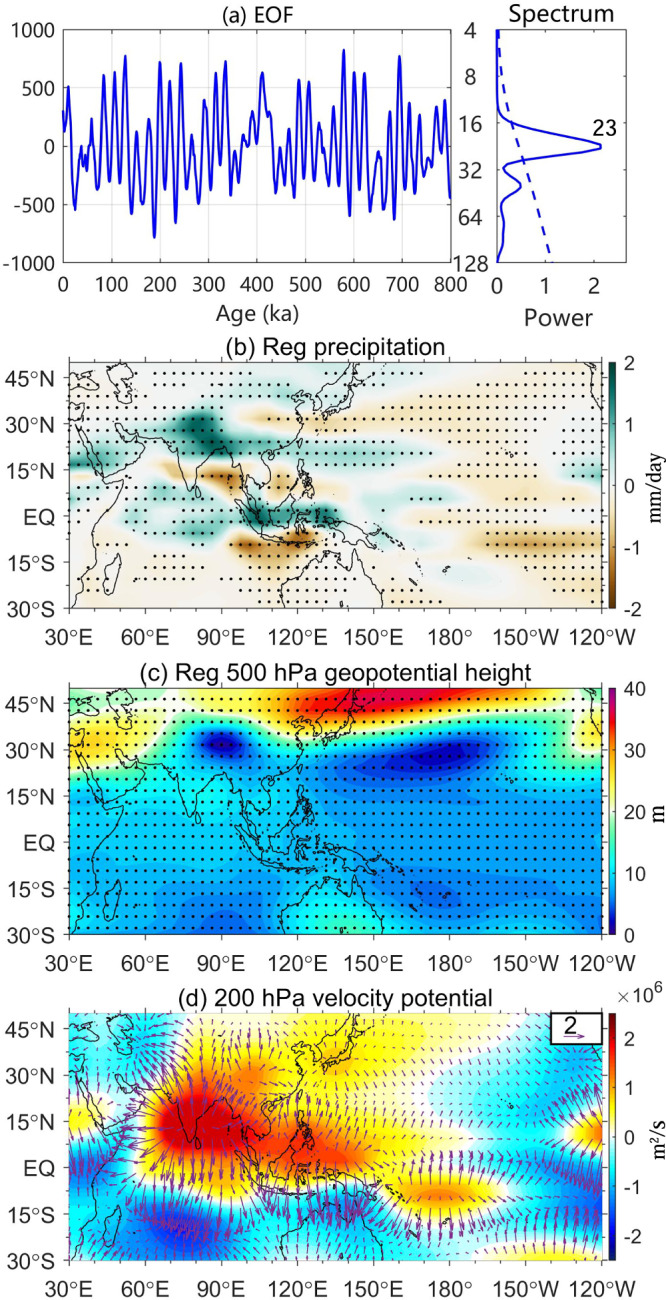
Precession impact on summer (JJA) precipitation and circulation changes since 800 ka. **a** Principal component (PC) timeseries associated with the first empirical orthogonal function mode of simulated JJA 500 hPa geopotential height anomalies and its power spectrum. Regressions of JJA **b** precipitation (mm/day), **c** 500 hPa geopotential height (m) and **d** 200 hPa velocity potential (shading; m^2^/s) and divergent winds (vectors; m/s) anomalies on the PC1 timeseries. The stippling in (**b**, **c**) indicate results that have passed the 95% confidence level.

To this end, we conducted empirical orthogonal function (EOF) analysis of the summer 500 hPa geopotential height anomalies in the mid-troposphere over East Asia (100°-130°E, 10°-50°N; the box area in Fig. S6a) since 800 ka (Fig. 3a). The 500 hPa level is selected as it best represents the large-scale atmospheric circulation while minimizing the influence of both the upper-level jet stream (200 hPa) and lower-level boundary layer processes (850 hPa). The results are not sensitive to the regional boundaries. Given that the Coriolis Force diminishes near the equator (e.g., between 5°S and 5°N), atmospheric dynamical processes (e.g., equatorial Kelvin waves) differ from those at mid-latitudes. To minimize the interference from equatorial dynamics and tropical ENSO signals, we restricted our EOF analysis to regions north of 10°N. The EOF analysis reveals that atmospheric circulation is predominantly explained by the first principal component (PC1), with a periodicity of 23-kyr and an explained variance of 86.5%. This mode shows a significant negative correlation with the precession parameter (r = -0.79). In contrast, the second mode contributes only 12.2% of variance (Fig. S10), indicating its relatively minor role.

Notably, regressing precipitation onto PC1 (Fig. 3b) reconstructs the characteristic hexapole banded pattern spanning 80°–180°E, 20°S–40°N, demonstrating dynamical coherence between extratropical circulation and tropical convection. Importantly, this banded precipitation structure is not an artifact of circulation-based regression: the leading EOF mode of precipitation anomalies alone exhibits an almost identical spatial pattern and temporal evolution (Figs. S10b, c), confirming that the coupled circulation–precipitation structure represents a physically coherent dominant mode of variability. This linkage persists despite our deliberate exclusion of equatorial regions to minimize interference from ENSO-like variability and equatorial wave dynamics. The result underscores a robust teleconnection between extratropical precipitation bands and the tropical IPWP, indicating that precession-driven precipitation anomalies are fundamentally governed by planetary-scale atmospheric circulation adjustment, not local processes.

The regressed 500 hPa atmospheric circulation on PC1 within 30°S-35°N exhibits a symmetrical distribution about the equator (Fig. 3c). This large-scale circulation pattern is characterized by elevated 500 hPa geopotential height over the IPWP, in contrast to regions between 20° and 30° latitude in both hemispheres. This elevated geopotential promotes the poleward transport of warm and moist air from low latitudes, enhancing moisture transport beyond 10°N. At the upper 200 hPa level, a distinct velocity potential maximum appears within this region (Fig. 3d). Notably, the velocity potential exhibits significantly greater magnitude in the Northern Hemisphere compared to the Southern Hemisphere. This asymmetry favors upper-level air divergence from the Rossby wave source region toward the polar region, while lower-level air converges in the opposite direction. Both the convective and divergent intensities are significantly greater in the Northern Hemisphere compared to the Southern Hemisphere. The interaction between lower-level convergence and upper-level divergence not only enhances local vertical convection, but also facilitates the efficient transport of low-latitude wave energy and moisture aloft towards mid- and high-latitudes. The enhanced poleward moisture transport reflects the strengthening of planetary-scale circulation, rather than local dynamics.

**Fig. 4:**
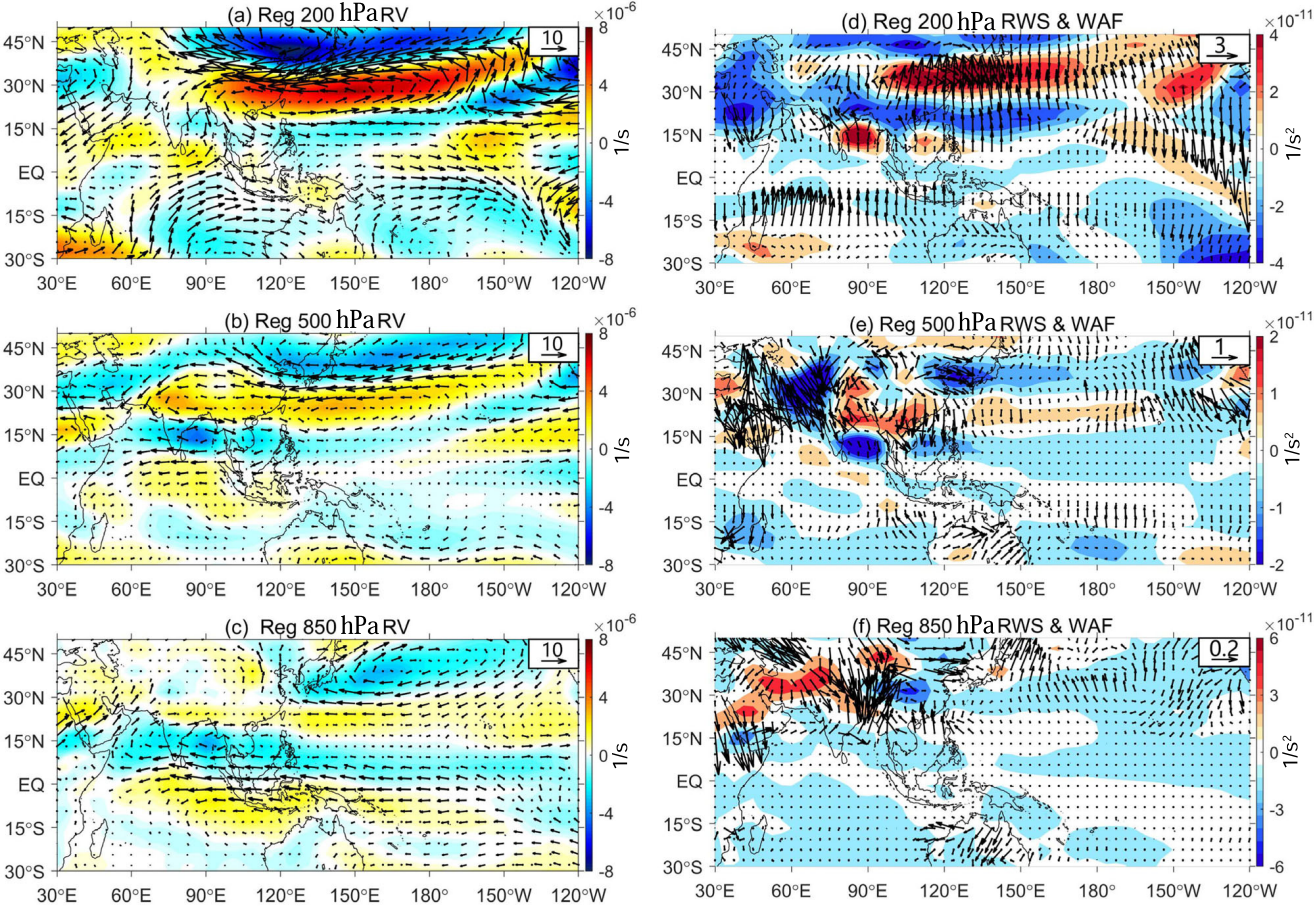
Rossby wave diagnoses simulated by transient CESM 1.2 since 800 ka. **a**–**c** Regression of JJA 200 hPa, 500 hPa and 850 hPa relative vorticity (RV, shadings; 1/s) and wind vector anomalies (arrows; m/s) on the normalized PC1, respectively. **d**–**f** Regression of JJA Rossby wave source (RWS, shading; 1/s^2^) and wave activity fluxes (WAF, arrows; m^2^/s^2^) at the same atmospheric levels. The stippling denotes values that are statistically significant at 90% confidence level.

Relative vorticity (RV) acts as an effective diagnostic proxy for Rossby wave phase characteristics and the rotational strength of large-scale atmospheric circulation, allowing the identification of Rossby wave phases within the circulation (Fig. 4a–c). Situated in the upper troposphere, the 200 hPa level is less influenced by friction compared to the 500 hPa and 850 hPa levels and more clearly expresses large-scale Rossby waves signals^24^. South of 40°N, the RV distribution closely aligns with banded precipitation anomaly patterns. Regions of positive RV are characterized by cyclonic (counterclockwise) circulation (arrows in Fig. 4a–c), corresponding to the trough phase of Rossby waves and coinciding with enhanced precipitation. Conversely, a negative RV is associated with anticyclonic circulation, ridge structures, and suppressed precipitation. This coherent correspondence indicates that the precession-driven precipitation pattern is strongly shaped by Rossby wave-mediated adjustments in the atmospheric circulation and associated large-scale meridional teleconnections. North of 40°N, a phase discrepancy is evident between RV and precipitation anomalies, indicating that the precipitation banding is no longer governed solely by Rossby waves emanating from the warm pool, but also impacted by midlatitude dynamics and thermodynamic factors, including the westerlies, the Western Pacific Subtropical High (WPSH), and land-sea thermal contrast^17^. Rossby wave activity is locally excited by anomalous deep convection over the IPWP, and disperses poleward within each hemisphere separately, without cross-equatorial propagation. The regulatory role of Rossby waves in shaping precipitation anomalies is not competitive with these potential influencing factors. The relationship between Rossby waves and additional climatic controls is demonstrated in Section 2.3.

The latitude-height composite of the regressed relative vorticity along the primary path of Rossby waves (110°-120°E) shows that the vertical gradient is concentrated within 5°S-5°N, indicating baroclinic influence; whereas poleward of 5°N, the weak vertical gradient suggests a dominance of barotropic effects (Fig. S11). The circulation pattern is consistent with Webster^25^. These results indicate that the interactions between barotropic and baroclinic mechanisms in atmospheric circulation jointly contribute to the excitation of Rossby wave activity and the development of large-scale meridional teleconnections, thereby facilitating the formation of band-shaped precipitation anomalies.

We further diagnose the Rossby wave source (RWS) and wave activity flux (WAF) to determine the wave source region and trace the meridional propagation of Rossby wave activity (Fig. 4d–f). Regressed RWS fields at both 200 and 500 hPa reveal a prominent positive RWS anomaly over the IPWP, including the Bay of Bengal, South China Sea, and Western Pacific. This coincides with the 200 hPa velocity potential maximum (Fig. 3d), identifying the IPWP as a key Rossby source region. Consistent with this diagnosis, WAF vectors clearly trace the meridional propagation of Rossby wave activity/energy emanating from this source into the Asia-Pacific region. A secondary positive RWS anomaly is evident near 30°N, consistent with the large RV values (Fig. 4a), suggesting further localized intensification and continued poleward extension of wave activity. Because the hexapole banding constitutes a regional, zonally inhomogeneous pattern with highly uneven spacing between its anomaly centers, it cannot be characterized by a single well-defined meridional wavenumber. Instead, it is best interpreted as a low-order, planetary-scale Rossby wave-related meridional teleconnection, consistent with the dominant large-scale meridional variability in the supplementary spectral analysis (Fig. S12). Overall, the combined RWS and WAF diagnostics reveal a coherent poleward-extending Rossby wave response, with its imprint on precipitation manifesting as six alternating precipitation anomaly centers spanning 20°S-50°N.

**Fig. 5:**
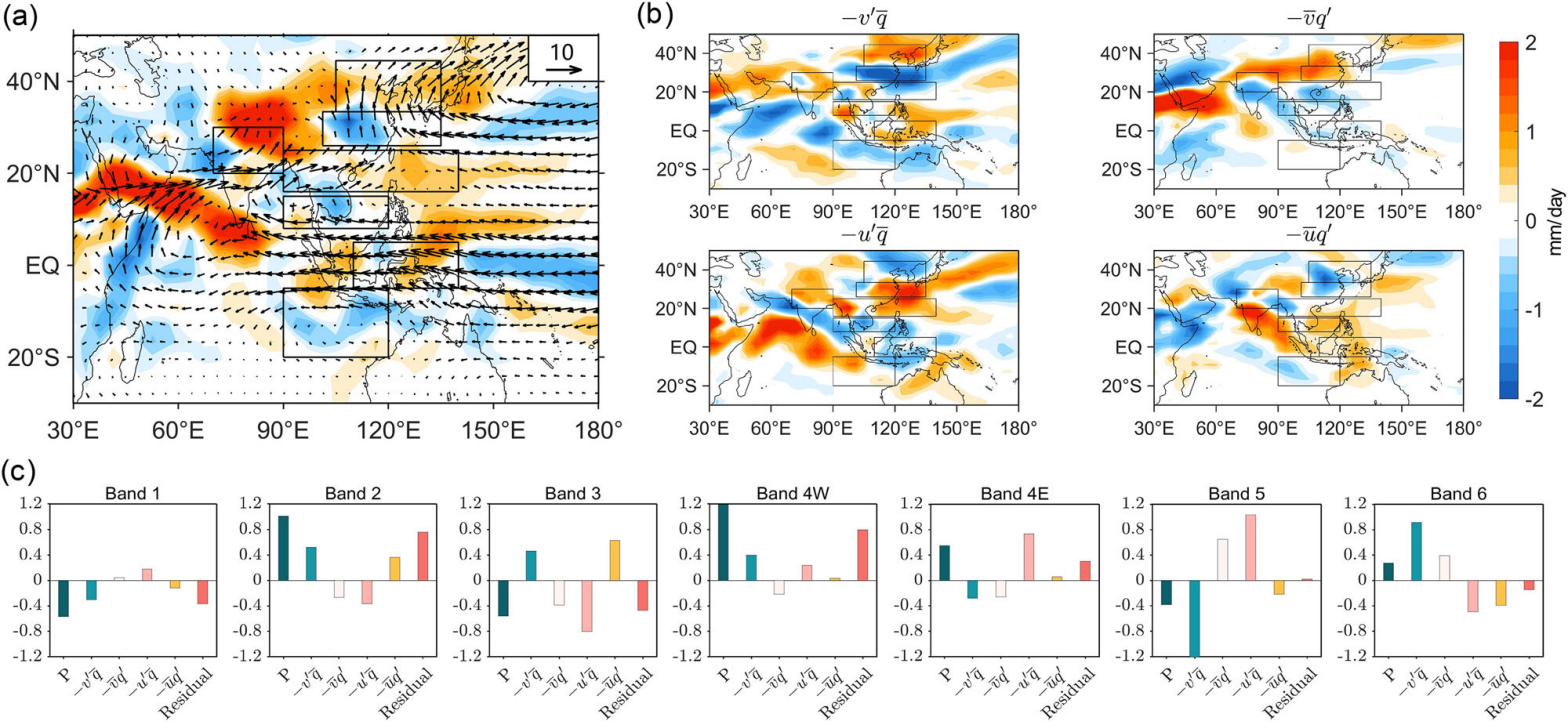
Water vapor budget diagnosis since 800 ka. **a** Regressed horizontal moisture fluxes (vectors: 10^6 ^kg/(m²·s)) and divergence (shadings; mm/day) onto the normalized PC1 during JJA. **b** The first four terms (mm/day) of Eq. (4) from water vapor budget analysis. **c** Area-averaged regression coefficients of each term in (**b**) over the six precipitation bands, with Band 4 further divided into western (Band 4 W) and eastern (Band 4E) sub-boxes. P and Residual in the *x* axis represent total precipitation and residual terms, respectively.

To assess how Rossby wave associated circulation anomalies modulate moisture transport and precipitation, we analyze vertically integrated moisture flux vectors and the corresponding moisture divergence fields (Fig. 5a). The spatial distribution of moisture convergence and divergence closely resembles the banding precipitation anomalies (Fig. 1). Pronounced moisture convergence is evident over the tropics (20°N–20°S; orange–red shading), indicating vigorous moisture accumulation and ascent associated with deep convection over the warm pool, which serves as the primary tropical moisture source. In contrast, extratropical regions—especially the subtropical high belt near 30°N—exhibit moisture divergence and subsidence, suggesting that precipitation anomalies there are remotely modulated by low‑latitude moisture rather than only local thermodynamic processes.

**Fig. 6:**
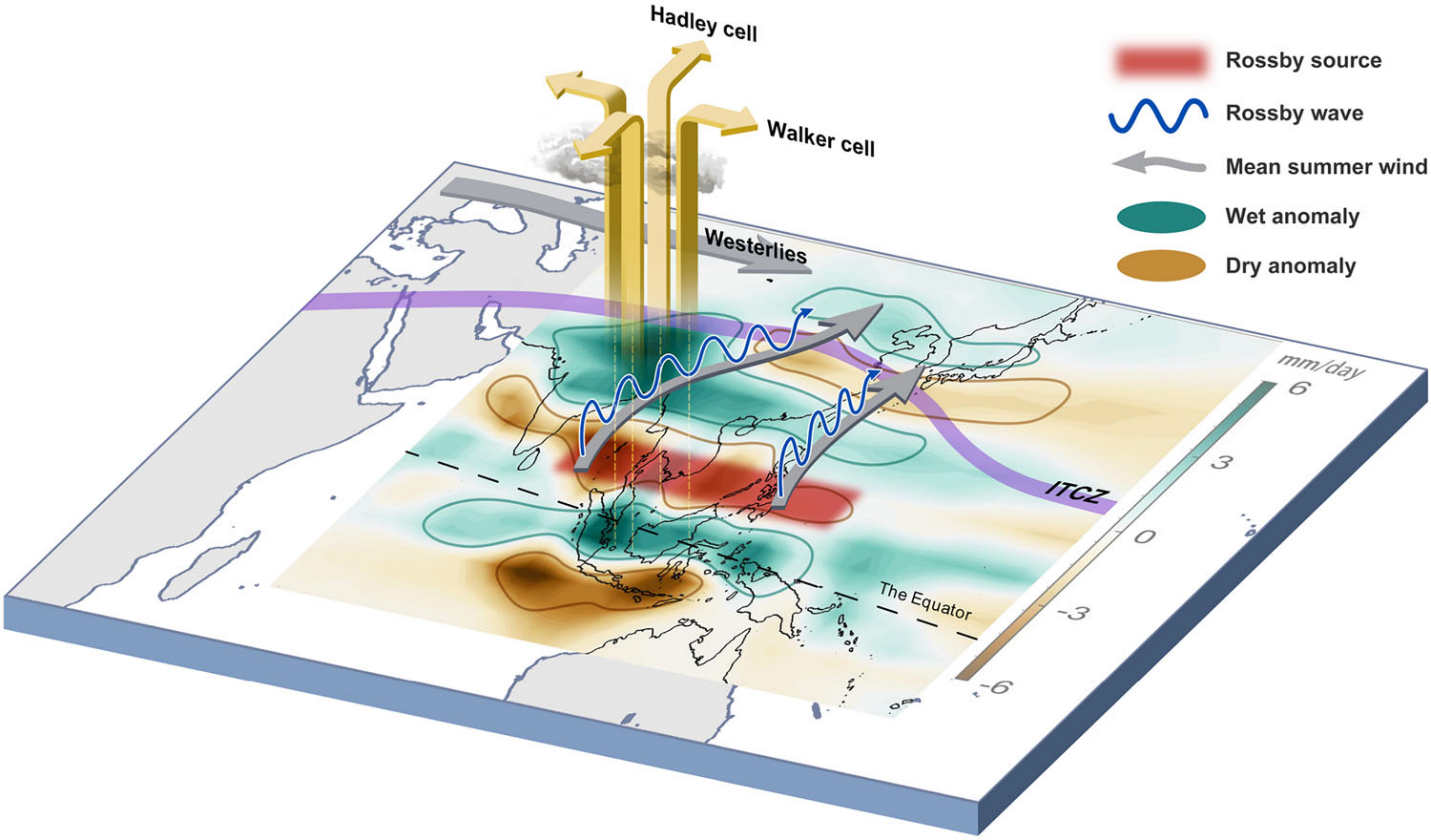
Schematic diagram of precipitation banding anomaly formation in the Asia-Pacific region. Precession-regulated insolation alters deep convection (gray clouds) over the IPWP, generating a Rossby wave source (red shading) and upper-tropospheric (200 hPa) divergence (yellow arrows). The resulting poleward extension of Rossby wave activity (blue curves) and moisture transport along the South Asian and western Pacific pathways (gray arrows) bridge tropical forcing to extratropical circulation, organizing coherent precipitation banding anomalies. This wave activity is locally excited by deep convection over the IPWP and disperses poleward within each hemisphere, with no cross-equatorial wave propagation invoked or implied. Only Northern Hemisphere results are presented here.

Along the monsoonal circulation, two distinct moisture transport corridors extending from the tropical Indian Ocean and western Pacific toward the mid-latitudes are identified (arrows in Fig. 5a), establishing a direct dynamical linkage between low‑latitude moisture sources and mid-latitude precipitation anomalies. These corridors are closely associated with pronounced vertical wind shear and vorticity anomalies (Fig. 4a–c), which favor the conversion of mean kinetic energy into perturbation kinetic energy — a key mechanism for the maintenance and poleward extension of Rossby wave activity into the mid-latitudes^26^. Consequently, moisture originating from the IPWP Rossby wave source is guided poleward by the southwesterly background flow in the Northern Hemisphere, primarily along the South Asian and western Pacific pathways (Fig. 6). Conversely, in the Southern Hemisphere, prevailing southeasterlies constrain low-latitude Rossby wave activity and its associated moisture transport largely to regions north of 20°S.

The moisture budget analysis further quantifies that the advective components associated with Rossby wave activity, namely the meridional (-${v}^{{\prime} }\bar{q}$) and zonal (-${u}^{{\prime} }\bar{q}$) moisture advection terms, are substantially larger than the thermodynamic contribution (-$\,\bar{u}{q}^{{\prime} }$ and -$\bar{v}{q}^{{\prime} }$), indicating that atmospheric circulation anomalies rather than humidity perturbations dominate moisture redistribution (Fig. 5b). In the tropics, meridional advection (-${v}^{{\prime} }\bar{q}$) is the leading term, reflecting efficient poleward export of moisture following low‑latitude convergence (Bands 1 and 2; Fig. 5c). In Bands 3 and 4E, zonal advection (-${u}^{{\prime} }\bar{q}$) becomes more prominent, consistent with monsoon-driven moisture accumulation within the tropical belt. This zonal moisture convergence sustains the banded tropical precipitation pattern and serves as a key wave source for the subsequent poleward extension of Rossby wave activity. In the mid‑ to high‑latitude bands (Bands 4 W, 5, and 6), Rossby wave-mediated meridional moisture advection (-${v}^{{\prime} }\bar{q}$) accounts for approximately 59%–89% of the total precipitation anomalies, suggesting that extratropical precipitation variability is largely governed by remote moisture transport. These results demonstrate that anomalous convection over the IPWP influences precipitation primarily by modulating Rossby wave-driven circulation and moisture pathways, rather than through direct local thermodynamic forcing. The poleward-extending Rossby wave response links tropical forcing to extratropical precipitation responses and ultimately shapes the large-scale spatial organization of precipitation anomalies across the Asia–Pacific region.

When modern climate models retain only Rossby source forcing in the IPWP, simulated results indicate that low-latitude Rossby wave energy can effectively disperse meridionally toward mid-latitude. However, upon removal of this source forcing, there is a dramatic decrease in amplitudes of these Rossby waves and the associated precipitation^27^. The comparison of these experiments underscores the pivotal role of the warm pool Rossby wave source in supplying the initial energy of Rossby waves. During strong Rossby wave phases, pronounced WAF propagation/dispersion along the subtropical waveguide accompanies clear, coherent banded precipitation anomalies across pan-Asia (Fig. S13). During weak phases, the wave structure and organized precipitation bands are largely suppressed. Quantitatively, precipitation anomalies in each band are strong and spatially coherent under active Rossby wave conditions but insignificant during inactive periods (Fig. S13g). This strong–weak phase contrast further demonstrates that reducing (or removing) Rossby wave activity leads to a collapse of the banded precipitation structure, which rigorously validates the essential role of Rossby wave activity in organizing the observed precipitation bands.

The banded precipitation pattern is not defined a priori, but each band is chosen to roughly enclose a coherent anomaly center within the alternating meridional pattern. To evaluate robustness to band definition, we perform two sensitivity tests: (i) shifting all bands meridionally by ±3.75° (one grid spacing), and (ii) applying 12° meridional smoothing (Figs. S14–15). The close correspondence between meridional moisture advection (-${v}^{{\prime} }\bar{q}$) and precipitation anomalies is preserved across both tests, indicating the core dynamical linkage is insensitive to reasonable variations in band definition. Area-mean amplitude sensitivity in Bands 5–6 arises from their location within strong meridional anomaly gradients, where small shifts cause partial cancellation of opposite-signed signals (Fig. S15c and e). The simultaneous weakening of precipitation and -${v}^{{\prime} }\bar{q}$ reflects spatial sampling of a continuous meridional structure, not a breakdown of the underlying mechanism. These results demonstrate that the banded pattern is a robust, large-scale dynamical structure driven by Rossby wave-induced moisture transport, not an artifact of band definition. The six-band configuration is thus a convenient discretization of a continuous meridional mode, and the associated dynamical interpretation are independent of exact band boundary choices.

Based on the aforementioned results, we propose that the precession-controlled hexapole latitudinal banding of summer precipitation anomaly could be interpreted as an atmospheric circulation adjustment associated with Rossby wave activity and wave energy dispersion (Fig. 6). Anomalous convective activity in the IPWP is inferred to act as a Rossby wave source, generating two Rossby wave-related upper-level responses extending poleward along the South Asian and western Pacific corridors, guided by the summer monsoon circulation. Through their influence on large-scale circulation and moisture transport, this Rossby wave activity appears to modulate the redistribution of subtropical moisture and contribute to the organization of latitude-dependent precipitation bands (Fig. 5).

In contrast to invoking long-period Rossby waves operating directly at orbital timescales^28^, we focus on fast, seasonal-scale atmospheric Rossby waves that are repeatedly excited by tropical convection and accumulate over orbital timescales through stable spatial superposition, as supported by both paleoclimate reconstructions and transient simulations in this study. Precession forcing does not directly govern the fast dynamics of Rossby waves. Instead, it indirectly modulates their generation and intensity by regulating IPWP deep convection and upper-ocean heat content. Consequently, the observed hexapole precipitation pattern emerges from precession-dominated insolation forcing and the dominant organizing role of Rossby wave dynamics, which bridge low- to high-latitude hydroclimate via moisture transport and dynamically reinforce zonal precipitation banding. Previous paleoclimate records and advanced modeling studies have documented the linkage between insolation and precipitation via Rossby waves on millennial to centennial timescales^29^. This complements our study, which spans orbital to modern periods, including preindustrial and current PJ patterns, and thereby establishes a coherent linkage between insolation variability, Rossby wave dynamics, and precipitation anomalies across timescales.

This interpretation differs fundamentally from resonant oceanic gyral Rossby waves that mediate orbital signals via long-period feedbacks^28,30^. Atmospheric and oceanic wave processes act on distinct timescales within different components of the climate system, rendering them complementary rather than contradictory. Together, they highlight the diverse roles of wave dynamics in translating orbital forcing into regional hydroclimate variability.

### Potential climatic drivers on band-shaped precipitation anomalies

To assess the potential climatic drivers of the banded precipitation anomalies over the Asia–Pacific region, we extracted long-term variability of several key climate components over the past 800 kyr (Fig. S16), including the Intertropical Convergence Zone (ITCZ), Hadley cell strength, the WPSH, and the South Asian monsoon (SAM; see Methods). We do not separately analyze the Walker circulation or the East Asian monsoon, as both are tightly coupled to the ITCZ^31^. Variations in the latitudinal position of ITCZ and WPSH, together with the SAM index, are dominated by precession (Fig. S16), consistent with the leading PC1 of the simulated atmospheric circulation. In contrast, precession exhibits as a secondary period in the strength of the Hadley circulation in both the Northern and Southern Hemispheres. Importantly, these circulation features primarily influence regional precipitation and lack a clear mechanism for producing the spatially coherent, latitudinally banded precipitation anomalies extending across the pan Asia-Pacific region^32,33^. We therefore interpret these components as regional modulators, rather than primary drivers of the observed hexapole precipitation pattern.

The Rossby waves propagate northward, guided by the large-scale atmospheric circulation, but encounter obstructions due to continental topography. The Asian continent exerts significant blocking and friction effects on atmospheric fluctuations, which can lead to the breaking of the propagating Rossby waves^34^. This breaking results in a reduction of wave scales and produces a banded pattern of precipitation anomaly across continental regions between 10°N and 50°N. In contrast, in the Southern Hemisphere, the reduced land area and persistent southerly winds act as barriers, limiting the southward propagation distances of Rossby waves generated at warm pool region. Consequently, no significant banded pattern in precipitation anomaly is observed south of 20°S.

The land–sea thermal contrast is a fundamental control on the mean state of regional precipitation and monsoon circulation^4^. However, this mechanism cannot adequately explain the anomalous latitudinal banding of summer precipitation here, particularly considering the absence of analogous banded patterns in other meteorological fields, including surface pressure, geopotential height, and wind (Figs. 3–4). Rather than directly forcing the precipitation anomalies, the land–sea thermal contrast primarily establishes the ‌background thermodynamic and circulation state‌ within which anomalies develop, regulating tropical convection and the large-scale monsoon circulation^35^. Our analysis instead focuses on the ‌summer precipitation anomaly field, ‌defined by the difference between minimum and maximum precession. Against this background mean state, we propose an atmospheric waveguide mechanism‌ in which precession-modulated convection in the IPWP excites planetary-scale Rossby wave activity. This Rossby wave-mediated circulation response extends poleward through the summer atmospheric waveguide, redistributes moisture and dynamically organizes the observed banding precipitation anomalies.

The banded precipitation anomaly is absent in winter (Fig. S17), indicating strong seasonal constraints on the underlying dynamics. Two factors jointly suppress the formation of wintertime precipitation banding. First, the winter background circulation is unfavorable for the poleward extension of Rossby wave activity. Although extratropical Rossby waves may still be generated in winter, the prevailing circulation, characterized by a strengthened zonal flow, does not provide an effective meridional waveguide linking the tropics and extratropics, in contrast to the summer circulation. Second, tropical thermodynamic forcing is significantly weakened in winter. Precession-driven tropical insolation is significantly lower than in summer^18^. Composite analyses show widespread negative precipitation anomalies over the IPWP, suggesting suppressed deep convection and a weakened Rossby wave source.

Consistent with these constraints, diagnosed winter RWS anomalies are weak and fragmented near 30°N, and WAF vectors indicate predominantly zonal energy propagation, effectively suppressing meridional wave propagation (Fig. S18). Additionally, weak low-level (850 hPa) RV anomalies and reduced moisture transport prevent the downward translation of upper-level wave signals to surface precipitation responses. Consequently, despite the presence of mid-latitude Rossby wave activity, coherent latitudinal precipitation banding across the pan-Asian-Pacific is strongly weakened in winter.

Notably, the role of Rossby waves in shaping precipitation anomaly appears to be organizing, rather than competing with, and thus complementary to other climate factors considered in our study. The hexapole banded structure in both proxy records and model simulations is consistent with the large-scale adjustment and spatial organization of precipitation anomalies associated with Rossby wave dynamics. By modulating atmospheric circulation and moisture transport pathways, Rossby waves provide a dynamically consistent mechanism linking regionally distinct precipitation responses into a coherent latitudinal banding pattern. The absence of Rossby wave-mediated moisture transport is associated with substantially reduced mid-latitude precipitation^27^ and a greater confinement of precipitation anomalies to low-to-subtropical regions.

The model used in this study realistically captures large-scale features (subtropical jet, monsoon seasonal cycle, moisture transport). Regional precipitation biases and LGM boundary conditions may affect local details, but not the large-scale coupling among tropical heating, Rossby wave activity, and meridional moisture transport.

## Implications

Both the modern PJ tripole pattern and the orbital-scale hexapole system appear to be closely linked to anomalous tropical insolation forcing. This forcing is inferred to modulate the Rossby wave source function and the poleward extension of Rossby wave activity, establishing teleconnections that shape precipitation anomalies in the mid- and high-latitudes. On orbital timescales, the precipitation banding anomalies over the Asia-Pacific exhibit spatial characteristics largely consistent with those of both the pre-industrial and the modern PJ patterns (Fig. 1 and S7). This consistency suggests that the modern PJ pattern is a spatially truncated manifestation of a broader, planetary-scale precipitation organization operating at orbital timescales under the modern climate state. When Rossby wave activity excited by tropical thermal forcing extends poleward, its pathways are further modulated by the summer monsoon circulation and other background atmospheric flows, contributing to distinct precipitation characteristics through interactions with other climatic factors. This integrated mechanism offers several key advances. First, it provides a dynamical perspective on the persistence of the PJ pattern across interannual to decadal timescales, consistent with its observed modal stability. Second, it offers a physically consistent interpretation for spatial heterogeneities in paleo-precipitation reconstructions across the Asia-Pacific, including the tripole pattern over eastern China during the last deglaciation^5^. Third and critically, orbital-scale precipitation banding anomalies may serve as a physically grounded reference for natural PJ-type variability, which is essential for assessing the contribution of anthropogenic warming to the intensity and frequency of recent and future PJ-related extreme events.

## Methods

### Clay mineralogy in core MD98-2162

Core MD98-2162 (4°41.33′ S, 117°54.17′ E, 1855 m water depth) was collected from the Makassar Strait, inside the IPWP, during the International Marine Global Changes Study cruise in June 1998. This core is composed of uniform hemipelagic mud rich in foraminifera and nannofossil oozes, without obvious turbidites or volcanic ash layers. Its age model was established based on 12 accelerator mass spectrometry (AMS) ^14^C dates on planktic foraminiferal *G. ruber* for the upper 40 ka^36^. The age between 150 to 40 ka was built by tuning planktic foraminifera *G. ruber* δ^18^O to the *G. ruber* δ^18^O of core MD01-2378, whose age is based on the synchronization of its benthic foraminifera δ^18^O record to the LR04 reference curve^14^.

We analyzed clay mineralogy on a total of 830 samples with an average interval of 4 cm, corresponding to an average resolution of ∼200 years. Marine sediment samples were treated and analyzed following the method described previously^37^. Briefly, the organic matter and carbonates in the samples were completely removed using acetic acid (25%) and hydrogen peroxide (15%), respectively. After repeated ultra-pure water rinsing, clay minerals with grain size <2 μm were extracted based on the Stoke’s settling velocity principle. Clay minerals were identified by X-ray diffraction (XRD) using a D8 ADVANCE diffractometer with CuKα (alpha) radiation (40 kV, 40 mA) in the Laboratory of the Institute of Oceanology, Chinese Academy of Sciences (IOCAS). Three XRD runs were performed after air-drying, ethylene glycol solvation for 24 h, and heating at 490 °C for 2 h.

### Compilation of precipitation records in Asia-Pacific region

We have compiled 18 published paleo-rainfall reconstructions across the Asia-Pacific region at different latitudes (Supplementary Table 1). The selection criteria are as follows: (1) Continuous records, covering at least the last interglacial (since ∼150 ka) with sufficient chronology and temporal resolution to resolve the orbital-scale cycles of precession; (2) Sensitive to the hydrological cycle, interpreted as reliable precipitation records; (3) Covering the Asia-Pacific region from low- to high-latitudes. All data were first linearly interpolated to a 1-kyr resolution, and then precession-cycle bandpass filtering was applied using PAST software (Figs. S2–3). The filtering frequency is from 0.033 to 0.053, with bandwidth = 0.02 and transition = 0.005. Correlation analysis was conducted using the Monte Carlo method to verify the relationship between the precipitation records and the precession (Fig. S4). The correlation coefficients and their standard errors between the filtering curve for the controlling cycle of each compiled precipitation record and precipitation were calculated. The Gaussian distribution is used to fit each expected probability density. The correlation coefficients and their standard errors are also labeled.

Displaying all 18 individual records in the main text is impractical, and mechanically averaging records with different inherent uncertainties is not methodologically sound. Therefore, for each of the six Bands, we have selected ‌one representative record with the highest temporal resolution and most robust chronology‌ for presentation in the Figs. 1 and 2, while the remaining records were kept in the Supplementary Figs Note that the records from the same band were generally consistent (Figs. S2–3).

### Modern observational and reanalysis data

Monthly precipitation is taken from the Climate Prediction Center Merged Analysis of Precipitation (CMAP) dataset^38^, which merges gauge and satellite observations onto a 2.5° grid for 1979–present. It is used to characterize large-scale meridional banded precipitation anomalies.

Atmospheric fields (winds, humidity, geopotential height) are from ERA5 reanalysis^39^, provided by ECMWF at 0.25° resolution with 37 pressure levels. ERA5 is employed to diagnose circulation, moisture transport, and Rossby wave dynamics, owing to its high accuracy and dynamical consistency.

Topographic data were derived from the ETOPO global relief model (NOAA National Centers for Environmental Information, NCEI), which provides integrated global land topography and ocean bathymetry datasets.

### Transient paleoclimate model simulation

The used model of CESM version 1.2 (CESM1.2) was forced by transient orbital forcing, ice-sheet forcing, and CO_2_ forcing^40,41^. This model contains fully coupled components, including the atmosphere (Community Atmosphere Model version 4 with ~3.75° horizontal resolution, 26 vertical levels, model top at 2 hPa), the ocean (Parallel Ocean Program version 2 with ~3° horizonal resolution, 25 depths), sea ice (Los Alamos Sea Ice Model), and land (Community Land Model version 4 with prescribed vegetation). The output data of the atmosphere and ocean are regridded at a horizontal resolution of 3.75° × 3.75° (T31 resolution), with an interval of 1000 years.

Following the previous work^40^, we provide a description of the external forcings and boundary-condition updates applied in the 3 Myr CESM1.2 transient simulation. Ice-sheet geometry and topography were prescribed from time-varying CLIMBER-2 simulation^42^ and remapped to the CESM grid at 100-yr intervals. The model did not include the ice-sheet freshwater forcing or calving from ice-sheets. This is because freshwater forcing originating from the ice sheets could possibly lead to an underestimation of the simulated Atlantic Meridional Overturning Circulation (AMOC) variability on orbital timescales. The land-sea mask and coastline are kept fixed at the Last Glacial Maximum condition. The 800 kyr Greenhouse-gas concentrations (CO₂, N₂O, CH₄) are prescribed based on the EPICA observations^43^. To get a reliable climate sensitivity, the CO_2_ forcing is scaled by a factor of 1.5. Greenhouse-gas forcing, Northern Hemisphere ice-sheet forcing, and orbital parameters^44^ are updated every 100 model years. The model applied a relatively small orbital acceleration factor of 5 (for orbital, Greenhouse-gas, and ice sheet topography forcings), reducing orbital 3 Myr into 600,000 CESM model years. The simulation was initialized from a fully spun-up 2300-yr preindustrial control run with minimal drift, ensuring numerical stability.

Although previous work had already shown a reasonable agreement of simulation in comparison with the proxy data by this model, including the large-scale monsoon and orbitally paced precipitation changes^40^, we further compared the last 100-yr preindustrial control (PI control; fixed 285 ppm CO_2_, present-day orbital and geometry, but the land-sea mask fixed at LGM) with modern observed PJ to demonstrate that the model realistically captures precipitation changes, large-scale monsoon dynamics, seasonal cycle of circulation, and Rossby wave teleconnections (Figs. S7–9). Vertically integrated moisture transport magnitude and pathways are also broadly consistent with ERA5 (Fig. S19). While modest biases exist and an LGM land–sea mask is employed, the large-scale dynamical features critical to Rossby-wave propagation and moisture–circulation interactions are faithfully captured.

Several limitations of the experimental design should be considered. The absence of freshwater forcing from ice sheets may affect AMOC variability^45,46^, while the use of a fixed LGM land–sea mask and prescribed ice-sheet geometry may influence regional moisture pathways and precipitation intensity^47,48^. However, these factors primarily affect the amplitude and regional expression of precipitation anomalies. The large-scale atmospheric circulation and planetary wave responses identified in this study are mainly controlled by tropical heating and background circulation, and are therefore expected to be robust. Consequently, these limitations do not qualitatively alter the dynamical mechanism proposed here, although regional details should be interpreted with caution.

### Atmospheric indices

We have analyzed hydroclimate variability over the past 800 kyr in the Asia-Pacific region with a transient paleoclimate model. Band-pass filtering was adopted on the simulated summer precipitation to isolate the effects of individual orbital parameters on this hexapole pattern (Fig. S6), with periods of 17-28 kyr for precession, 37-45 kyr for obliquity, and 96-104 kyr for eccentricity. Our results are not sensitive to slightly broader and narrower filter windows. We also analyzed the variability of several well-established climatic indices to assess their possible relationships with band-shaped precipitation anomalies (Fig. S16). The ITCZ migration was defined by the latitude of maximum zonal-mean precipitation between 10°S–10°N^49^. Hadley cell strength was quantified by meridional mass flux integrated across 1000–200 hPa between 0°–30°N/S^50^. The South Asia Monsoon index was defined by standardized PC1 of summer precipitation over the South Asian monsoon domain (70°–90°E, 10°–30°N)^51^. The position of the Western Pacific Subtropical High (WPSH) was determined by the latitude of the 500 hPa geopotential height maximum over 120°–150°E, 20°–40°N^52^. Statistical significance tests were conducted using two-tailed Student’s *t*-test. Model output and figures were generated with MATLAB (R2021b), and all filtering was performed using a Lanczos filter^53^.

### Atmospheric wave activity flux and Rossby wave source

In this study, the horizontal propagation features of quasi-stationary Rossby waves are characterized by the atmospheric wave activity flux^54^. Under the quasi-geostrophic approximation, the horizontal component of the wave activity flux in the pressure coordinate system is formulated as:

1 $$\vec{W}=	 \frac{p\cos \varphi }{2\left|\vec{U}\right|}\left( {\frac{U}{{r}^{2}{\cos }^{2}\varphi }}\left[{\left(\frac{\partial {\psi }^{{\prime} }}{\partial \lambda }\right)}^{2}-{\psi }^{{\prime} }\frac{{\partial }^{2}{\psi }^{{\prime} }}{\partial {\lambda }^{2}}\right] \right.\\ 	 \left. {+\frac{V}{{r}^{2}\cos \varphi }\left[ \frac{\partial {\psi }^{{\prime} }}{\partial \lambda }\frac{\partial {\psi }^{{\prime} }}{\partial \varphi }-{\psi }^{{\prime} }\frac{{\partial }^{2}{\psi }^{{\prime} }}{\partial \lambda \partial \varphi }\right]}{\frac{U}{{r}^{2}\cos \varphi }\left[\frac{\partial {\psi }^{{\prime} }}{\partial \lambda }\frac{\partial {\psi }^{{\prime} }}{\partial \varphi }-{\psi }^{{\prime} }\frac{{\partial }^{2}{\psi }^{{\prime} }}{\partial \lambda \partial \varphi }\right]+\frac{V}{{r}^{2}}\left[{\left(\frac{\partial {\psi }^{{\prime} }}{\partial \varphi }\right)}^{2}-{\psi }^{{\prime} }\frac{{\partial }^{2}{\psi }^{{\prime} }}{\partial {\varphi }^{2}}\right]}\right)$$

Where p represents the normalized atmospheric pressure (pressure divided by 1000 hPa), r is the Earth’s radius (6.37×10^6 ^m), λ and$\,\varphi$ refer to longitude and latitude, respectively, $\vec{U}=(U,\,V)$ denotes the climatological horizontal winds, and the prime indicates anomalies from climatological means. Under the quasi-geostrophic assumption, the stream function anomaly ${\psi }^{{\prime} }$ is derived from the ratio of geopotential anomaly ${\phi }^{{\prime} }$ to the planetary vorticity (f).

The Rossby wave source (RWS) was applied to diagnose the Rossby wave sources excited by atmospheric convection in the warm pool^55^. The RWS is defined as:

2 $$\begin{array}{c}\,{RWS}=-{\nabla }_{H}\cdot \left\{{V}_{\chi }^{{\prime} }\left(f+\bar{\zeta }\right)\right\}-{\nabla }_{H}\cdot \{\bar{{V}_{\chi }}{\zeta }^{{\prime} }\}\\ \,\end{array}$$

Here, f and$\,\,\zeta$ denote planetary vorticity and relative vorticity, respectively. ${V}_{\chi }$ represents the divergent component of the horizontal winds, and ${\nabla }_{H}$ signifies the horizontal gradient. Overbars indicate the climatological mean, while primes denote anomalies.

### Moisture budget

Moisture budget analysis was performed to quantify the relative contributions of various moisture transport processes for summer (JJA) precipitation anomalies. The moisture budget equation is expressed as:

3 $$P=-\frac{1}{{\rho }_{w}g}{\int }_{{P}_{t}}^{{P}_{s}}\left[\nabla \cdot \left(\bar{V}{q}^{{\prime} }\right)\right]{dp}-\frac{1}{{\rho }_{w}g}{\int }_{{P}_{t}}^{{P}_{s}}\left[\nabla \cdot \left({V}^{{\prime} }\bar{q}\right)\right]{dp}+E+R$$

Here, *ρ*_w_ is the density of liquid water, *V*  =  (*u, v*) represents the horizontal wind vector, *q* is the specific humidity, *g* is the gravitational acceleration, *Ps* is surface pressure, and *Pt* is the top of the atmosphere pressure, set to 100-hPa in this study. *P* and *E* denote total precipitation and surface evaporation, respectively, while *R* represents the residual term.

The first two terms on the right-hand side quantify moisture convergence due to mean circulation transporting anomalous moisture ($\bar{V}{q}^{{\prime} }$) and anomalous circulation transporting mean moisture (${V}^{{\prime} }\bar{q}$), commonly referred to as thermodynamic and dynamic contributions, respectively. The residual term *R* encompasses effects from the vertical motions at the surface and tropopause, moisture storage, and high-frequency eddy fluxes, which are assumed negligible at orbital timescales.

To further isolate zonal and meridional contributions, the horizontal wind field *V* is decomposed into zonal and meridional components (i.e., *V*  =  *(u, v)*), yielding:

4 $$\begin{array}{c}P=-\frac{1}{{\rho }_{w}g}{\int }_{{P}_{t}}^{{P}_{s}}[\nabla \cdot (\bar{u}{q}^{{\prime} }\, )]{dp}-\frac{1}{{\rho }_{w}g}{\int }_{{P}_{t}}^{{P}_{s}}[\nabla \cdot (\bar{v}{q}^{{\prime} })]{dp}\\ -\frac{1}{{\rho }_{w}g}{\int }_{{P}_{t}}^{{P}_{s}}[\nabla \cdot (u^{\prime}\, \bar{q})]{dp}-\frac{1}{{\rho }_{w}g}{\int }_{{P}_{t}}^{{P}_{s}}[\nabla \cdot ({v}^{{\prime} }\bar{q})]{dp}+E+R\end{array}$$

The spatial patterns of the moisture convergence and four budget terms are presented in Fig. 5a-b, and their quantitative contributions of each budget term are shown in Fig. 5c. Evaporation (*E*) is not the dominant factor in determining long-term precipitation anomalies and is therefore not shown.

### Projection of Rossby-wave activity

To quantify the amplitude of the upper-tropospheric Rossby wave pattern, we construct a projection index by projecting the anomalous geopotential height onto a reference Rossby wave pattern^56,57^. The reference pattern is defined as the regression of 200 hPa geopotential height onto the precession index (the dominant wave mode linked to IPWP convection). Projections are performed over the subtropical waveguide region (15°–50°N, 60°–180°E), with cosine latitude weighting:

5 $$I\left(t\right)=\frac{{\sum }_{x,y}Z^{\prime} (x,y,t){Z}_{{ref}}(x,y)\cos \phi {{\varnothing }}}{{\sum }_{x,y}{{Z}_{{ref}}(x,y)}^{2}\cos \phi }$$

where $Z^{\prime}$ is the geopotential height anomaly, ${Z}_{{ref}}$ is the reference pattern, and *ϕ* is the latitude. The index is normalized by its standard deviation. This index provides a measure of the amplitude of the associated wave response. Strong and weak Rossby wave phases are defined as periods when the normalized index exceeds ±1 standard deviation and lies within ±0.5 standard deviation, respectively.

### Estimation of errors

We take the six precipitation records shown in Fig. 1 as an example to provide a detailed estimation of errors. For smectite/(illite+chlorite) ratios in core MD98-2162, the analysis uncertainties were ~5% (1σ). The measurement uncertainties for Eu/Eu* ratios in MD01-2385 and stalagmite δ^18^O from Sanbao Cave are generally less than 1% (1σ)^8,9^. For Ln(Fe/Ca) ratios in MD06-3067, the analysis uncertainties were ~5% (1σ)^58^.The uncertainties of pollen from MD01-2421 are not reported^59^. As such, we arbitrarily assigned a 5% (1σ) uncertainty to this record. The average uncertainties for ^10^Be of Chinese loess were suggested to be ~9.4% (1σ)^17^. Apart from the analytical uncertainties of the proxies, the chronology of the archives also introduces errors. Stalagmite δ^18^O from Sanbao Cave is absolutely dated by U/Th method with errors that can be ignored^8^. For AMS ^14^C dates, the errors in age measurement can range from a few years to several hundred years^60^. The uncertainties of tuning are ~2-5 kyr for the last 1 Ma, which should be smaller than 2 kyr in the last 160 ka for our study^61^. Monte Carlo method was applied to calculate the correlation coefficients and their standard errors between filtering curve of each compiled precipitation record and precession (Fig. S4). The absolute values of correlation coefficients are generally between 0.3 and 0.8, with a *p*-value lower than 0.01 (Fig. S4). Gaussian fitting also indicates that these error distributions are basically in accordance with the normal distribution (Fig. S4). When all sources of uncertainty are combined, we estimated average total uncertainties for paleo-precipitation records are better than 10% (1σ). Such uncertainties generally meet the requirements of orbital study.

## Supplementary information

Supplementary Information

Description of Additional Supplementary Files

Supplementary Data 1

Supplementary Data 2

Transparent Peer Review file

## Data Availability

Clay mineral data is available in the supplementary Material. The transient paleoclimate simulation data is available on the ICCP climate data server: https://climatedata.ibs.re.kr. ETOPO1 topographic data were derived from: https://www.ncei.noaa.gov/.
