# Supplementary material for: Rossby wave-modulated orbital precipitation anomalies in the Asia-Pacific region
Source: Nat Commun. 2026 Jun 16;17:7590. doi: 10.1038/s41467-026-74368-3 (PMC13421478; doi:10.1038/s41467-026-74368-3)
Supplement: Supplementary file 1 — Supplementary Information [file 41467_2026_74368_MOESM1_ESM.pdf]

Supplementary Material for

Rossby wave-modulated orbital precipitation anomalies in the Asia-Pacific region

Zhaojie Yu et al

This PDF file includes:
Figure S1 to S19
Table S1
References

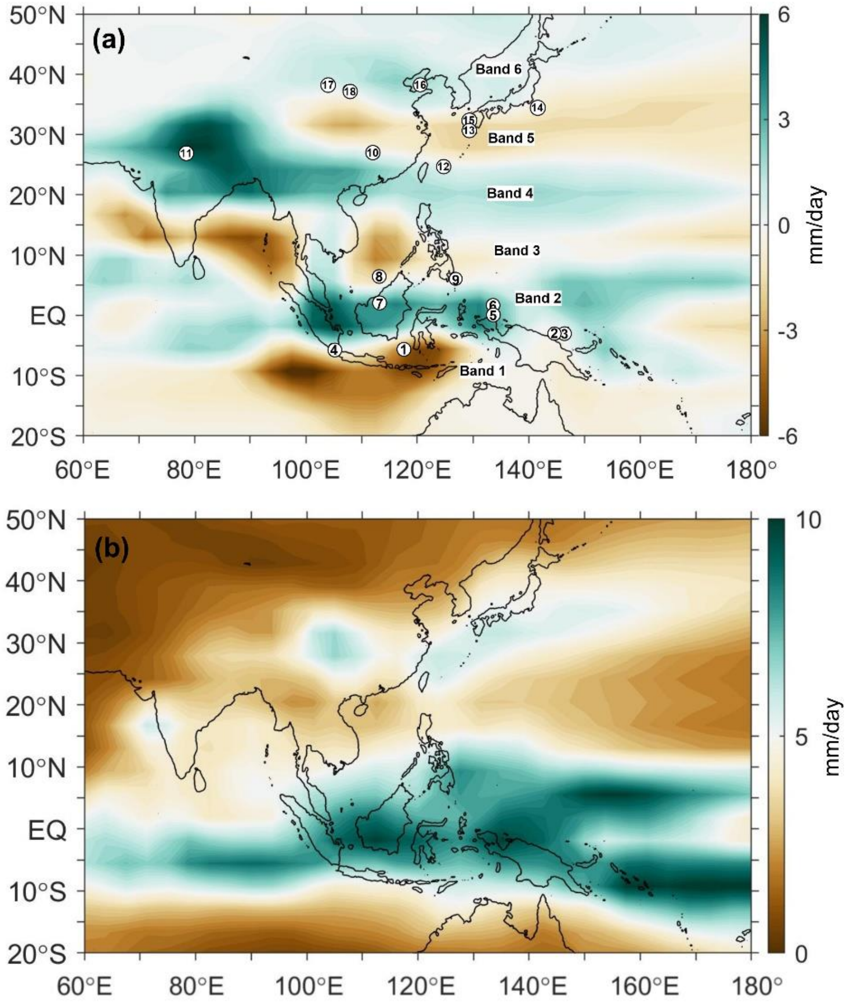

Figure S1 (a) Average summer (JJA) precipitation anomalies (mm/day) corresponding to the minimum minus maximum precession since 800 ka simulated by CESM 1.2 in the Asia-Pacific region. Band 1-6 indicate the hexapole banding areas. Circles and numbers represent the positions of the compiled precipitation records in Table S1 and Figures S2 and S3. (b) shows the mean precipitation (mm/day) distribution over 800 ka. Note that the color bars in (a) and (b) differ. Their comparison suggests that the magnitude of summer precipitation anomalies in the extratropics is close to the mean precipitation.

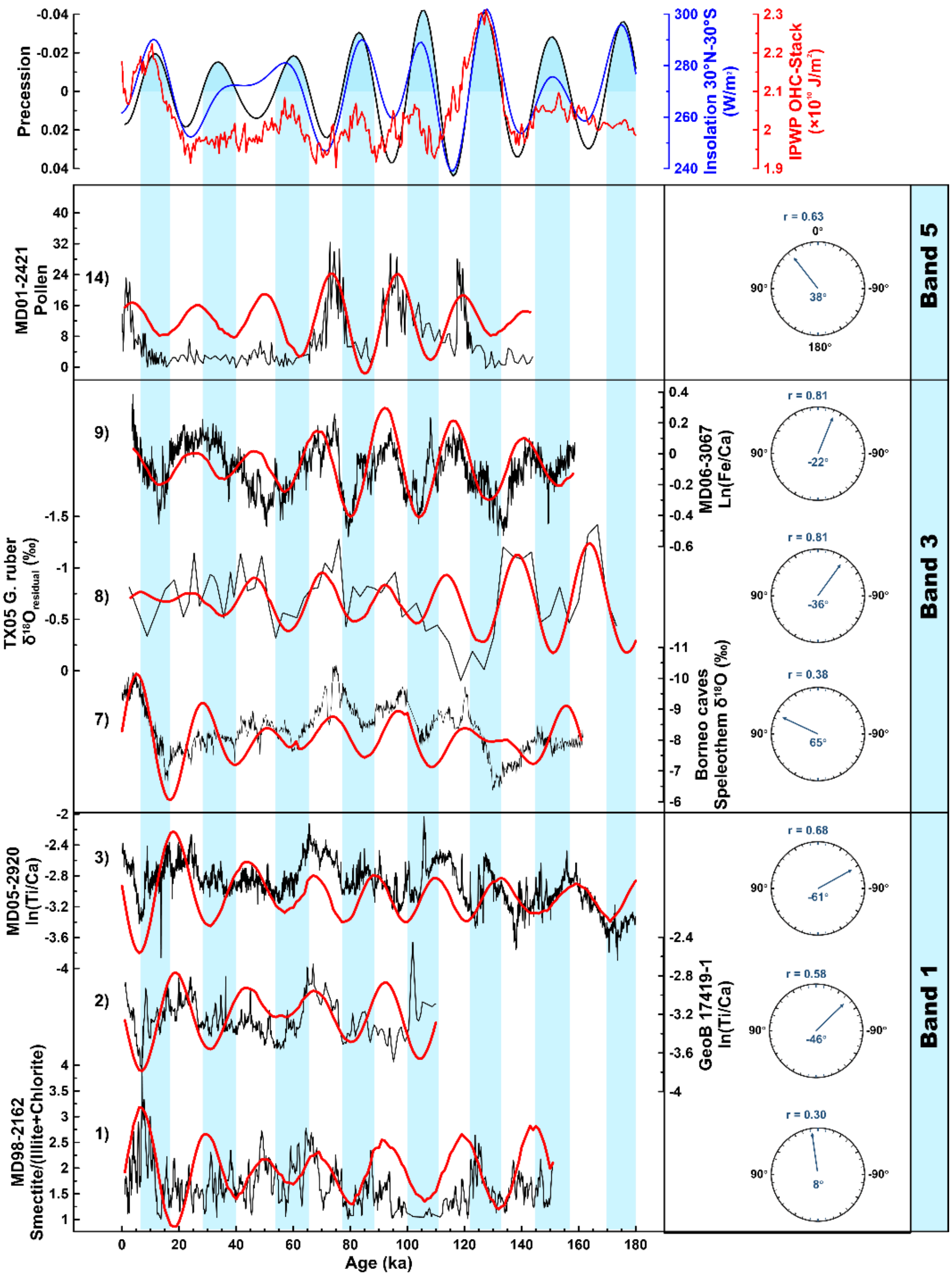

Figure S2 Comparisons of precession-paced precipitation changes for Band 1, 3 and 5. The detailed information of precipitation records is shown in Table S1. The red curves on top of each record are filtering curve of dominated precession cycle. The degrees and r values noted on the phase wheel indicate the phase angle and correlation coefficient between the precession and each record during the 23-kyr cycle. The upward direction of y-axis indicates more precipitation for all records. Note the precession axis is inverted and the maximum precipitation correspond to the maximum precession.

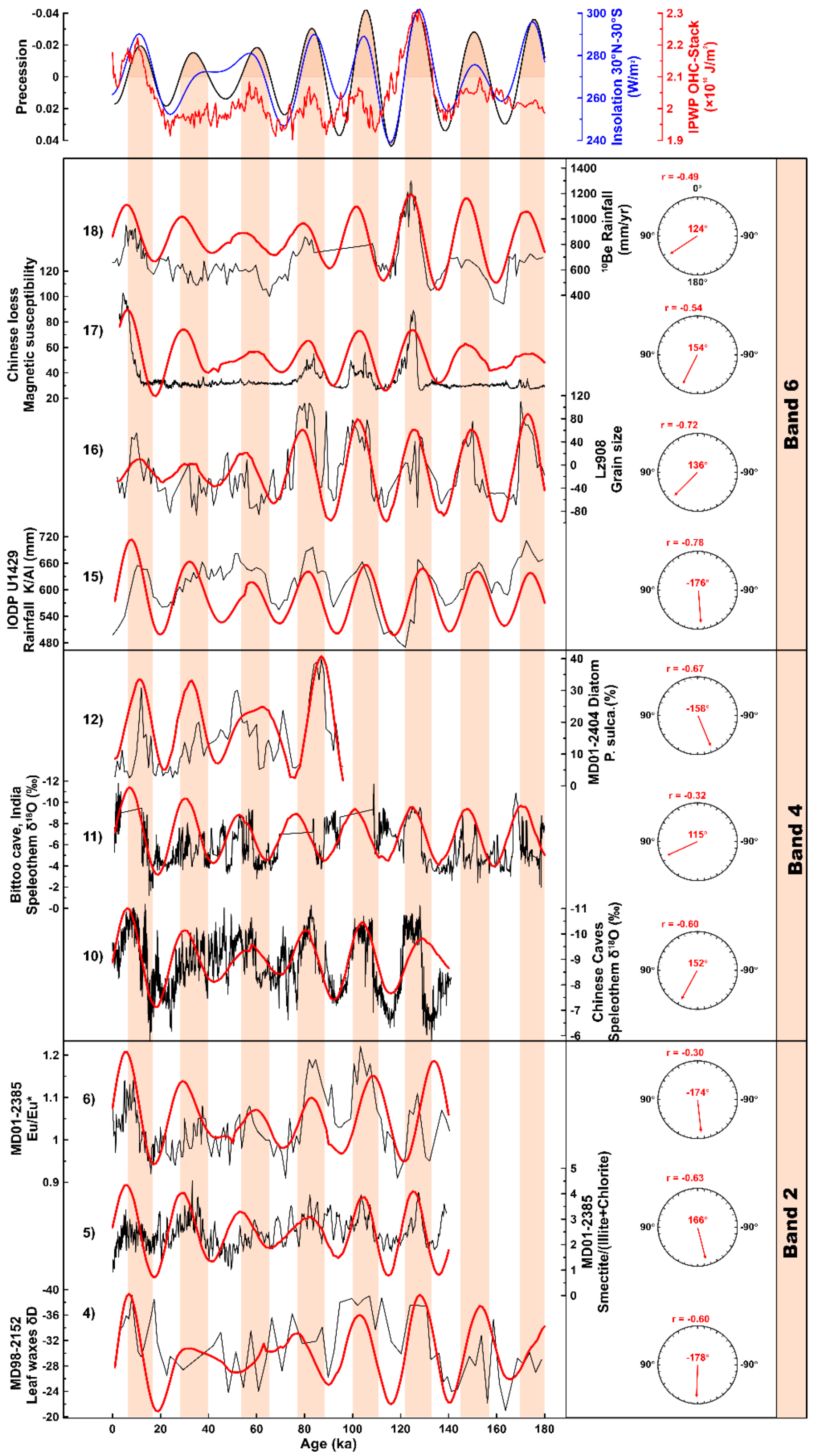

Figure S3 The same as Figure S2 but for Band 2, 4 and 6. Note the precession axis is inverted and the maximum precipitation corresponds to the minimum precession.

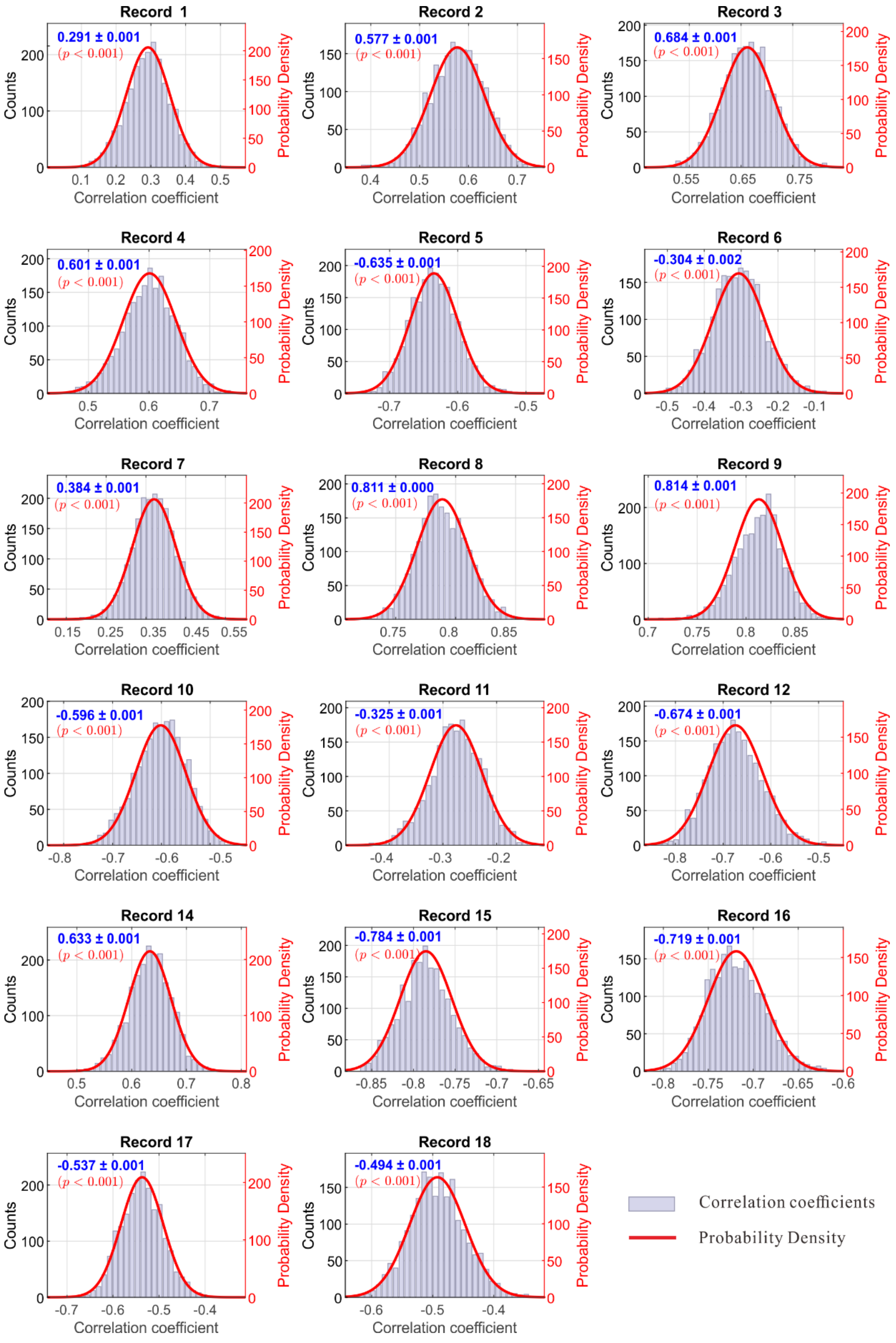

Figure S4 Histograms of correlation coefficients between filtering curve for the controlling cycle of each compiled precipitation record and precession. The correlation coefficients are calculated by linear interpolation of each curve to a resolution of 1-kyr. The Gaussian distribution (red lines) is used to fit each expected probability density curve. The correlation coefficients and their standard errors are also labeled.

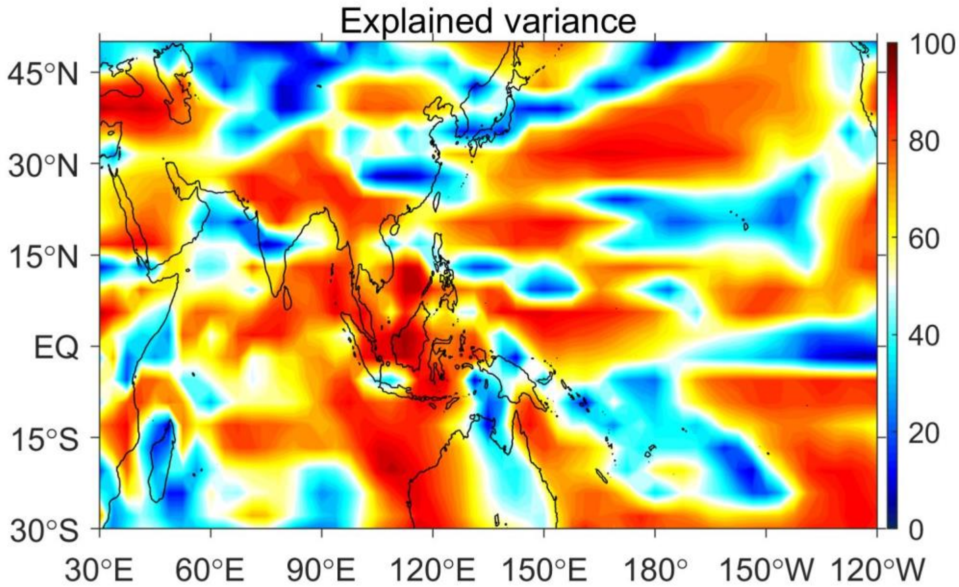

Figure S5 Explained variance (unit: %) of the simulated 23-kyr summer (JJA) precipitation anomalies relative to the total precipitation anomalies.

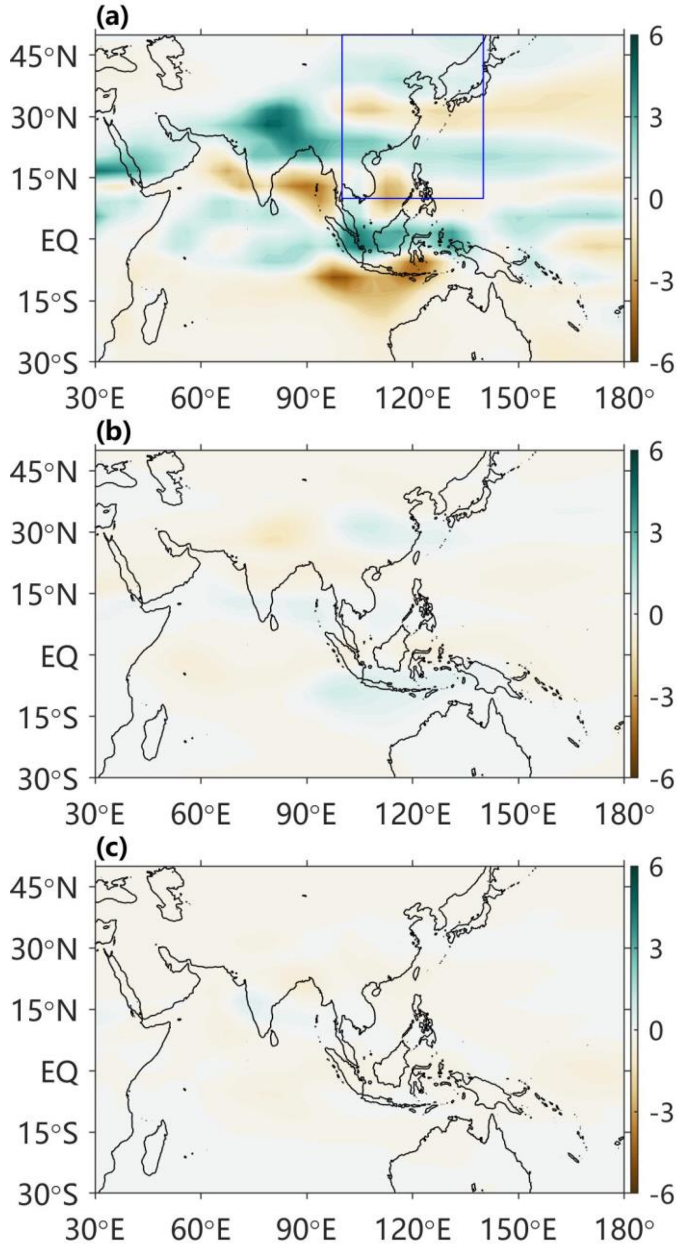

Figure S6 Simulated summer (JJA) precipitation anomalies in the Asia-Pacific region. Precipitation anomalies (mm/day) with Lanczos bandpass filtering [1] of (a) 17-28 kyr for precession, (b) 37-45 kyr for obliquity, and (c) 96-104 kyr for eccentricity, respectively. The summer precipitation anomaly is calculated based on the difference of minima and maxima of each orbital parameter.

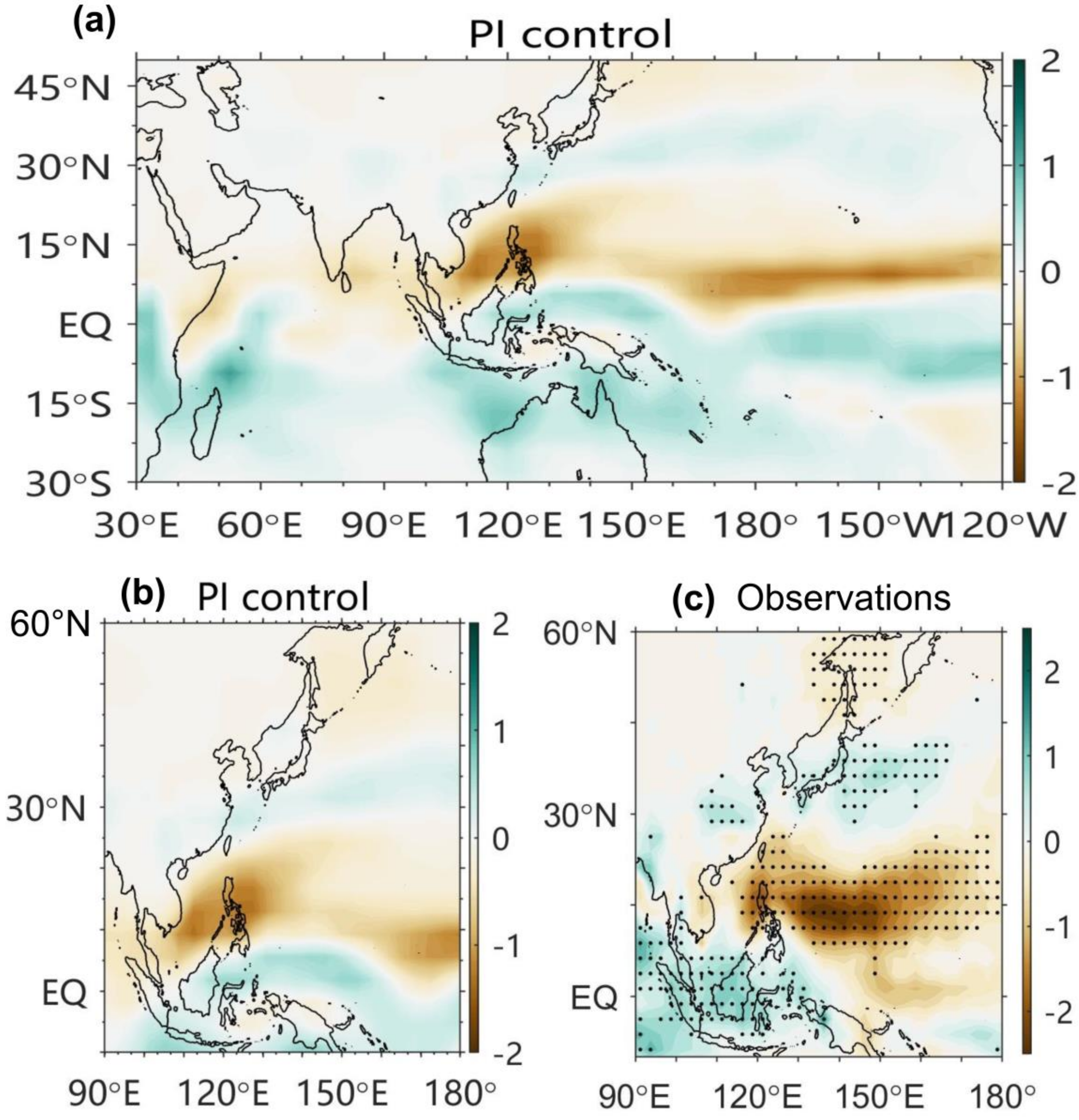

Figure S7 Structure of the Pacific-Japan (PJ) pattern. (a-b) Regressed summer precipitation anomalies (mm/day) using the identical methodology as in Figure 3c, based on the last 100-yr outputs derived from a preindustrial control simulation of the same CESM model. The simulation is configured with fixed CO_2 concentration at 285 ppm, present-day orbital parameters and geographic configuration, but with the land-sea mask fixed at the Last Glacial Maximum (LGM) boundary. (b) shows the same pattern as (a) but focus on the core domain of the PJ pattern. (c) Regressed summer precipitation anomaly for 1979-2014 based on CMAP observational data [2,3].

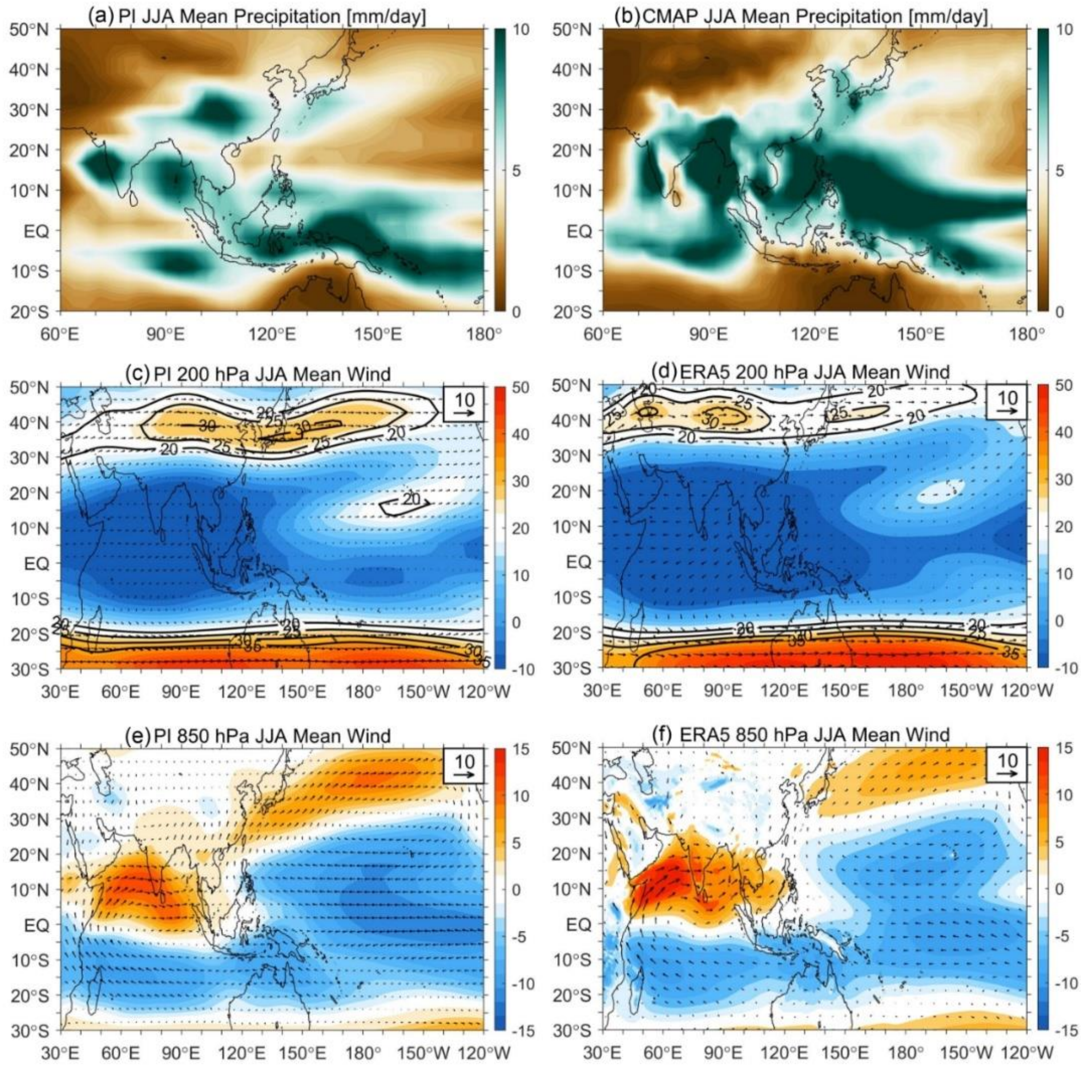

Figure S8 Validation of monsoon precipitation and circulation in the PI control simulation. JJA mean precipitation (units: mm/day) in the (a) PI control simulation and (b) CMAP observations. (c) JJA mean horizontal winds at 200 hPa in the PI control (shadings: zonal current; units: m/s; contours denote zonal wind with intervals of 5 m/s) and (d) ERA5 reanalysis. (e-f) are the same as (c-d) but at 850 hPa.

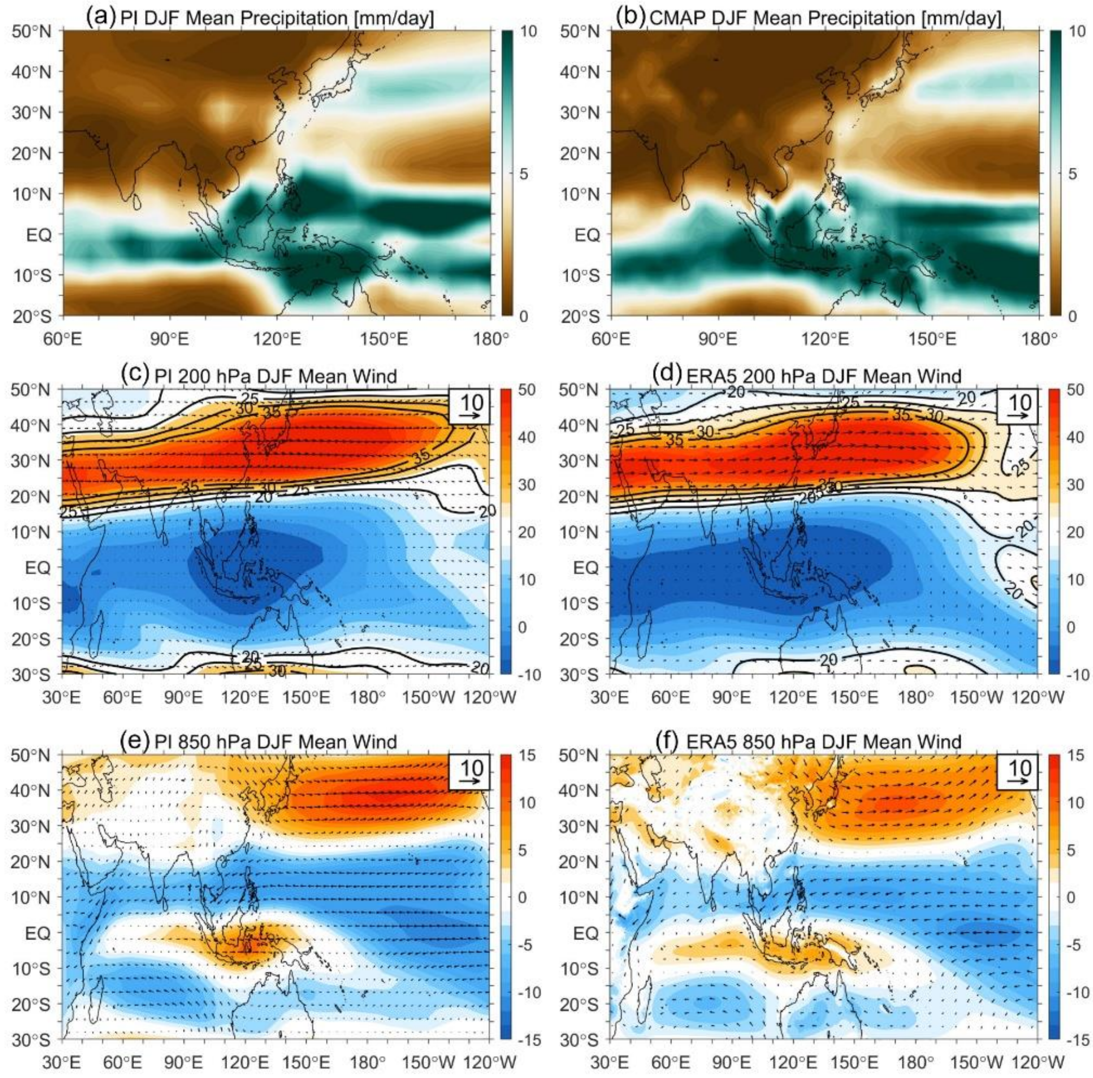

Figure S9 The same as Figure S8, but for winter.

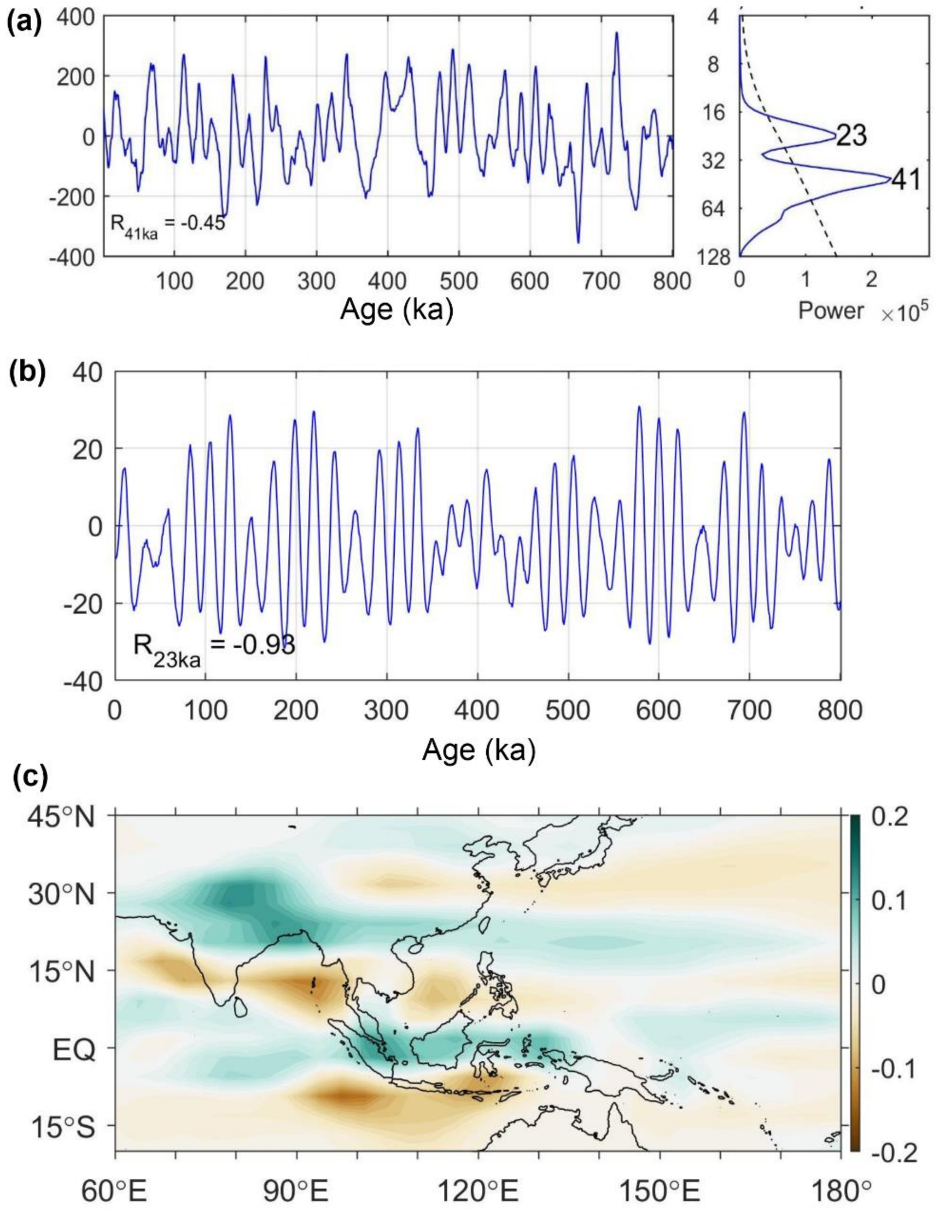

Figure S10 (a) As Figure 3a but for EOF2. The variance explained by EOF2 is relatively low at only 12.2%. (b-c) The leading mode of EOF analysis on simulated precipitation anomalies. The explained variance is 55.4%.

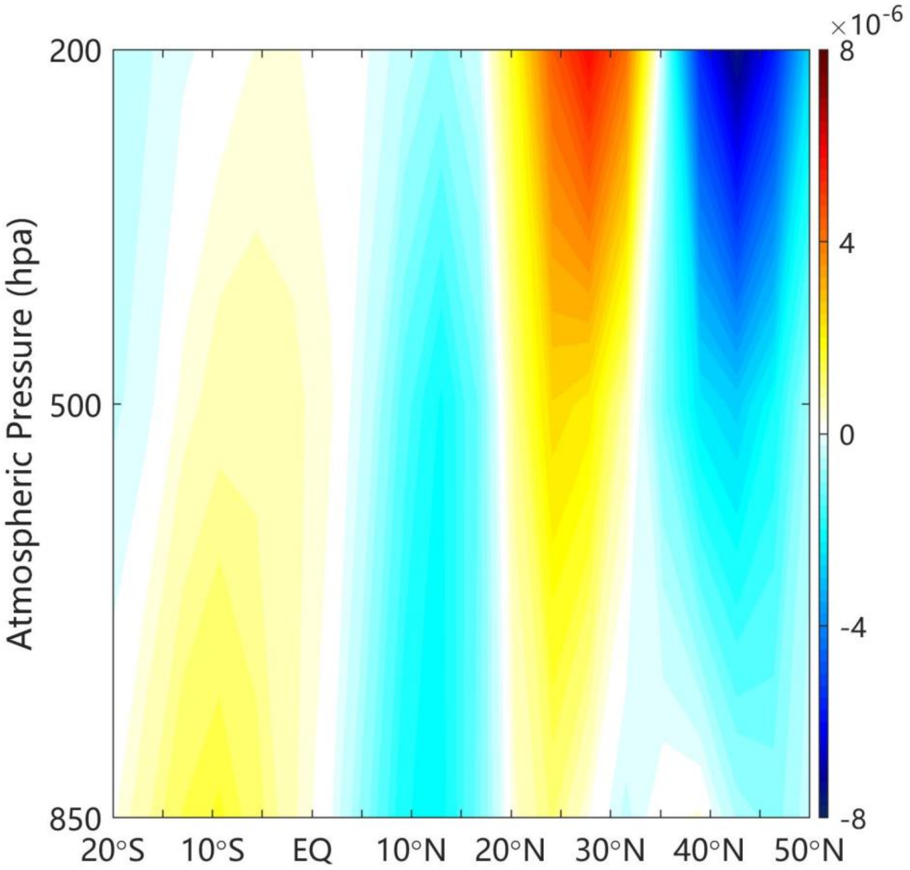

Figure S11 Latitude-height composite of the regressed relative vorticity along the primary Rossby wave propagation corridor (110°-120°E). A pronounced vertical gradient is evident within 5°S-5°N, indicative of baroclinic influence. In contrast, poleward of 5°N, the weakened vertical gradient suggests the dominance of barotropic effects.

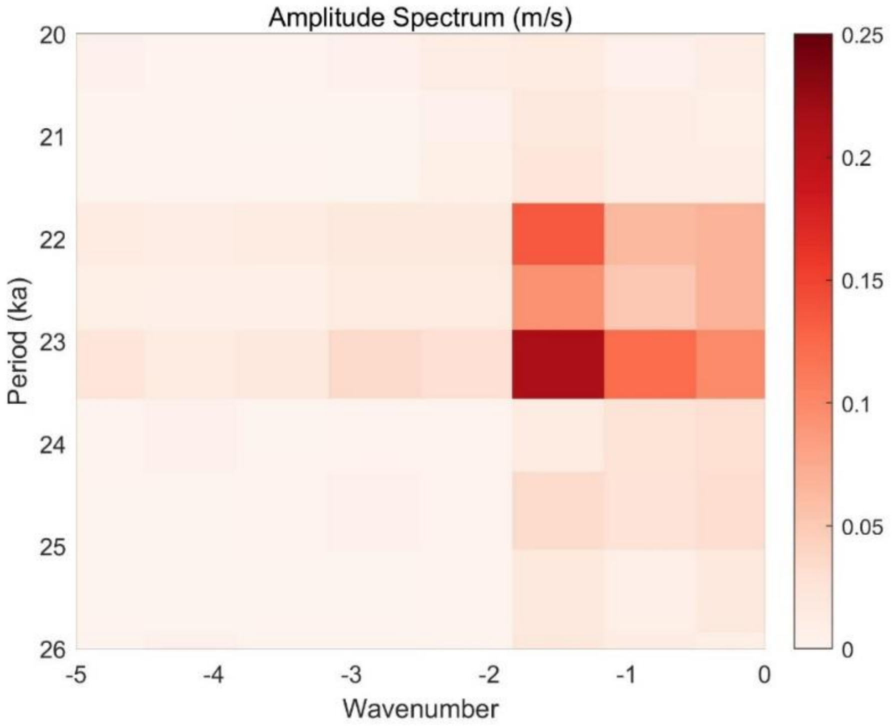

Figure S12 Rossby wave number-period spectral energy analysis of the 200 hPa JJA meridional wind anomaly (*v*') over 100°-120°E, EQ-60°N since 800 ka, calculated using a two-dimensional fast Fourier transform (FFT). The spectrum highlights dominant large-scale meridional variability on orbital timescales. Given the regional and zonally inhomogeneous nature of the circulation anomalies, these features are interpreted as low-order meridional wave structures rather than a single well-defined harmonic mode, consistent with the RWS–WAF diagnostics in the main text.

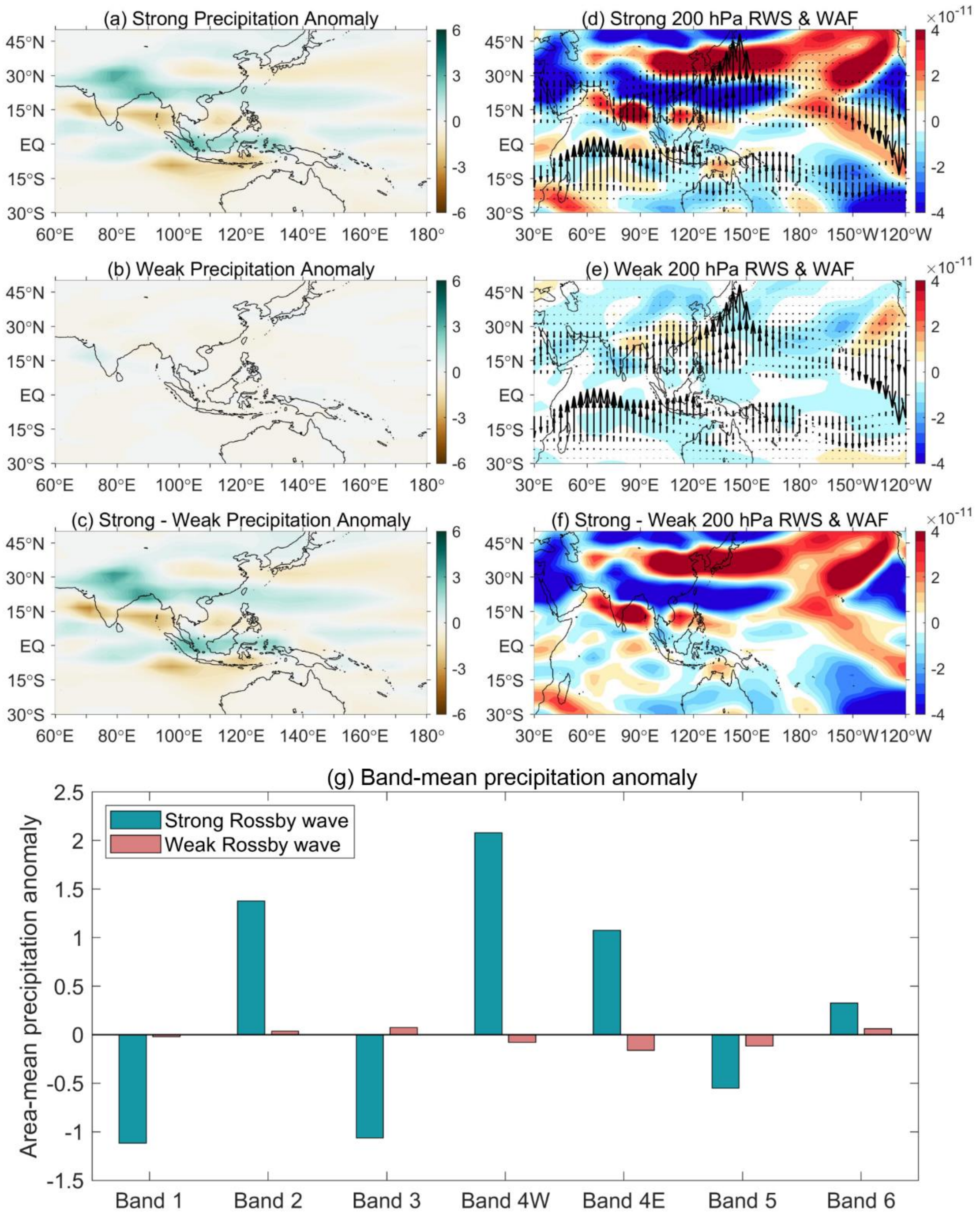

Figure S13 Rossby-wave modulation of banded precipitation. Precipitation anomaly during (a) strong Rossby-wave phases, (b) weak Rossby-wave phases and (c) difference between strong and weak phases (strong minus weak), respectively. The same as (a-c) but for Rossby wave source (RWS; shading) and wave activity flux (WAF; vectors) at 200 hPa. (g) Area-mean precipitation anomalies averaged over the predefined bands (Bands 1–6) for strong (green) and weak (red) Rossby-wave phases.

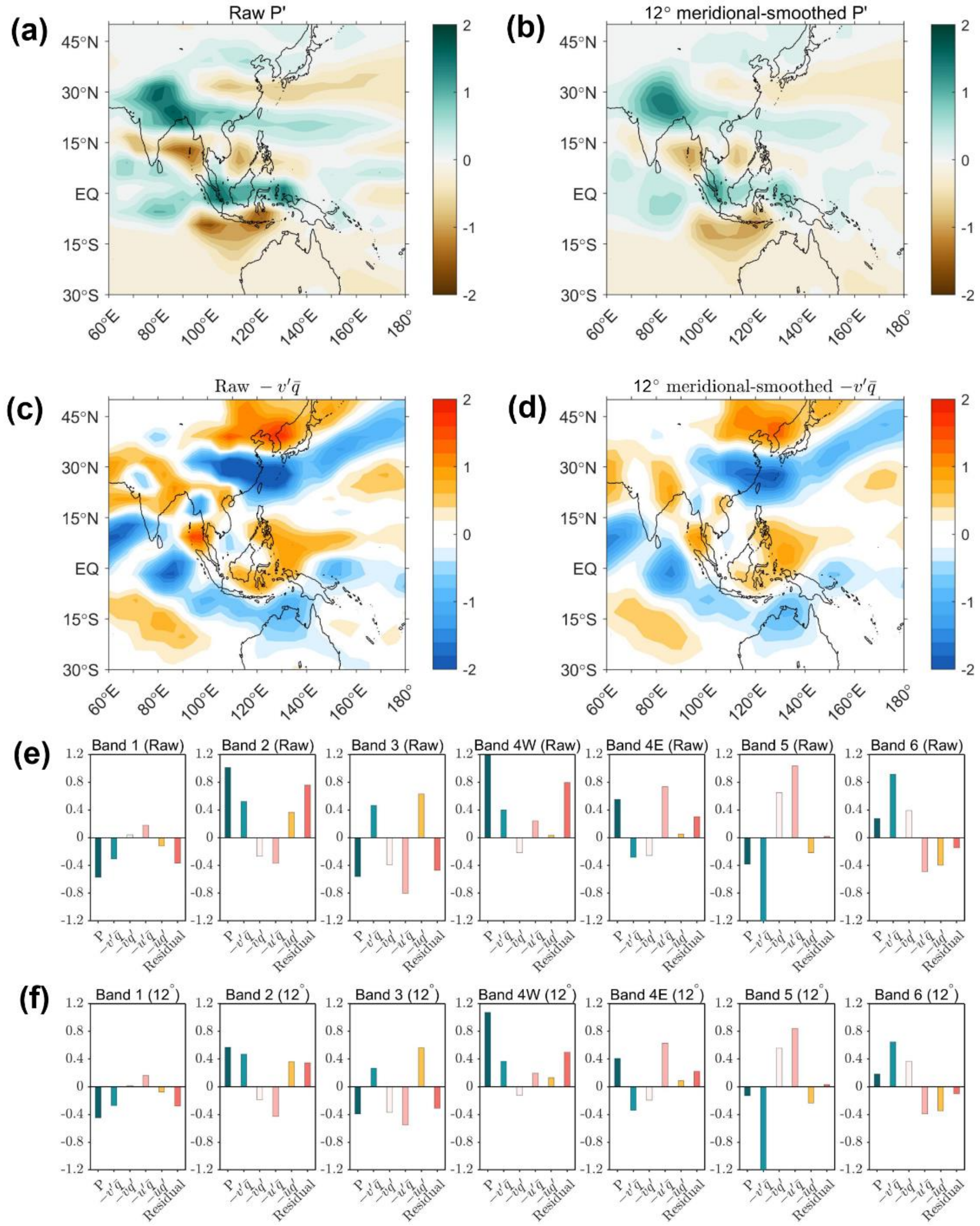

Figure S14 Robustness of banded precipitation structure and meridional moisture advection (- $v'\overline{q}$) under meridional smoothing. Spatial patterns of regressed precipitation anomalies (top) and - $v'\overline{q}$ associated with Rossby wave-mediated circulation and poleward moisture transport for (left) the original fields and (right) fields after applying a 12° meridional smoothing. Area-mean precipitation anomalies (P) and corresponding moisture budget terms for each band, for the original (raw) fields and fields after applying a 12° meridional smoothing.

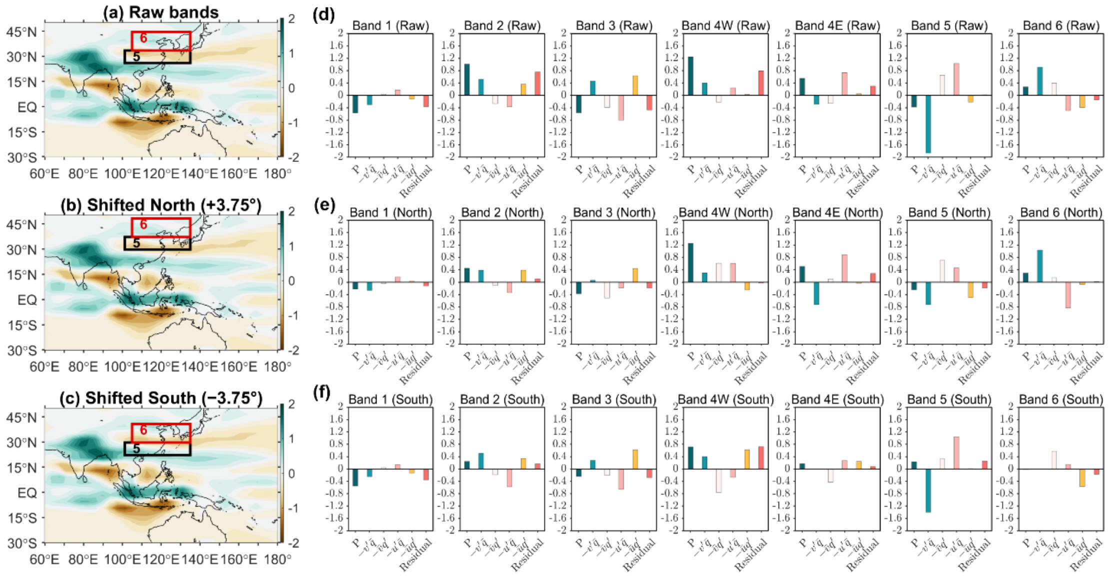

Figure S15 Sensitivity of moisture budget decomposition to meridional shifts of band boundaries. Regressed precipitation anomalies with diagnostic boxes for the (a) original bands, and after shifting all bands northward (+3.75°; b) and southward (−3.75°; c), corresponding to one grid spacing. Labels denote Bands 5 and 6, whose area-mean are sensitive to southward shifts because of incorporating opposite-signed anomalies from adjacent regions. (d–f) Area-mean precipitation (P) and individual moisture budget terms for each band under the original, north-shifted, and south-shifted configurations, respectively.

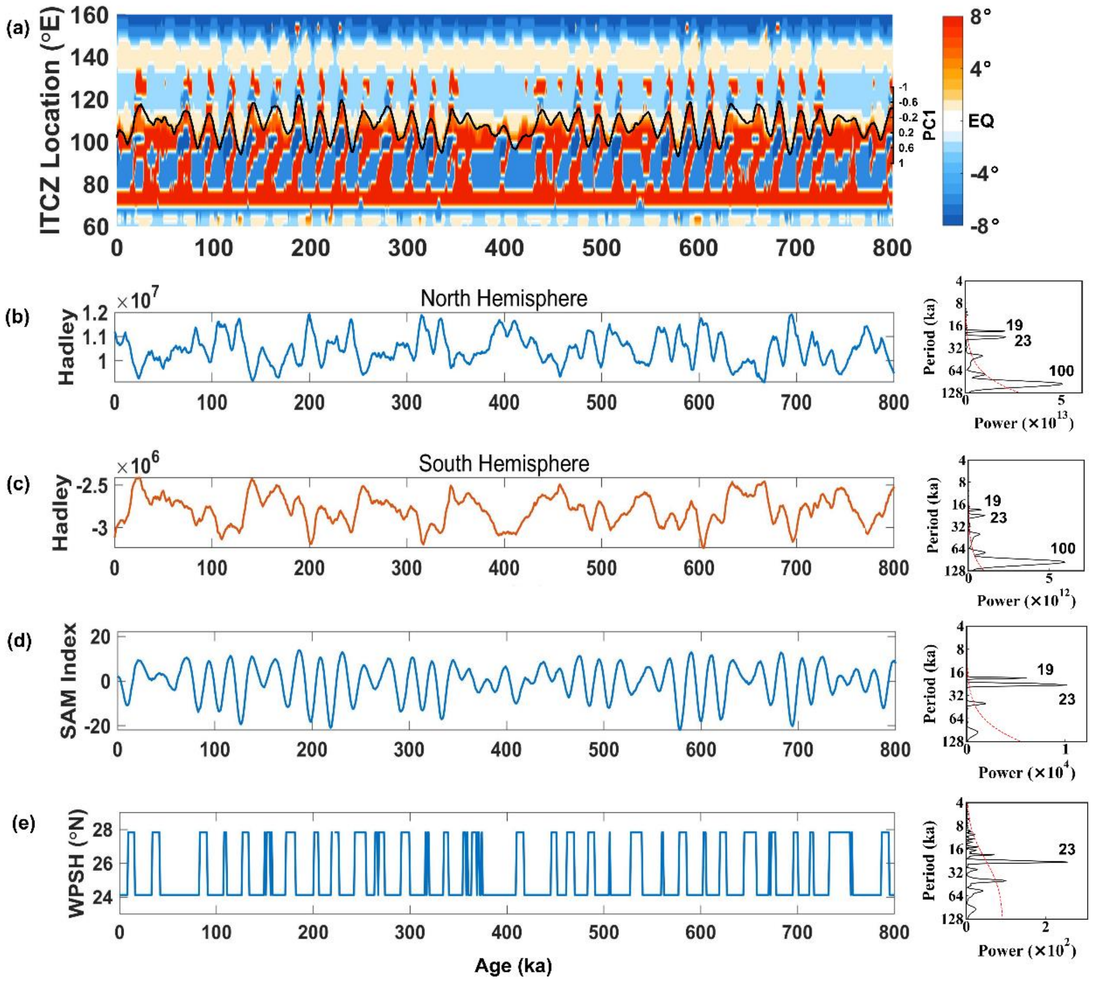

Figure S16 Relationships between summer (JJA) precipitation anomalies and potential atmospheric drivers from CESM1.2 simulations since 800 ka. (a) Latitudinal position of the ITCZ (unit: °); the black line denotes the time series of PC1. Intensities of the Hadley circulations in the (b) Northern Hemisphere and (c) Southern Hemisphere, respectively. (d) South Asian monsoon index. (e) Latitudinal extent (°) of the Western Pacific Subtropical High (WPSH). The right-hand panels present the periodicity analysis of the corresponding indices. Detailed definitions of these indices are provided in the Methods section.

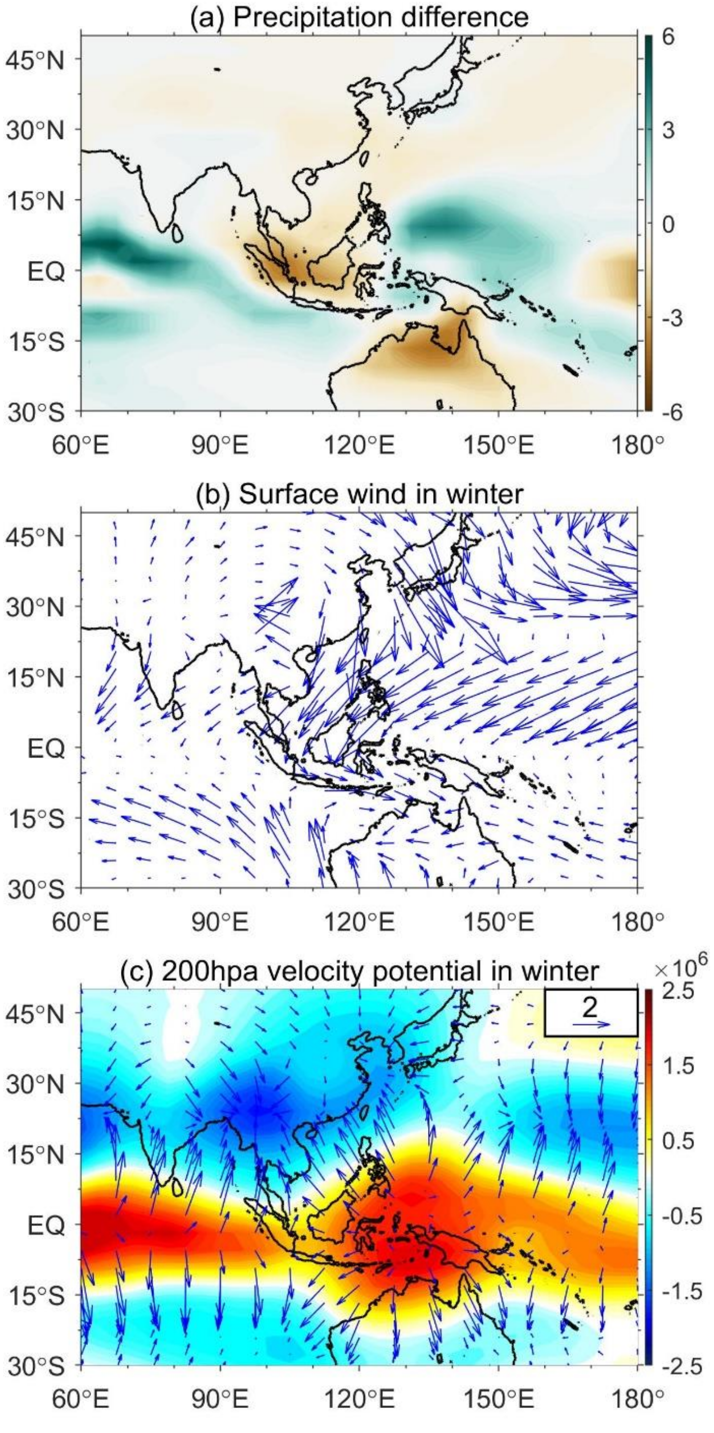

Figure S17 CESM simulation of precipitation and winds in winter (DJF). (a) The same as Figure 1 but for winter. (b) Surface wind stress (N m^{-2}) during winter. (c) 200-hPa velocity potential (shading; unit: 10^6 m^2 s^{-1}) and divergent wind (vectors). Positive velocity potential along the equator in winter are associated with strong precipitation, accompanied by the upper-level divergence. In contrast, the region of negative velocity potential between 15°N and 30°N reflects upper-level convergence.

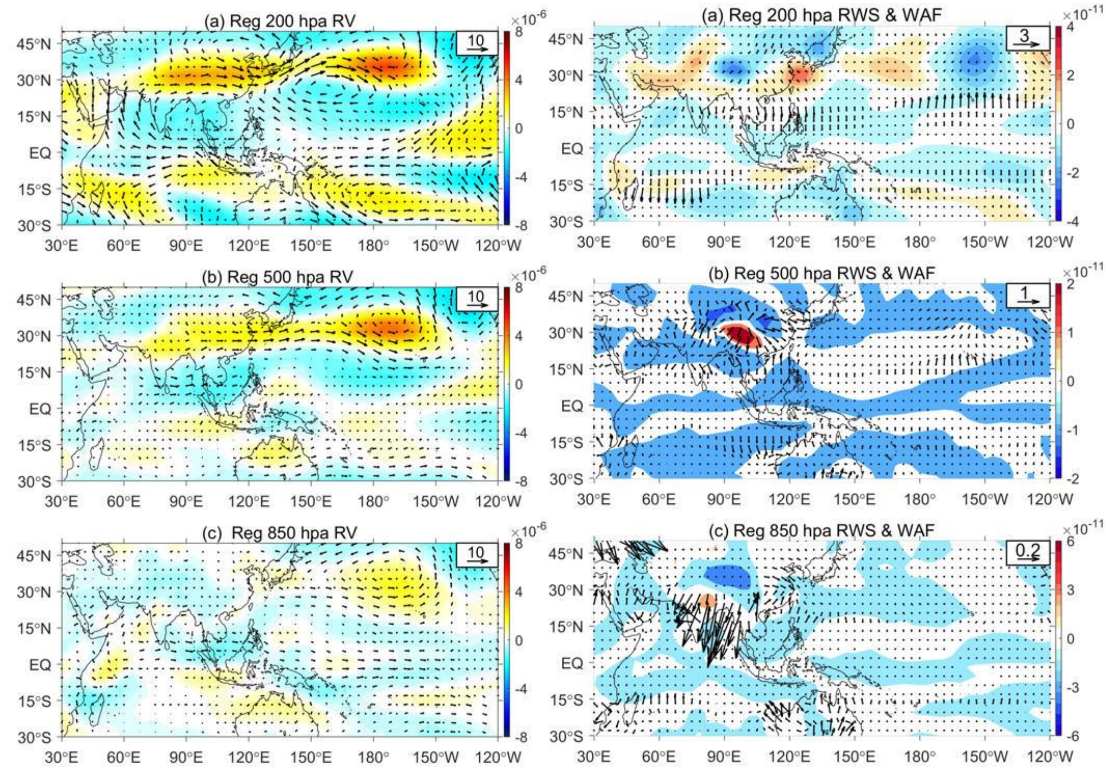

Figure S18 The same as Figure 4 but for winter (DJF).

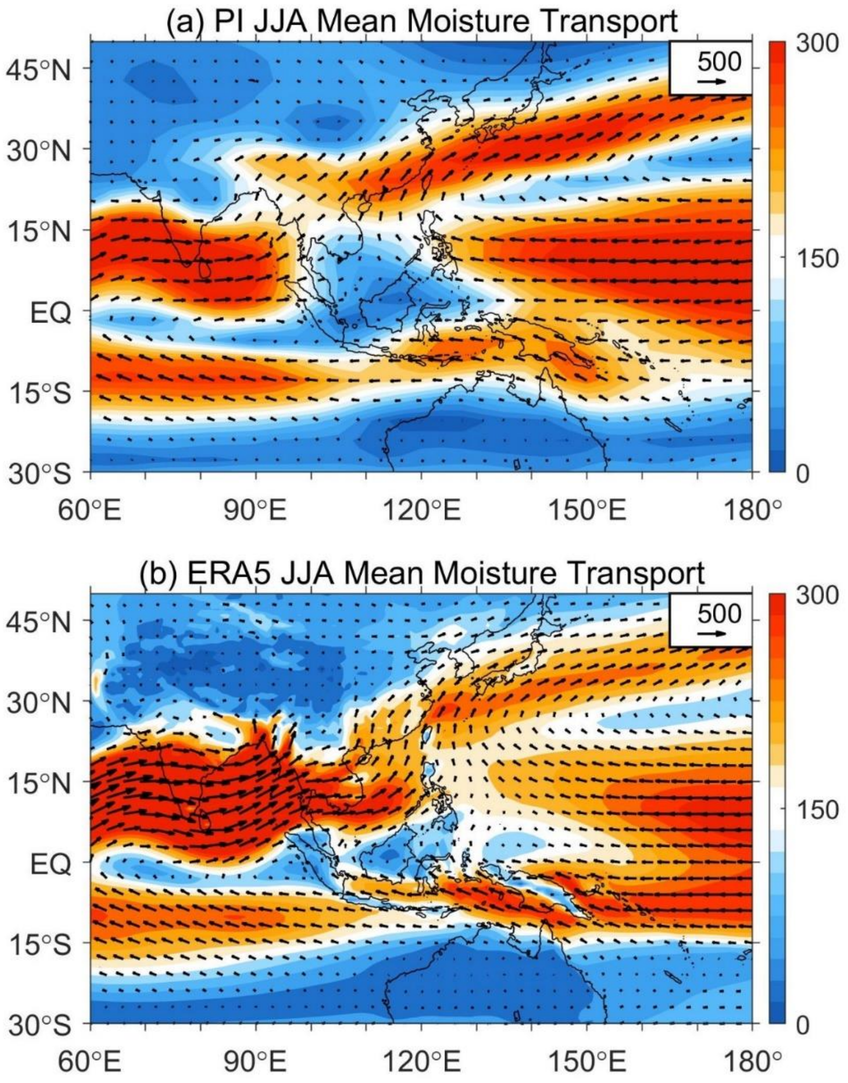

Figure S19 Validation of monsoon moisture transport in the PI control simulation. JJA mean vertically integrated moisture transport (vectors) and its magnitude (shadings; units: kg/m/s) in the (a) PI control and (b) ERA5.

Table S1 List of the climate archives from the Asia-Pacific region considered in this study. Note that "-" in the correlation with precession (r) represent anti-phase with precession maximum.

No.	Band	Name	Long. & lat.	Rainfall Proxies	Period (ka)	Correlation with precession (r)	References
1	1	MD98-2162	4.68°S 117.90°E	Smectite/(illite+chlorite)	0-150	0.30	This study
2		GeoB 17419-1	2.81°S 144.50 °E	Ti/Ca (XRF)	0-110	0.58	[4]
3		MD05-2920	2.85°S 144.53°E	Ti/Ca (XRF)	0-400	0.68	[5]
4	2	MD98-2152	6.18°S 106.83°E	δD of leaf waxes	0-450	-0.60	[6]
5		MD01-2385	0.22°S 134.24°E	Smectite/(illite+chlorite)	0-140	-0.63	[7]
6		MD01-2385	0.22°S 134.24°E	Eu/Eu*	0-140	-0.30	[7]
7	3	Borneo caves	4.10°N 114.88°E	$\delta^{18}O$	0-160	0.38	[8,9]
8		TX05	6.33°N 111.27°E	$\delta^{18}O_{sw}$	0-280	0.81	[10]
9		MD06-3067	6.50°N 126.50°E	Fe/Ca (XRF)	0-160	0.81	[11]
10	4	Sanbao Cave, China	31.67° N 110.86° E	$\delta^{18}O$	0-640	-0.60	[12]
11		Bittoo cave, India	30.77°N 77.76°E	$\delta^{18}O$	0-280	-0.32	[13]
12		MD01-2404	26.65°N 125.81°E	Diatom assemblage	0-100	-0.67	[14]
13	5	IODP U1429	31.62°N 129°E	$\delta^{18}O_{sw}$	0-400	No precession	[15]
14		MD01-2421	36.03°N 141.78°E	Pollen	0-150	0.63	[16]
15	6	IODP U1429	31.62°N 129°E	K/Al	0-400	-0.78	[17]
16		Lz908	37.15°N 118.97°E	Grain size	0-260	-0.72	[18]
17		Chinese loess	36.35°N 104.60°E	Magnetic susceptibility	0-750	-0.54	[19]
18		Chinese loess	34.43°N 107.12°E	^{10}Be	0-550	-0.49	[20]

References

1 Duchon, C. E. Lanczos filtering in one and two dimensions. *Journal of Applied Meteorology (1962-1982)*, 1016–1022 (1979).

2 Xie, P. & Arkin, P. Monitoring large-scale precipitation over the globe. (1997).

3 Xie, S.-P. *et al.* Indo-western Pacific ocean capacitor and coherent climate anomalies in post-ENSO summer: A review. *Advances in Atmospheric Sciences* **33**, 411–432 (2016). https://doi.org/10.1007/s00376-015-5192-6

4 Hollstein, M. *et al.* Variations in Western Pacific Warm Pool surface and thermocline conditions over the past 110,000 years: Forcing mechanisms and implications for the glacial Walker circulation. *Quaternary Science Reviews* **201**, 429–445 (2018).

5 Tachikawa, K. *et al.* The precession phase of hydrological variability in the Western Pacific Warm Pool during the past 400 ka. *Quaternary Science Reviews* **30**, 3716–3727 (2011). https://doi.org/https://doi.org/10.1016/j.quascirev.2011.09.016

6 Windler, G., Tierney, J. E., Zhu, J. & Poulsen, C. J. Unraveling Glacial Hydroclimate in the Indo-Pacific Warm Pool: Perspectives From Water Isotopes. *Paleoceanography and Paleoclimatology* **35**, e2020PA003985 (2020).

7 Yu, Z. *et al.* Late Pleistocene island weathering and precipitation in the Western Pacific Warm Pool. *npj Climate and Atmospheric Science* **7**, 91 (2024).

8 Carolin, S. A. *et al.* Northern Borneo stalagmite records reveal West Pacific hydroclimate across MIS 5 and 6. *Earth and Planetary Science Letters* **439**, 182–193 (2016). https://doi.org/https://doi.org/10.1016/j.epsl.2016.01.028

9 Carolin, S. A. *et al.* Varied response of western Pacific hydrology to climate forcings over the last glacial period. *Science* **340**, 1564–1566 (2013).

10 Yang, C., Xu, J. & Zhang, P. Glacial sea level low-stands regulated the upper ocean hydrology in the southern South China Sea over the past~ 280 kyr. *Palaeogeography, Palaeoclimatology, Palaeoecology* **629**, 111806 (2023).

11 Kissel, C. *et al.* Monsoon variability and deep oceanic circulation in the western equatorial Pacific over the last climatic cycle: Insights from sedimentary magnetic properties and sortable silt. *Paleoceanography* **25** (2010).

12 Cheng, H. *et al.* The Asian monsoon over the past 640,000 years and ice age terminations. *Nature* **534**, 640–646 (2016).

13 Kathayat, G. *et al.* Indian monsoon variability on millennial-orbital timescales. *Scientific Reports* **6**, 24374 (2016). https://doi.org/10.1038/srep24374
https://www.nature.com/articles/srep24374#supplementary-information

14 Chang, Y.-P., Wang, W.-L. & Chen, M.-T. The last 100 000 years' palaeoenvironmental changes inferred from the diatom assemblages of core MD012404 from the Okinawa Trough, East China Sea. *Journal of Quaternary Science* **24**, 890–901 (2009). https://doi.org/https://doi.org/10.1002/jqs.1316

15 Clemens, S. C. *et al.* Precession-band variance missing from East Asian monsoon runoff. *Nature Communications* **9**, 3364 (2018). https://doi.org/10.1038/s41467-018-05814-0

16 Yamamoto, M., Ichikawa, Y., Igarashi, Y. & Oba, T. Late Quaternary variation of lignin composition in core MD01-2421 off central Japan, NW Pacific. *Palaeogeography, Palaeoclimatology, Palaeoecology* **229**, 179–186 (2005). https://doi.org/https://doi.org/10.1016/j.palaeo.2005.06.021

17 Zhao, D. *et al.* Quaternary rainfall variability is governed by insolation in northern China and ice-sheet forcing in the South. *Communications Earth & Environment* **4**, 7 (2023). https://doi.org/10.1038/s43247-022-00670-9

18 Yi, L. *et al.* Late Quaternary linkage of sedimentary records to three astronomical rhythms and the Asian monsoon, inferred from a coastal borehole in the south Bohai Sea, China. *Palaeogeography, Palaeoclimatology, Palaeoecology* **329-330**, 101–117 (2012). https://doi.org/https://doi.org/10.1016/j.palaeo.2012.02.020

19 Sun, Y. *et al.* East Asian monsoon variability over the last seven glacial cycles recorded by a loess sequence from the northwestern Chinese Loess Plateau. *Geochemistry, Geophysics, Geosystems* **7** (2006). https://doi.org/https://doi.org/10.1029/2006GC001287

20 Beck, J. W. *et al.* A 550,000-year record of East Asian monsoon rainfall from 10Be in loess. *science* **360**, 877–881 (2018).
