# Supplementary material for: Rossby wave-modulated orbital precipitation anomalies in the Asia-Pacific region
Source: Nat Commun. 2026 Jun 16;17:7590. doi: 10.1038/s41467-026-74368-3 (PMC13421478; doi:10.1038/s41467-026-74368-3)
Supplement: Supplementary file 2 — Description of Additional Supplementary Files [file 41467_2026_74368_MOESM2_ESM.pdf]

File Name: Supplementary Data 1
Description: Clay mineral data of MD98-2162

File Name: Supplementary Data 2
Description: The original and filtered data presented in Table S1
