# Supplementary material for: Rossby wave-modulated orbital precipitation anomalies in the Asia-Pacific region
Source: Nat Commun. 2026 Jun 16;17:7590. doi: 10.1038/s41467-026-74368-3 (PMC13421478; doi:10.1038/s41467-026-74368-3)
Supplement: Supplementary file 5 — Transparent Peer Review file [file 41467_2026_74368_MOESM5_ESM.pdf]

nature portfolio

Peer Review File

Rossby wave-modulated orbital precipitation anomalies in the Asia-Pacific region

Corresponding Author: Dr ZHAOJIE Yu

This file contains all reviewer reports in order by version, followed by all author rebuttals in order by version.

Attachments originally included by the reviewers as part of their assessment can be found at the end of this file.

Version 0:

Reviewer comments:

Reviewer #1

(Remarks to the Author)
Detailed comments are attached as pdf file

My recommendation is major revisions

*What are the noteworthy results?
The noteworthy results of this paper are a climate model that demonstrates the potential role of Rossby waves in regulating Asian monsoon rainfall over orbital timescales. This is an overlooked/under-explored mechanism for explaining Quaternary paleo-climate change.

*Will the work be of significance to the field and related fields? How does it compare to the established literature? If the work is not original, please provide relevant references.
The work is original. It will be significant to paleo-climatology, modern climate change, geology, other geoscience fields, and perhaps environmental policy for anthropogenic contingencies.

*Does the work support the conclusions and claims, or is additional evidence needed?
The modeling work supports the hypothesis about Rossby waves. However, the research fails to exclude other hypotheses that explain the paleo-climate data. Therefore, instead of eliminating other possibilities, the research nominates an addition control on orbital patterns of Asian hydroclimate.

*Are there any flaws in the data analysis, interpretation and conclusions? Do these prohibit publication or require revision?
Revision is required. A main flaw is with the interpretation of the smectite data, which I provide detailed comments via the attached pdf. Also, the supposed latitude trend in phase relationships is just as easily explained by outlier data. There is not strong evidence, statistically or otherwise, for a latitudinal spatial trend in phase relationships between the paleo-climate records and the astronomical solution for precession/insolation.

Is the methodology sound? Does the work meet the expected standards in your field?
Yes

Is there enough detail provided in the methods for the work to be reproduced?
Yes

Reviewer #2

(Remarks to the Author)
Review on “Rossby wave–regulated orbital precipitation in the Asia–Pacific region” submitted to Nature Communications by Zhaojie Yu et al.
The study presented by Zhaojie Yu et al. investigates the relevance of orbital forcing for the distribution of precipitation in the Asia–Pacific region. The authors identify the precession cycle, operating on ~19–23-kyr timescales, as the most relevant

orbital component when compared to obliquity and eccentricity. The study combines existing sediment records (representing precipitation in different latitudinal bands) with one new record (MD98-2162), together with climate model simulations conducted with CESM. The authors argue that the observed precipitation signals, in both sediments and model results, are predominantly driven by the precession cycle.

The authors further identify a hexagonal banded structure in the region of interest, which includes the Pacific–Japan tripole pattern. They argue that the observed pattern is shaped by anomalies in the Indo-Pacific warm pool, which modulate convection and the subsequent propagation of meridional Rossby waves. They conclude that orbitally forced IPWP anomalies have an analogue in a warming world and that their results may help constrain uncertainties in the region by better defining the range of naturally induced variability.

In general, the paper is well written. Some figures require substantial revision, and the text could be significantly streamlined in places. However, I find the choice of meteorological diagnostics difficult to understand and, at times, somewhat arbitrary. In its current form, the conclusions are, in my view, not clearly enough articulated, not sufficiently supported by the presented evidence, and I struggle to follow the overall narrative. Furthermore, uncertainties and statistical testing are insufficiently addressed in the present version of the manuscript.

My major reservation concerns the degree of novelty of the study. There already exists a considerable body of work on orbitally forced precipitation anomalies over Asia, and it is not yet clear to me what the specific selling point of the present analyses is that would justify publication in a highest-impact journal. The authors need to clearly communicate their unique selling point compared to the existing literature that would justify publication in such a high-impact venue. If the issues outlined below can be addressed in a thorough major revision, this work might become suitable for consideration for publication in Nature Communications.

Major comments:

1. Sediment core analysis:
It is unclear to me what the inclusion of the new sediment core MD98-2162 adds to the overall findings. To my understanding, there are already plenty of sediment core analyses covering all relevant latitudinal bands. It is also not clear why some of the cores are included in Figure 1, while others are only shown in the Supplementary Material. This choice requires clearer motivation and justification, especially for readers who are not fully embedded in this specific field. It also needs to be discussed (even if only briefly) why the different sediment cores deviate so strongly in their lead–lag times, even though they are, from a global perspective, rather close to each other.

2. The model used in the context of learning from the past for the future:
The proposed mechanism is based on the assumption that (positive) anomalies in IPWP heat content modulate convection and meridional Rossby wave propagation, which, shaped by the natural topography, ultimately produces the observed regional precipitation anomalies. The model resolution used here (3.75° horizontal spacing and 26 vertical levels; please also define the model top), however, is rather coarse, and one could question the ability of the model to realistically capture regional precipitation regimes, especially if Rossby wave propagation and complex topography are essential parts of the overall mechanism. Therefore, before drawing far-reaching conclusions, it is, in my opinion, essential that the authors demonstrate that the model is capable of reproducing precipitation anomalies associated with the PJ pattern during the contemporary era. This could, for example, be achieved by phase composites or comparable diagnostics. Furthermore, if the authors wish to draw conclusions along the lines of "learning from the past for the future," they should show how the model responds to future increases in greenhouse gases in the context of the proposed mechanism. The necessary data should be available; if this is not the case for dedicated future simulations, one could at least compare the latest decades of climate change to a situation around the 1850s (typically represented by pre-industrial forcing). This would really provide a sense of how the results differ between past, contemporary, and future climate states within the same modelling framework.

3. Inconsistent/arbitrary analysis underlying Figures 2 and 3, variability range and statistics:
In my understanding, the overarching idea is that, if the orbitally forced precipitation component is projected onto the climatological mean state of the summer dynamics in the region of interest, the emergence of the hexagonal structure can be understood. However, the meteorological variables shown in Figures 2 and 3 differ from each other. This makes it very difficult to follow the narrative. For example, Figure 2 shows precipitation, 500-hPa geopotential height, 200- and 850-hPa relative vorticity, and 200-hPa wave activity flux, whereas Figure 3 shows wind vectors from the surface to 200 hPa and 200-hPa velocity potential. Both analyses need to show the same quantities; otherwise, it is difficult to understand how the orbitally driven component differs from the mean state and how both interact. Please include the variables shown in Figure 2 in Figure 3, and vice versa. It is also unclear why the authors chose these specific levels. At this stage, the analysis appears somewhat ad hoc; a more consistent and unified framework is necessary, and the rationale for the chosen diagnostics and levels should be clearly explained in the main text or, at minimum, in the Supplementary Information. In addition, all colour bars currently lack physical units; these should be included. Finally, Figures 1 and 2 show the signals associated with orbital forcing, but it is not clear how the magnitude of these signals (e.g. the precipitation response of ±2 mm day^{-1} in Figure 2c) compares to the overall variability of precipitation in this region. This needs to be shown to give the reader a sense of how large the contribution of orbital forcing actually is. Additionally, nowhere in the manuscript is it described how the significance tests are performed; it only states, for example, that results "pass the 95% confidence level." Which statistical test is used, and which software or toolbox is employed? Please provide this information.

Minor comments:
44: strike out “amounts”.
46: add reference for statement on decline in precipitation.
50–53: see comment on “learning from the past for the future”.
58: maybe change “moisture” to “wet conditions”.
60: briefly explain the reason for the spread in lags.
62: clarify what is meant by “insignificant lag”.
62–66: explain how this study contributes to addressing this lack of understanding; see major comment above.
67: change “integrate” to “combine”.
69: explain why this record is essential for the overall narrative; see major comment above.
71: change “extracted” to “analyze”.
72: change “precipitation records” to “precipitation time series”.
Figure 1: design is somewhat unfortunate; it would fill a complete page in print. Consider redistributing subfigures. In 1a, the text (sediment locations) is not readable. Why do you show min–max, given that your record (1) suggests the opposite (e.g. positive response in band 1)? Please rearrange so 1a better follows your narrative. Your filtering method is nowhere explained; add details in the Methods section.
It is interesting to see that during high-precession periods surface solar insolation in the tropics is reduced, which appears to shape your overall pattern. This resonates well with studies suggesting a similar pattern under reduced solar activity based on climate reconstructions and state-of-the-art chemistry–climate modelling (Rimbu et al., 2024). Please consider discussing these findings, as they would strengthen your argument that naturally driven variability can indeed modulate long-term precipitation in this region.*
95–98: here you explain the sediment record, but the composite maps show the exact opposite; please rearrange and clarify.
118–145: justification that precession is the dominant component of orbital forcing is unnecessarily long; condense to 2–3 sentences and refer to the Supplementary Material for methodological details.
218: why is this not shown? It would only add one figure to the Supplement and seems essential.
220: clarify what is meant by “limited layers” (or correct the term if misspelled).
Figure 2: same quantities should be shown consistently in Figures 2 and 3. The colour bar in 2d is incorrect; it should be symmetric (e.g. –40 to 40) with the colour transition between blue and red at zero, otherwise it is very difficult to interpret. In general (as in 2c), it is conventional to show positive precipitation in blue and negative in red; please adjust.
Figure 3: see comments for Figure 2 and major comments above.
238: consider replacing “combination” with “interaction”.
243: clarify what is meant by “annual wind field” (annual mean? climatology?).
274: relabel subsection/title as “Topographic effects”.
Figure 4: this figure can serve as an early version before submission but needs substantial revision. For example, the Rossby-wave-induced anomalies are currently shown over the open ocean, but according to the manuscript they should be located over the continent to depict topographic effects.
316–317: you refer to the known PJ pattern here but need to show that the model can realistically capture it; see major comment above.
References:
*Rimbu, N., Spiegl, T., Ionita, M., Doshi, S., & Lohmann, G. (2024). A synoptic scale perspective of solar forcing on extreme precipitation and floods over Europe during summer. Journal of Geophysical Research: Atmospheres, 129(21), e2024JD041952.

Reviewer #3

(Remarks to the Author)
This manuscript combines a new 150-kyr clay-mineral precipitation reconstruction from core MD98-2162 with an 800-kyr transient CESM1.2 simulation and proposes that orbital-scale hydroclimatic variability across the Asia–Pacific is regulated by meridionally propagating Rossby waves excited by precession-modulated IPWP convection. The authors identify a “hexapole” banding structure in modelled and reconstructed precipitation and interpret this as evidence for a planetary-wave–driven precipitation mechanism operating on orbital timescales.

The manuscript presents valuable new proxy data and an ambitious proxy–model comparison. However, the central physical mechanism — extension of synoptic-timescale Rossby wave dynamics to explain orbital-scale precipitation patterns, is not yet convincingly demonstrated. The evidence linking precession forcing to Rossby-wave energetics and propagation remains circumstantial, and several key diagnostics (moisture budget, stationary wave analysis, seasonal sensitivity, tests against alternative mechanisms) are missing. The manuscript therefore requires major revision before it can be considered for publication.

Major comments:

1. The central claim about meridionally propagating Rossby waves regulate precipitation on orbital timescales is not physically well-founded. Rossby waves are synoptic–seasonal atmospheric responses to instantaneous heating anomalies. Orbital forcing, by contrast, modulates seasonal mean states, not fast-timescale wave dynamics directly. The manuscript does not establish how a fast atmospheric mode can be treated as the primary cause of slow climate variability. Without a clear theoretical framework linking mean-state changes to stationary Rossby wave structures, the interpretation risks being circular.

2. Several well-established orbital-scale mechanisms may explain the latitudinal precipitation patterns: ITCZ migrations forced by hemispheric energy balance; land–sea thermal contrast and monsoon intensity changes; shifts in the westerly jet and Western Pacific Subtropical High; precession-controlled changes in tropical SST gradients and Walker circulation. These are briefly mentioned but not tested quantitatively. The current narrative assumes Rossby waves dominate without demonstrating that these alternative mechanisms are less significant.

3. The Methods section provides only a very brief description of the 800-kyr CESM1.2 transient simulation and refers readers to external publications for details. This level of description is inadequate for a study in which the model results constitute a major line of evidence supporting a new dynamical mechanism. Key external forcings, particularly ice-sheet geometry and topography, their source datasets, temporal resolution, and implementation strategy are not described.Also the treatment of CO_2 and other greenhouse gases, orbital forcing, coastline changes, and boundary condition interpolation is unclear. The manuscript also lacks essential information on model spin-up, drift behavior, and computational stability. As the model output forms a major component of the argument, some form of validation should be presented to demonstrate that the simulation realistically captures modern and orbital-scale monsoon dynamics and Rossby-wave patterns. Without transparent documentation of forcing fields, boundary-condition updates, and model performance, it is difficult to assess whether the simulated hexapole precipitation pattern reflects real climate dynamics or model-specific behavior. These issues must be addressed comprehensively in the Methods section.

4. The six-band precipitation structure appears visually constructed and could be: (1) an artifact of model resolution; (2) related to the monsoon shear line or WPSH position, (3) or a zonal-mean precipitation response to orbital-scale ITCZ shift. No objective clustering or eigenmode analysis is shown. Without quantitative detection, the band boundaries are subjective.

5. Many marine and terrestrial records used have tuning uncertainties of 2–5 kyr, comparable to the precession cycle phase. But Fig. 1b interprets sub-kiloyear lead/lag patterns across latitude bands. These phase relationships are likely not statistically meaningful.

6. Clay mineral ratios (smectite/illite+chlorite) depend on lithology, erosion intensity, transport path length, and vegetation cover, not solely on precipitation. The manuscript overstates the linearity of this proxy–climate relationship.

7. There is no breakdown of precipitation anomalies into dynamic moisture advection, thermodynamic moisture changes, vertical velocity anomalies, stationary eddy contributions. Without this, the driver of precipitation changes cannot be identified.

8. To claim a Rossby-wave mechanism, the manuscript must isolate stationary wave components, their amplitude modulation by precession, and the Rossby source function. None of these are analyzed.

9. A simple correlation analysis of banding vs ITCZ latitude, Hadley cell strength, Western Pacific Subtropical High latitude, and South Asian monsoon index would reveal whether Rossby waves are truly dominant. This is not done.

Specific comments

1. The title asserts a Rossby-wave–regulated mechanism at orbital scale, but the paper does not yet provide causal evidence. Consider softening the statement in the title until mechanistic diagnostics are strengthened.

2. Abstract over-claims: “planetary wave propagation constitutes the primary determinant.” This is not demonstrated quantitatively.

3. Figure 1 and proxy compilation, the band definition appears qualitative. A more objective classification would strengthen the identification of the hexapole pattern. Using only “representative records” from each band risks confirmation bias. The full dataset of 18 records should be analyzed statistically to determine whether banding exists independently of prior expectations.

4. Phase relationships in Fig. 1b require statistical confidence intervals; tuning uncertainties can reach several kyr and must be acknowledged explicitly.

5. Interpretation of S/(I+C) as precipitation intensity requires clearer justification. Weathering is influenced by lithology, vegetation, and erosion processes—not only rainfall.

6. EOF1 explains 86.5% of variance in 500 hPa geopotential height. This likely corresponds to large-scale monsoon intensity variations, or Hadley cell expansion/contraction, or shifts in seasonal thermal contrast. Rossby waves are patterns on top of mean-state circulation — not equivalent to EOF1. Interpreting EOF1 as a Rossby-wave diagnostic is not justified.

7. The patterns in Figs. 2c–f (precipitation, geopotential height, vorticity) match known monsoon variability structures. They are not uniquely attributable to Rossby wave propagation. Moisture advection and vertical motion anomalies are not isolated.

8. Wave activity flux arrows in Fig. 2g merely show that waves propagate under the resulting circulation anomaly. They do not demonstrate that waves are the source of the anomaly, or that waves are the mechanism mediating orbital-scale changes. The analysis is descriptive rather than diagnostic.

9. Sections on land–sea contrast dismiss its role too quickly; these mechanisms remain well-established drivers in the literature.

10. For the seasonality, good that authors acknowledge winter vs summer differences. But there is no mechanistic diagnosis of how seasonal mean changes shift stationary wave characteristics.

11. Conceptual figure 4 is plausible but schematic and not quantitatively demonstrated. If Rossby waves are key, authors should show anomalous heating centers, Rossby source function, and stationary wavenumber fields.

12. Wave activity flux formula is standard, but use of only 200 hPa level may miss low- to mid-tropospheric interactions. Insufficient detail on seasonal filtering and frequency filtering. No moisture budget diagnostics are performed.

Version 1:

Reviewer comments:

Reviewer #3

(Remarks to the Author)
The authors have made a serious and substantive effort to address the major concerns. Compared to the original submission, the revised manuscript is much stronger, especially in clarifying that Rossby waves are not acting on orbital timescales, but as seasonally recurrent stationary responses. They also added Rossby wave source (RWS), wave activity flux (WAF), and moisture budget diagnostics. The Methods section now is expanded to describe forcing, acceleration, and spin-up. Following the comments, they explicitly tested alternative mechanisms (ITCZ, Hadley, WPSH, SAM), and softened some of the more problematic causal language.

Several non-trivial issues remain and should be resolved before the manuscript is accepted for publication. See below.

1. The expanded Methods section is an improvement, but it raises new concerns that are not discussed critically enough. (1) Ice-sheet geometry and topography are prescribed from CLIMBER-2 and remapped every 100 years, but freshwater fluxes from ice sheets are explicitly excluded, despite their known influence on large-scale circulation. The justification ("to avoid underestimating AMOC variability") is not convincing without sensitivity testing. (2) The land–sea mask is fixed at LGM conditions for the entire 800 kyr simulation. This is a very strong assumption for a study claiming to diagnose pan-Asian hydroclimate mechanisms. The implications for monsoon pathways, coastlines, and moisture transport are not discussed. I understand it won't be possible to redo the simulation, but the authors need to provide a clear discussion of the limitations introduced by fixed LGM coastlines, absence of meltwater forcing, and use of a reduced-complexity ice-sheet product (CLIMBER-2). At minimum, the authors should explicitly state how these choices might bias Rossby wave propagation, moisture pathways, and band spacing and amplitude. At present, the Methods describe what was done, but not whether it matters for the conclusions.

2. The added PI control and PJ-pattern comparison (Figure S7) is helpful, but validation is still incomplete. The PI control uses LGM land–sea mask, not modern geography. Validation is largely limited to spatial similarity of the PJ pattern and qualitative agreement with CMAP precipitation. There is no validation of mean monsoon rainfall magnitude, seasonal cycle realism, vertical circulation structure, jet position and strength, moisture transport strength. Because the central claim depends on subtle circulation–moisture interactions, even moderate biases in monsoon strength, subtropical jets, amd background shear, could strongly affect the diagnosed Rossby-wave pathways. The manuscript still lacks a concise validation statement explaining what the model does well, what it does not, and why this does not invalidate the conclusions.

3. The authors now present a coherent diagnostic chain: IPWP convection → RWS → WAF → moisture advection → precipitation bands. This is a major improvement. However, all diagnostics are still regression-based and internally consistent, but not causal. No new sensitivity experiments are actually performed in this study. The argument relies heavily on analogy with the modern PJ pattern and previously published sensitivity experiments (Hu et al., 2024). The authord need to provide at least one of the following: a simple idealised perturbation within this study (e.g. suppressed IPWP heating), or a quantitative comparison showing that removing Rossby-wave-related circulation terms collapses the banding pattern. Without this, the Rossby-wave mechanism remains well-supported but not uniquely demonstrated.

4. The authors now argue that bands emerge from EOFs, boundaries are not sharp, and the pattern is a low-order meridional mode. This is reasonable, but EOFs are not equivalent to physical modes, and boundaries are still defined post hoc for budget analysis, the six-band structure is not shown to be statistically optimal, robust to domain choice, and stable across different filtering choices. A brief sensitivity test by changing latitude bins, or showing that the moisture-budget dominance does not depend on exact band definition, would be helpful. Without this, skepticism about "hexapole specificity" may remain.

5. The authors correctly clarify that they do not interpret sub-kyr variability, phase analysis is restricted to precession scale, and consistency across records supports robustness. This is largely satisfactory. However, the phase-wheel presentation (Figure 2) still visually invites over-interpretation. The text occasionally implies tighter phase coherence than the data strictly

allow. A short statement explicitly cautioning against over-interpreting phase leads/lags would further strengthen credibility.

6. The revised manuscript still occasionally suggests that the mechanism provides a “unified framework” for orbital hydroclimate variability. This remains too strong, given the reliance on a single model framework, strong boundary-condition assumptions, and lack of ensemble or inter-model confirmation. This should be clearly framed as a proposed mechanism, not a definitive explanation.

If these remaining issues are clearly and transparently addressed, I would be comfortable recommending acceptance after minor-to-moderate revision.

Reviewer #4

(Remarks to the Author)
Dear Editor, dear Authors,
Upon reading the manuscript, I identified flaws in the analysis, interpretation, and conclusions drawn from the data. As shown below, they concern the interpretation of the latitudinal spatial trend in phase relationships between the paleo-climate records and the astronomical solution for precession/insolation shown in Figure 2. These flaws preclude publication.
1) Rossby waves owe their existence to the stratification of the fluid and the variation with the latitude of the Coriolis frequency $f = 2\Omega\sin(\varphi)$ (Ω is the rotation rate of the Earth and φ is the latitude). Since $f=0$ along the equator (this should be considered a singularity in the equations of movement of Rossby waves), imagining a Rossby wave crossing the equator makes no physical sense (Figure 6). The equator can only trap Rossby and Kelvin waves.
2) The orbital forcing of the climate system can only be exerted with Rossby waves of very long apparent wavelength to be in line with their period. On the other hand, the variations in solar irradiance resulting from orbital cycles are so small that a positive feedback loop is necessarily involved in the amplification process. This is why the concept of gyral Rossby waves was introduced, as these waves act as a mediator between variations in solar irradiance and the climate response (the resonance occurs at 24,576 yrs).
Therefore, the phase-shifts observed in Figure 2 probably result either from diagenesis processes in the case of sediments, or from maturation processes within the epikarst in the case of speleothems. Under no circumstances can they be justified based on exogenous phenomena.

Pinault J-L, A Review of the Role of the Oceanic Rossby Waves in Climate Variability, J. Mar. Sci. Eng. 2022, 10, 493. https://doi.org/10.3390/jmse10040493
Pinault J-L, The Milankovitch Theory Revisited to Explain the Mid-Pleistocene and Early Quaternary Transitions, Atmosphere 2025, 16, 702. https://doi.org/10.3390/atmos16060702

Version 2:

Reviewer comments:

Reviewer #3

(Remarks to the Author)
The manuscript has been substantially improved following revision, and I appreciate the authors’ serious efforts in addressing the previous concerns. In particular, the clarification that Rossby waves are treated as seasonally recurrent atmospheric responses rather than orbital-timescale waves themselves significantly improves the physical consistency of the proposed mechanism. The addition of Rossby wave source (RWS), wave activity flux (WAF), moisture budget diagnostics, and the strong–weak Rossby-wave composite analyses provides much stronger dynamical support for the interpretation. The expanded discussion of alternative mechanisms (ITCZ, Hadley circulation, WPSH, monsoon circulation), together with the new sensitivity analyses on band definitions, also strengthens the robustness of the conclusions.

I also appreciate the authors' efforts to improve the Methods section, especially regarding the transient simulation setup, forcing implementation, and validation against modern circulation and moisture transport fields. The newly added validation figures and discussion help demonstrate that the model reasonably captures the large-scale circulation structures relevant to the proposed mechanism.

Overall, I find that my major scientific concerns have now been sufficiently addressed, and the manuscript is now much clearer, more balanced, and physically better justified. While some limitations remain inherent to the experimental design (e.g., fixed land–sea mask, absence of freshwater forcing, and lack of dedicated sensitivity experiments), the authors now discuss these limitations appropriately and avoid overextending the interpretation.

I therefore support publication after minor revision. Before final acceptance, I recommend the authors carefully clean the manuscript text, as the current version still contains a number of editing artifacts, duplicated wording, figure-number inconsistencies, and grammatical issues that appear to result from track-change merging. These are primarily editorial issues and do not affect the scientific merit of the study.

Reviewer #4

(Remarks to the Author)
Dear Editor, dear Authors,
Many thanks to the authors for their work clarifying the extratropical response of the upper atmosphere to equatorial Rossby waves resulting from deep convection processes. In particular, thank you for emphasizing the overlap of two timescales: one seasonal, the other of orbital origin.
However, ambiguity remains regarding the formation of extratropical Rossby waves moving poleward. This is not possible within the framework of the generally accepted theory of Rossby waves, which assumes that they are sensitive to latitudinal variations in the Coriolis parameter. But I cannot blame the authors because this is not always clear in the literature, unless redefining the concept of Rossby wave. I rather suggest the terminology " Extratropical divergence-convergence structures". So, this is a minor remark, and I leave the authors to their own judgment. The response of the upper atmosphere in the form of concentric waves around transient equatorial Rossby waves has been documented by several authors and the corresponding articles are well referenced in the manuscript.
I believe the manuscript meets the journal's criteria. In particular, it is innovative and of major interest to the field and related fields. The methodology is rigorous and clearly explained.

Open Access This Peer Review File is licensed under a Creative Commons Attribution 4.0 International License, which permits use, sharing, adaptation, distribution and reproduction in any medium or format, as long as you give appropriate credit to the original author(s) and the source, provide a link to the Creative Commons license, and indicate if changes were made.
In cases where reviewers are anonymous, credit should be given to 'Anonymous Referee' and the source.
The images or other third party material in this Peer Review File are included in the article's Creative Commons license, unless indicated otherwise in a credit line to the material. If material is not included in the article's Creative Commons license and your intended use is not permitted by statutory regulation or exceeds the permitted use, you will need to obtain permission directly from the copyright holder.
To view a copy of this license, visit https://creativecommons.org/licenses/by/4.0/

Response to Comments

Reviewer #1 (Remarks to the Author):

Detailed comments are attached as pdf file

My recommendation is major revisions

*What are the noteworthy results?

The noteworthy results of this paper are a climate model that demonstrates the potential role of Rossby waves in regulating Asian monsoon rainfall over orbital timescales. This is an overlooked/under-explored mechanism for explaining Quaternary paleo-climate change.

*Will the work be of significance to the field and related fields? How does it compare to the established literature? If the work is not original, please provide relevant references.

The work is original. It will be significant to paleo-climatology, modern climate change, geology, other geoscience fields, and perhaps environmental policy for anthropogenic contingencies.

*Does the work support the conclusions and claims, or is additional evidence needed?

The modeling work supports the hypothesis about Rossby waves. However, the research fails to exclude other hypotheses that explain the paleo-climate data. Therefore, instead of eliminating other possibilities, the research nominates an addition control on orbital patterns of Asian hydroclimate.

*Are there any flaws in the data analysis, interpretation and conclusions? Do these prohibit publication or require revision?

Revision is required. A main flaw is with the interpretation of the smectite data, which I provide detailed comments via the attached pdf. Also, the supposed latitude trend in phase relationships is just as easily explained by outlier data. There is not strong evidence, statistically or otherwise, for a latitudinal spatial trend in phase relationships between the paleo-climate records and the astronomical solution for precession/insolation.

Is the methodology sound? Does the work meet the expected standards in your field?

Yes

Is there enough detail provided in the methods for the work to be reproduced?

Yes

We sincerely appreciate your constructive comments, which have been instrumental in improving the overall quality and clarity of our manuscript. In the revised version, we have addressed your comments as follows:

(1) We have comprehensively summarized and discussed all potential factors that could

influence the interpretation of the paleoclimate data and the formation of the band-shaped precipitation anomalies. Please see lines 107-159 in the revised manuscript.

(2) Based on your comments regarding the origin of smectite, we have provided further clarifications and integrated this discussion into the main text. Please see lines 109-130 in the revised manuscript.

(3) We have meticulously filtered the entire collection of paleoclimate records (n = 18), and performed new correlation and phase wheel analyses specifically in relation to the precession cycle. Please see lines 157-159 in the revised manuscript.

All comments have been addressed point by point in the sections below, with detailed responses and corresponding line numbers indicated for clarity.

From attached PDF

Z. Yu et al. (submitted for review to Nature Communications) This paper presents a new dataset from the last ~150 kyr that adds to a growing body of Pleistocene paleoclimate information for the Indo-pacific warm pool and adjacent continental areas, particularly Asia. The authors also conduct climate modeling to contextualize anomalous observations that are thought to be not well explained by traditional forcing mechanisms of Indo-Pacific climate. My expertise on modeling is limited and therefore cannot comment on that aspect of the study.

The study attempts to understand why there are leads and lags in paleoclimate records from equatorial, to tropical, and finally subtropical Asia. The leads and lags are defined from correlations with the intensity of summer insolation modulated by the orbital cycle of climatic precession (20 kyr). An ultimate goal of the research is to further elucidate the underlying controls on the Asian monsoon so that we are better equipped to study changes in the monsoon that may arise from anthropogenic impacts.

The authors offer a novel and innovative hypothesis that the (1) offsets between the astronomical solutions, and the (2) spatial differences between the paleo-monsoon records are a function of Rossby waves that facilitate transport of moisture outward from the Indo-Pacific warm pool. Rossby waves deliver different rainfall amounts according to the latitude along a poleward (meridional) transect.

We extend our thanks for your expert insights and meticulous attention to our manuscript. Your feedback has greatly assisted us in formulating the overall concept of the entire theory, and particularly directly helped us address the reviewer's comments regarding the innovation of our manuscript.

Minor concerns

- Line 43: remove the article “the” from “… the hydrological circulation…”

Corrected

- The acronym OHC is not defined in the paper. [ocean heat conditions?]

Corrected

- Fig.1b: is "More rainfall" with the arrow just for record (3) or does this apply for all six records?

Which direction on the Y axis for the other five records also indicates more rainfall?

Yes, it applies for all records. We deleted this sign to avoid ambiguity. Meanwhile, we have added a sentence to the caption: "The upward direction of y-axis indicates more precipitation for records in Bands 1-6".

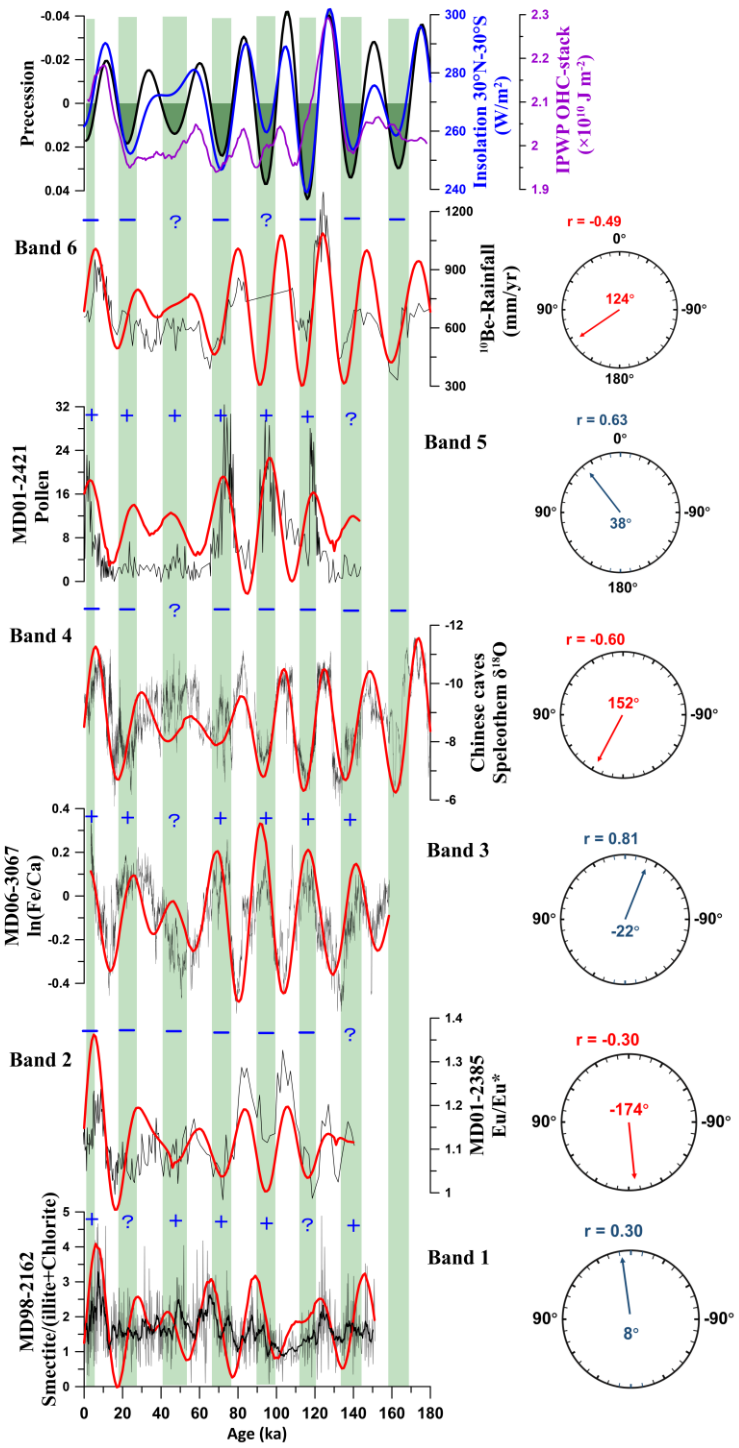

Figure 2 Comparisons of precession-paced precipitation changes from each Band. (1) Smectite/(illite+chlorite) ratios in MD98-2162 (this study); (2) Eu/Eu* ratios in MD01-2385 (Yu et al., 2024); (3) Ln(Fe/Ca) ratios in MD06-3067 (Kissel et al., 2010); (4) Stalagmite $\delta^{18}O$ from Sanbao Cave (Cheng et al., 2016); (5) Pollen from MD01-2421 (Yamamoto et al., 2005); (6) ^{10}Be of Chinese loess (Beck et al., 2018), and (7) Stacked IPWP upper ocean heat content (OHC) (Jian et al., 2022), insolation gradient from 30 °N to 30 °S and precession (Laskar et al., 2004). Note the precession axis is inverted. The red curves on top of each record are filtering curve of precession cycle. The upward direction of y-axis indicates more precipitation for records in Band 1-6. The degrees and r values noted on the phase wheel indicate the phase angle and correlation coefficient between the precession and each record during the 23-kyr cycle.

Slapdash concerns

Figure 4: incomplete/not labeled correctly according to the figure caption; no blue arrows or red arrows for example.

Corrected. Please see the following new Figure:

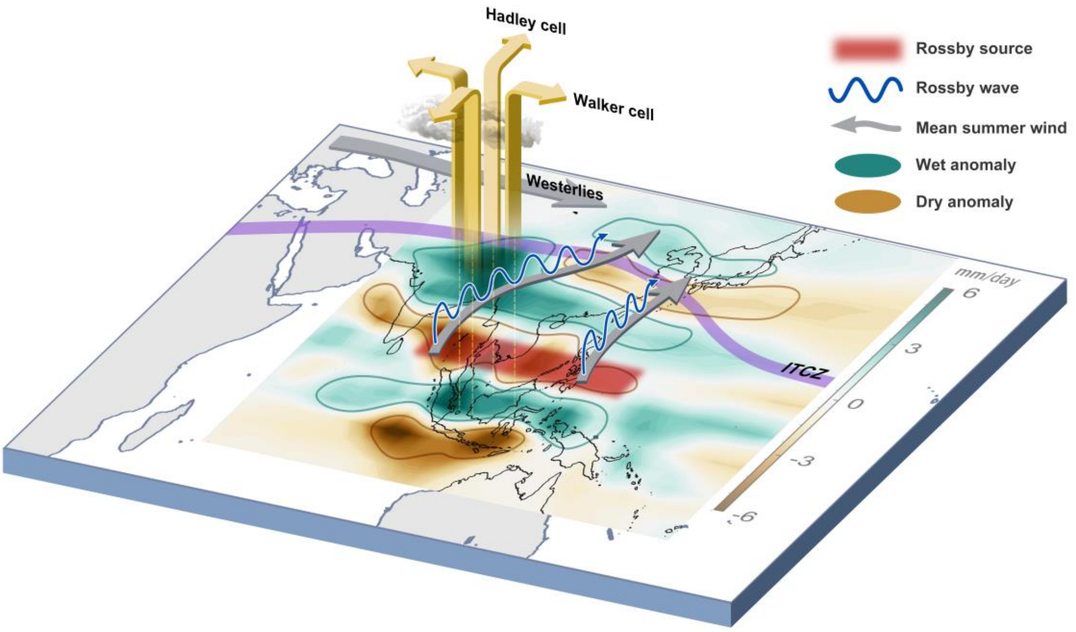

Figure 6 Schematic diagram of precipitation banding anomaly formation in the Asia-Pacific region. Precession-regulated insolation alters deep convection intensity (grey clouds) over the IPWP, generating a Rossby wave source (red shading) and upper-tropospheric (200 hPa) divergence (yellow arrows). The resulting meridionally propagating Rossby waves (blue curves) and moisture transport along the South Asian and western Pacific pathways (grey arrows) bridge tropical forcing to extratropical circulation, organizing coherent precipitation banding anomalies

Major concerns

1. To what extent do all the paleoclimate sediment cores have the same level of chronostratigraphy?

o Are the offsets due to the empirical error associated with chronology of each record?

o Six different records with six different climate proxy methods

▪ Why should they all behave the same?

▪ How about repeating the experiment using the same proxy method for all six rainfall

bands? Are the same results resolved?

We sincerely apologize for the lack of clarity in our chronological description in the original manuscript. We acknowledge the importance of robust chronology, especially for precession-scale studies. Please allow us to elaborate below.

The chronologies of all paleoclimate sediment cores analyzed in our study are appropriate for investigating orbital-precession variability. Specifically, the chronologies of the 18 paleo-precipitation records are primarily based on:

(1) U/Th dating with negligible errors (Cheng et al., 2016);

(2) AMS ^{14}C dating with age uncertainties ranging from several years to a few hundred years (Heaton et al., 2020);

(3) tuning of $\delta^{18}O$ profiles to the LR04 benthic stack (Lisiecki and Raymo, 2005), which provides an uncertainty of ~2–5 kyr for the past 1 Myr—an order of magnitude smaller than the precession period (~23 kyr).

Moreover, our study focuses on the most recent part of the last ~160 ka, for which the actual tuning uncertainty does not exceed ~2 kyr (Lisiecki and Raymo, 2005). Such tuned chronological frameworks have been widely and effectively adopted in previous studies to constrain phase relationships with the precession cycle, such as Figure 3e in (Jian et al., 2022), Figure 4 in (Clemens et al., 1996) etc.

To further address this point, we have performed new analyses in the revised manuscript. We applied Lanczos filtering (Duchon, 1979) and precession-phase analysis to all 18 compiled paleo-precipitation records (see Figures S1–4). The results demonstrate that records within the same precipitation Band exhibit essentially the same precession phase, even though they are based on independent chronologies and diverse proxies. This high consistency provides direct confirmation that the observed precession-phase difference between Bands reflects a genuine climatic signal rather than an artifact of chronological uncertainties.

We also greatly appreciate your valuable comments regarding uncertainties associated with different climate proxies. We fully acknowledge that this is a common challenge in paleoclimate reconstruction, as different proxies, influenced by distinct physical/chemical processes, can yield varying estimates of the same climatic variable (precipitation in this case). The prevailing approach to mitigating this issue is to compare results from multiple, independent proxies and to integrate proxy data with climate model simulations. In our work, we have implemented this strategy comprehensively:

(1) We have systematically compared results from the 18 records, as described above.

(2) The coherent precipitation-band pattern derived from the multi-proxy synthesis is further validated by simulations from the climate model. The strong agreement between independent proxies and the model supports the robustness of the reconstructed band-like precipitation anomaly pattern. The confidence level of this multi-method convergence is substantially higher than that achievable by any single proxy record alone.

In response to your comment, we have expanded the relevant section in the manuscript to better articulate these points. We believe the revisions and additional analyses have significantly strengthened the manuscript's foundation and clarity. Please see Figures S1–4 and lines 835-853, 1010-1016 in the revised manuscript.

The problem with the authors interpretation of smectite is that it is not simply warm, humid tropical climate equals greater smectite concentrations, as they posit. In tropical coastal environments that weather basalt rock (Eggleton et al., 1987) smectite in an early stage/less intense weathering product. The main weathering product is mixtures of kaolinite and Ferric oxyhydroxides, which increase under warm, humid climates. Smectite formation from basalt is favored by wet-dry climates (i.e., monsoons) that have a pronounced dry season, i.e., a net deficit of effective moisture (Kantor and Schwertmann, 1974; Deepthy and Balakrishnan, 2005; Zaarur et al., 2024). This is why, to me, it is not unexpected that the author's smectite/(illite-chlorite) ratio gets bigger under higher precession. Maxima in precession are associated with minima in tropical insolation and thus dry monsoons. Therefore, elevated smectite is correlating with dryness as one would expect and is not anomalous as the authors would lead us to believe.

We sincerely thank you for raising this important point and apologize for the insufficient explanation in our original manuscript regarding the critical differences between continental and island arc basalt weathering regimes.

The basaltic weathering studies you cited predominantly focus on stable continental or plateau settings, (e.g., Deccan Plateau from (Eggleton et al., 1987) and (Deepthy and Balakrishnan, 2005), Middle East from (Kantor and Schwertmann, 1974) and the southwestern part of Australia (Zaarur et al., 2024)). These regions are typically characterized by long sediment retention times, thick weathering profiles, and advanced chemical weathering stages. In stark contrast, the tectonically active tropical island arcs of Southeast Asia (our source region) represent a fundamentally different geomorphic and climatic regime: high relief, short river systems, steep slopes, and the absence of extensive alluvial plains (West et al., 2005). Under such conditions of intense physical erosion, increased precipitation primarily enhances sediment detachment and transport efficiency, drastically reducing the time available for in-situ chemical weathering in soils. Consequently, this regime delivers detrital minerals with notably lower degrees of chemical alteration, a process strongly documented for tropical island arcs where weathering is dominated by rainfall-driven physical erosion (Wu et al., 2012; Yu et al., 2024; Yu et al., 2023). Within this specific context, basaltic lithology under a perennially hot and humid tropical climate favors smectite neoformation, while the variations of illite and chlorite are interpreted to reflect background sedimentation. Changes in the

smectite/(illite+chlorite) ratio are linked to **variations in smectite input modulated by physical erosion intensity**, which is itself highly sensitive to precipitation changes (Wu et al., 2012; Yu et al., 2024; Yu et al., 2023).

Regarding the seasonality concern, modern climatology shows that even the "dry season" in Southeast Asian tropical islands sustains substantial rainfall (~200 mm/month) (Aldrian and Susanto, 2003), exceeding the peak monsoon precipitation in many mid-latitude regions (For example, north China, ~150 mm/month for June to September) (Wang et al., 2012). Thus, a true moisture-limited "dry season" is absent. This year-round warmth and moisture are prerequisite for smectite formation. While clay mineral assemblages globally are influenced by lithology, transport, and erosion, not solely by rainfall (Chamley, 1989; Huyghe et al., 2011), the unique combination of young, weatherable basalts and intense, perennial precipitation/runoff in tropical island arcs makes precipitation the dominant control on the erosional delivery of smectite in this specific setting (Wu et al., 2012; Yu et al., 2024; Yu et al., 2023).

In the revised manuscript, we have added a dedicated discussion on the uniqueness of Southeast Asian island arc weathering and refined the interpretation of our clay mineral proxy. Please see lines 109-133 in the revised manuscript.

2. The “Remarkable” phase shift phenomenon is an overstatement of the data

o So-called trends in phase angles are defined by an outlier in each case

▪ The speleothem and 10Be phase angles are effectively the same; the Eu/Eu* record is practically in-phase with precession; I don’t see a meridional trend, just the Eu/Eu* record is an outlier as compared to the other two record.

▪ Similarly, the pollen record is an outlier as compared to the new clay record and the XRF record.

▪ There are no trends just outliers

We thank you for your comment and fully agree with your assessment.

In response, we have performed filtering and precession-phase analysis on all 18 of the compiled paleo-precipitation records (Figures S1–4). **Overall, these analyses do not reveal a systematic phase shift between the different precipitation bands.** This lack of a significant inter-band phase shift may be because the stationary Rossby waves we posit primarily govern the existence of the ~10-15°-wide precipitation bands, but do not necessarily generate a consistent, progressive temporal lag (phase shift) between adjacent bands under the scale and proxy resolution of our records.

Given this result, we have **removed the sections of the main text that discussed a potential inter-band phase shift phenomenon**. We emphasize that this revision does not in any way alter the manuscript’s core conclusions, which center on establishing the existence, spatial structure, and dynamical mechanism of the orbital-scale hexapole precipitation system. Please see Figures S1–4 and lines 145-211 in the revised manuscript.

3. The six rainfall bands are not uniquely correlated to Rossby waves.

o The main areas of intensified rainfall are the south Asian landmass and the Maritime Continent.

o Weaker rainfall areas are present over the surrounding ocean waters

o The most parsimonious explanation for the data is that the authors have reproduced the rainfall patterns for the two largest precipitation “hotspots” of Asia. This is clearly an artifact of the seasonality of the monsoon.

o It would appear that of the hypotheses, the authors model data supports the hypothesis that the spatial differences in precipitation are due to land-sea thermal contrasts as proposed by Cheng et al. (Cheng et al., 2022). So I am not understanding why this simpler explanation of monsoonal dynamics is supplanted by the more complex argument of Rossby waves?

Thank you for your insightful comment. We agree that the dominant **absolute summer mean precipitation** pattern in the Asia-Pacific—featuring “hotspots” over South Asia and the Maritime Continent—is fundamentally shaped by the land-sea thermal contrast (Cheng et al., 2022). Our study does not challenge this established understanding.

Our work, however, focuses on the orbital-scale **summer precipitation anomalies** since 800 ka (derived as the difference between min- and max-precession composites in CESM 1.2 simulations), rather than on the mean precipitation distribution. In this anomaly field, we identify a **previously unidentified, 23-kyr periodic hexapole band pattern**, that is not a simple amplification or attenuation of the monsoon rainfall centers. Importantly, the spatial organization of these anomalies differs from the background monsoon pattern in three key aspects:

(1) The anomaly structure is distinct from the known large-scale monsoon background. The anomaly bands extend coherently across both land and ocean from ~20°S to 50°N, rather than being confined to continental precipitation hotspots.

(2) Such banded signals are absent in other contemporaneous meteorological fields (e.g., surface pressure, geopotential height, low-level winds; Figures 3–4), indicating that the signal reflects a dynamically organized anomaly rather than a direct thermodynamic response.

In this context, we interpret the land–sea thermal contrast as providing the **background thermodynamic state** and seasonal circulation(Kosaka and Nakamura, 2010), while proposing that Rossby wave dynamics act as a **regulating and organizing** mechanism that redistributes moisture and structures the precipitation anomalies meridionally. Specifically, precession modulated convection in the IPWP excites planetary-scale Rossby waves, which propagate meridionally through the summer atmospheric waveguide, redistribute moisture and reorganize the observed banding precipitation anomaly across latitudes.

To **quantitatively** verify the proposed Rossby wave dynamics, we have incorporated a suite of new diagnostic analyses, including **relative vorticity (RV), wave activity flux (WAF), the Rossby wave source (RWS)** at 200, 500 and 850 hPa, as well as **vertically integrated moisture fluxes and moisture budget** decomposition (see Eqs. (1-4) and

Figures 4–5). These analyses demonstrate that:

(1) Water vapor transport and its convergence-divergence patterns matches precipitation bands, confirming that summer precipitation anomalies across the mid-low latitudes are dominated by remote moisture transport forced by large-scale circulation, rather than local thermodynamic or dynamic processes.

(2) The spatial pattern of relative vorticity provides an effective indicator of Rossby wave phase characteristics: positive (negative) vorticity is associated with cyclonic (anticyclonic) circulation, corresponding to the trough (ridge) of the Rossby wave. Meanwhile, RWS diagnostics identify the **warm pool as the primary wave source** region, and WAF vectors demonstrate the poleward propagation pathway of Rossby waves in summer.

(3) **The essence of Rossby wave propagation is the transport of vorticity, most directly manifested as meridional wind anomalies (v').** Budget decomposition shows precipitation anomalies across multiple latitudinal bands are primarily associated with Rossby wave induced meridional moisture transport ($-v'\overline{q}$), rather than local thermodynamic forcing alone.

(4) In the South Asian (Band 4W) and Maritime Continent (Band 2) hotspots, **$-v'\overline{q}$ significantly exceeds $-u'\overline{q}$**, further supporting the Rossby wave modulated meridional transport plays a key role in shaping the anomaly structure, rather than zonal advection forced by the land-sea thermal contrast.

We have revised the manuscript to clearly distinguish between the background **monsoon-controlled precipitation climatology** and the **Rossby wave organized orbital-scale anomaly pattern**, and to elucidate the physical mechanisms underlying the banded precipitation pattern through these comprehensive diagnostic analyses. Please see lines 397-739 in the revised manuscript.

Reviewer #2 (Remarks to the Author):

Review on "Rossby wave–regulated orbital precipitation in the Asia–Pacific region" submitted to Nature Communications by Zhaojie Yu et al.

The study presented by Zhaojie Yu et al. investigates the relevance of orbital forcing for the distribution of precipitation in the Asia–Pacific region. The authors identify the precession cycle, operating on ~19–23-kyr timescales, as the most relevant orbital component when compared to obliquity and eccentricity. The study combines existing sediment records (representing precipitation in different latitudinal bands) with one new record (MD98-2162), together with climate model simulations conducted with CESM. The authors argue that the observed precipitation signals, in both sediments and model results, are predominantly driven by the precession cycle.

The authors further identify a hexagonal banded structure in the region of interest, which includes the Pacific–Japan tripole pattern. They argue that the observed pattern is shaped by anomalies in the Indo-Pacific warm pool, which modulate convection and the subsequent propagation of meridional Rossby waves. They conclude that orbitally forced IPWP anomalies have an analogue in a warming world and that their results may help constrain uncertainties in the region by better defining the range of naturally induced variability.

In general, the paper is well written. Some figures require substantial revision, and the text could be significantly streamlined in places. However, I find the choice of meteorological diagnostics difficult to understand and, at times, somewhat arbitrary. In its current form, the conclusions are, in my view, not clearly enough articulated, not sufficiently supported by the presented evidence, and I struggle to follow the overall narrative. Furthermore, uncertainties and statistical testing are insufficiently addressed in the present version of the manuscript.

My major reservation concerns the degree of novelty of the study. There already exists a considerable body of work on orbitally forced precipitation anomalies over Asia, and it is not yet clear to me what the specific selling point of the present analyses is that would justify publication in a highest-impact journal. The authors need to clearly communicate their unique selling point compared to the existing literature that would justify publication in such a high-impact venue. If the issues outlined below can be addressed in a thorough major revision, this work might become suitable for consideration for publication in Nature Communications.

We sincerely appreciate your insightful comments and constructive suggestions. We fully recognize the concerns regarding the clarity of the narrative, the diagnostic choices, the sufficiency of evidence supports, the uncertainties and statistical testing in the previous version.

In the revised manuscript, we have substantially restructured and streamlined the presentation to improve the overall narrative coherence. The manuscript is now reorganized around **a clear physical chain**: (i) identification of the robust and objective orbital-scale band-shaped precipitation anomaly pattern, (ii) diagnosis of the associated large-scale circulation anomalies connected with meridionally propagating Rossby

waves, and (iii) quantitative evaluation of the Rossby wave generation source, moisture transport pathways, and contributions to precipitation anomalies. Several sections have been condensed, and all figures reorganized to better align with this logical flow.

We have also clarified the physical reason for the choice of meteorological diagnostics. **Relative vorticity (RV), Rossby wave source (RWS), and wave activity flux (WAF)** are now explicitly introduced to diagnose Rossby wave phase, generation, and propagation, respectively (Eqs. 1-2 and Figure 4). The added **vertically integrated moisture flux and budget decomposition** (Eqs. 3-4 and Figure 5) provides a direct quantitative link between Rossby wave induced meridional circulation and the associated precipitation responses, while also quantifying the contribution of Rossby wave to the observed precipitation anomalies. Together, these analyses demonstrate how Rossby waves organize meridional moisture transport and shape the observed precipitation banding, rather than relying on schematic interpretation alone.

The novelty of our manuscript can be summarized into three points:

(1) **Identifying a Critical Discrepancy.** Prevailing understanding holds that Indo-Pacific hydroclimate changes are broadly in phase with orbital precession. Our MD98-2162 record directly challenges this view by demonstrating the opposite phase relationship.

(2) **Synthesizing Evidence to Reveal a New Pattern.** Motivated by this contradiction, we synthesized 18 paleo-precipitation records spanning low- to high-latitudes, leading to the discovery of a previously unidentified, 23-kyr periodic hexapole-band pattern in precipitation anomalies.

(3) **Proposing a Unifying Dynamical Mechanism.** To explain this novel banded pattern, we propose a new atmospheric waveguide mechanism. This planetary wave dynamics provided a unified dynamical explanation across different latitudes.

Finally, we have systematically addressed uncertainty and statistical robustness in the Methods and assessed the robustness of the identified signals across datasets and experiments. We have provided a detailed point-by-point response on your comments. Particularly, we have provided a detailed reply regarding the three key aspects of innovation in our reply to **Major Comment 1**. Furthermore, corresponding discussions have been added to the main text to further clarify these novel contributions. Please see lines 397-739 in the revised manuscript.

Major comments:

1. Sediment core analysis:

It is unclear to me what the inclusion of the new sediment core MD98-2162 adds to the overall findings. To my understanding, there are already plenty of sediment core analyses covering all relevant latitudinal bands. It is also not clear why some of the cores are included in Figure 1, while others are only shown in the Supplementary Material. This choice requires clearer motivation and justification, especially for readers who are not fully embedded in this specific field. It also needs to be discussed (even if only briefly) why the different sediment cores deviate so strongly in their lead–

lag times, even though they are, from a global perspective, rather close to each other.

We apologize for not clearly explaining the novelty of sedimentary record in MD98-2162. The novelty of our record and manuscript can be summarized into three points:

(1) **Identifying a Critical Discrepancy.** Prevailing understanding, based on the coherence between upper-ocean heat content and surface temperature in the warm pool, holds that Indo-Pacific hydroclimate changes are broadly in phase with orbital precession: precipitation minima occur at maximum precession (minimum insolation), and maxima at minimum precession (maximum insolation) (Jian et al., 2022). Our MD98-2162 record directly challenges this view by demonstrating the **opposite phase relationship—maximum precipitation occurs at maximum precession**. This key discrepancy raised fundamental questions about the spatial complexity of orbital-scale precipitation in the region.

(2) **Synthesizing Evidence to Reveal a New Pattern.** Motivated by this contradiction, we synthesized 18 paleo-precipitation records spanning low- to high-latitudes across the Asia-Pacific (Figures S1–4). This comprehensive analysis led to the discovery of a previously unidentified, 23-kyr periodic hexapole-band pattern in precipitation anomalies.

(3) **Proposing a Unifying Dynamical Mechanism.** To explain this novel banded pattern, we propose a new atmospheric waveguide mechanism. In this framework, precession-modulated convection over the Indo-Pacific Warm Pool excites planetary-scale Rossby waves. These waves propagate meridionally within the summer waveguide, redistributing precipitation and creating the observed latitudinal banding. This planetary wave dynamics reconciles long-standing discrepancies between proxy records and model simulations, providing a unified dynamical explanation for orbital-scale hydroclimate variability across different latitudes.

We have expanded the discussion of these innovative aspects both in our response to the specific comment and within the revised manuscript text. Please see lines 107-159, 145-211, 397-739 in the revised manuscript.

We fully acknowledge a fundamental challenge in paleoclimate reconstruction: different proxies record climate signals through distinct physical processes, inevitably leading to varying biases in their reconstructions—in this case, of past precipitation. The established approach to address this inherent uncertainty is the **integration of multiple, independent proxy records coupled with climate model simulations**. Therefore, we have taken concrete steps in our revised manuscript:

(1) **Multi-proxy consistency check**: We performed filtering and precession-phase analysis on all 18 compiled records (Figures S1-4). Despite relying on independent chronologies and different proxy types, records within the same identified precipitation band consistently show coherent phase relationships with orbital precession. This internal consistency across diverse methodologies strengthens the robustness of the banding pattern.

(2) **Climate model verification**: We further compared the multi-proxy synthesis with results from transient climate model (CESM) simulations. The model independently reproduces the spatial structure of the band-like precipitation anomalies, providing strong dynamical support for the pattern identified from the proxy data.

The convergence of evidence from this **multi-proxy, model-verified approach** yields a substantially higher confidence level than that with any single proxy record.

Regarding presentation: Displaying all 18 individual records in the main text is impractical, and mechanically averaging records with different inherent uncertainties is not methodologically sound. Therefore, for each of the six Bands, we have selected one representative record with the highest temporal resolution and most robust chronology for presentation in the main figures. The complete suite of 18 records is provided in the Supplementary Materials for full transparency and detailed inspection.

We have added corresponding text in the revised manuscript to explicitly discuss this multi-faceted validation strategy. This includes new figures and discussions that further clarify the innovative aspects of our work and elucidate how Rossby wave dynamics can account for the observed leading/lag times in sedimentary archives. Please see lines 79-84, 165-211, 397-739 in the revised manuscript.

2. The model used in the context of learning from the past for the future:

The proposed mechanism is based on the assumption that (positive) anomalies in IPWP heat content modulate convection and meridional Rossby wave propagation, which, shaped by the natural topography, ultimately produces the observed regional precipitation anomalies. The model resolution used here (3.75° horizontal spacing and 26 vertical levels; please also define the model top), however, is rather coarse, and one could question the ability of the model to realistically capture regional precipitation regimes, especially if Rossby wave propagation and complex topography are essential parts of the overall mechanism. Therefore, before drawing far-reaching conclusions, it is, in my opinion, essential that the authors demonstrate that the model is capable of reproducing precipitation anomalies associated with the PJ pattern during the contemporary era. This could, for example, be achieved by phase composites or comparable diagnostics. Furthermore, if the authors wish to draw conclusions along the lines of "learning from the past for the future," they should show how the model responds to future increases in greenhouse gases in the context of the proposed mechanism. The necessary data should be available; if this is not the case for dedicated future simulations, one could at least compare the latest decades of climate change to a situation around the 1850s (typically represented by pre-industrial forcing). This would really provide a sense of how the results differ between past, contemporary, and future climate states within the same modelling framework.

We extend our sincere thanks for your expert insights and evaluation, which have been invaluable in refining our research.

(1) **Model Validation and PJ Pattern Fidelity**. To assess the model's capability (3.75° resolution; model top at 2.3 hPa) in simulating the atmospheric teleconnection, we

further compared the **PJ pattern** from an added preindustrial (PI) control simulation with the modern observed PJ pattern (new added Figure S7). The comparison shows that the PI control simulation robustly reproduces the modern observed PJ pattern, further confirming the model's skill in capturing the essential large-scale teleconnection and meridional banded structure. Notably, our analysis focuses on large-scale precipitation anomalies and planetary-scale Rossby wave teleconnections, rather than local precipitation. Therefore, the key processes of Rossby wave generation, meridional propagation, and associated moisture redistribution operate at spatial scales that are well resolved in this model configuration.

(2) **Temporal Persistence of the Banded Structure**. Figure S7 further illustrates that while the **maxima of precipitation anomalies shift geographically** across timescales (western Pacific in modern, western Pacific–South China Sea in PI, and South Asia/Maritime Continent/Warm Pool on orbital scales), the **core banded spatial structure of precipitation anomalies is consistently maintained**. This underscores the remarkable dynamical persistence of this organized pattern across interannual, centennial, and orbital timescales.

(3) **A Unified Orbital-Scale Framework and Its Implications**. On orbital timescales, the banded precipitation anomalies align with the characteristics of both the PI and modern PJ patterns. Orbitally tropical insolation forcing through modulating the meridional propagation of planetary Rossby waves, establishes teleconnections that organize precipitation into bands from the tropics to mid-high latitudes. This indicates that the Asia-Pacific precipitation banding represents an **inherent, persistent circulation mode**. Furthermore, the orbital-scale banded anomalies serve as a **natural benchmark**, offering a vital reference for accurately quantifying the contribution of human activities to the intensification of modern and future PJ-type extreme events against the backdrop of natural variability.

We have incorporated these points and the new figures into the revised manuscript to strengthen the discussion. Please see lines 240-245, 879-894 in the revised manuscript.

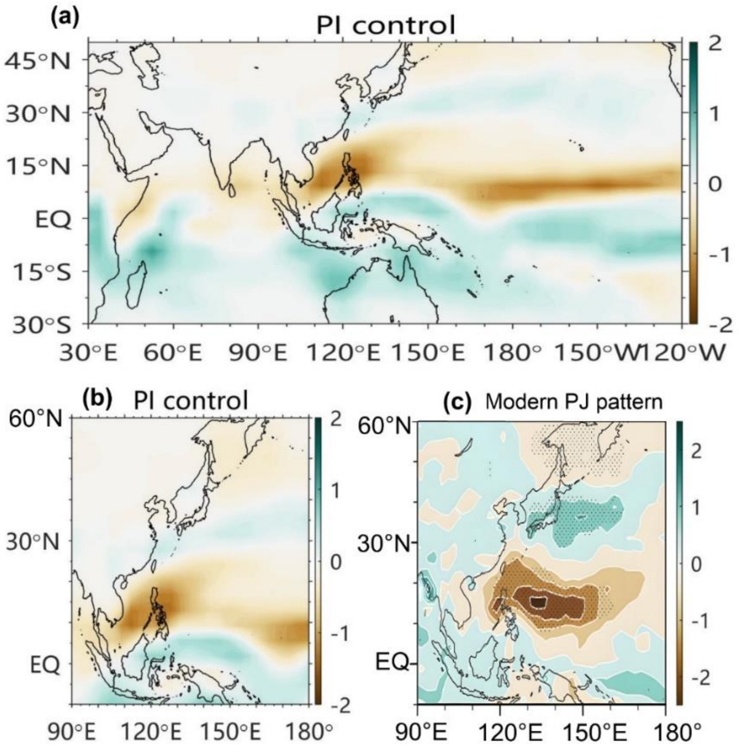

Figure S7 Structure of the Pacific-Japan (PJ) pattern. (a-b) Regressed summer precipitation anomalies using the identical methodology as in Figure 3c, based on the last 100-yr outputs derived from a preindustrial control simulation of the same model. The simulation is configured with fixed CO_2 concentration at 285 ppm, present-day orbital parameters and geographic configuration, but with the land-sea mask fixed at the Last Glacial Maximum (LGM) boundary. (b) shows the same pattern as (a) but focus on the core domain of the PJ pattern. (c) Regressed summer precipitation anomaly for 1979-2014 based on CMAP observational data (Xie and Arkin, 1997), illustrating the structure of the modern PJ pattern. This panel is from (Xie et al., 2016).

3. Inconsistent/arbitrary analysis underlying Figures 2 and 3, variability range and statistics:

In my understanding, the overarching idea is that, if the orbitally forced precipitation component is projected onto the climatological mean state of the summer dynamics in the region of interest, the emergence of the hexagonal structure can be understood. However, the meteorological variables shown in Figures 2 and 3 differ from each other. This makes it very difficult to follow the narrative. For example, Figure 2 shows precipitation, 500-hPa geopotential height, 200- and 850-hPa relative vorticity, and 200-hPa wave activity flux, whereas Figure 3 shows wind vectors from the surface to 200 hPa and 200-hPa velocity potential. Both analyses need to show the same quantities; otherwise, it is difficult to understand how the orbitally driven component differs from the mean state and how both interact. Please include the variables shown in Figure 2 in Figure 3, and vice versa. It is also unclear why the authors chose these specific levels. At this stage, the analysis appears somewhat ad hoc; a more consistent and unified framework is necessary, and the rationale for the chosen diagnostics and levels should be clearly explained in the main text or, at minimum, in the Supplementary Information.

In addition, all colour bars currently lack physical units; these should be included. Finally, Figures 1 and 2 show the signals associated with orbital forcing, but it is not clear how the magnitude of these signals (e.g. the precipitation response of ±2 mm day^{-1} in Figure 2c) compares to the overall variability of precipitation in this region. This needs to be shown to give the reader a sense of how large the contribution of orbital forcing actually is. Additionally, nowhere in the manuscript is it described how the significance tests are performed; it only states, for example, that results "pass the 95% confidence level." Which statistical test is used, and which software or toolbox is employed? Please provide this information.

We are truly thankful for your time and effort. Following your suggestions, we have made the following modifications to the main text:

(1) We have substantially revised and added new Figures 3-5 into a unified and consistent diagnostic framework linking orbital forcing, Rossby wave induced circulation response, and precipitation anomalies.

(2) In the new Figure 3, we have added precipitation and atmospheric circulation based on EOF regression analysis. This figure only displays the velocity potential at 200 hPa, because this level in the upper troposphere is a dynamically critical layer where the velocity potential signal is less susceptible to interference and exhibits a high signal-to-noise ratio. In the IPWP region, upper-level divergence at 200 hPa necessarily corresponds to lower-level convergence; thus, showing 200 hPa velocity potential is sufficient to characterize the divergent circulation associated with deep convection.

(3) In the new Figure 4, we have added wind vectors, relative vorticity, wave sources, and wave activity flux (WAF) at 850 hPa, 500 hPa, and 200 hPa (all atmospheric levels of monthly model output), representing low-level moisture transport, middle tropospheric wave structure, and upper tropospheric wave source/propagation, respectively.

(4) In the new Figure 5, we have added a vertically integrated moisture flux, convergence and budget diagnostic analyses, which elaborately quantify the Rossby wave induced moisture sources, pathways, thermodynamic and dynamic contributions for different precipitation Bands.

(5) We have added units to all color bars in Figures 1-5.

(6) We have added a new Figure S1b, which shows the comparison of precession-induced JJA precipitation anomalies and mean precipitation. The magnitude of summer precipitation anomalies in the extratropic (~6 mm/day) is close to the mean precipitation (< 6.75 mm/day). Figure S5 shows the proportion of precipitation anomalies on the precession timescale to total precipitation anomalies. In the East-South Asia region (60°E-140°E), this proportion exceeds 60%, with the warm pool region reaching up to 90%.

(7) In the Methods Section, we have added that the significance tests in the manuscript were all performed using the two-tailed Student's t-test, implemented through

MATLAB 2021 version.

Please refer to the description and illustrations in the manuscript and the figures as follows:

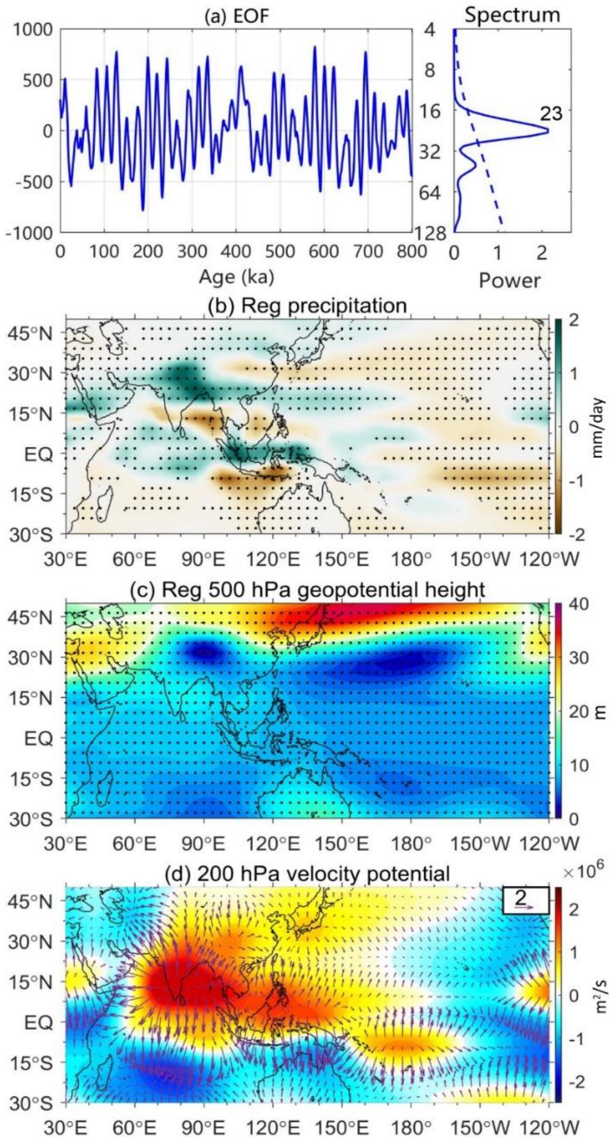

Figure 3 Precession impact on summer (JJA) precipitation and circulation changes since 800 ka. (a) Principal component (PC) timeseries associated with the first empirical orthogonal function mode of simulated JJA 500 hPa geopotential height anomalies and its power spectrum. Regressions of JJA (b) precipitation (mm/day), (c) 500 hPa geopotential height (m) and (d) 200 hPa velocity potential (shading; 10^6 m^2/s) and divergent winds (vectors; m/s) anomalies on the PC1 timeseries. The stippling in (c-f) indicate results that have passed the 95% confidence level.

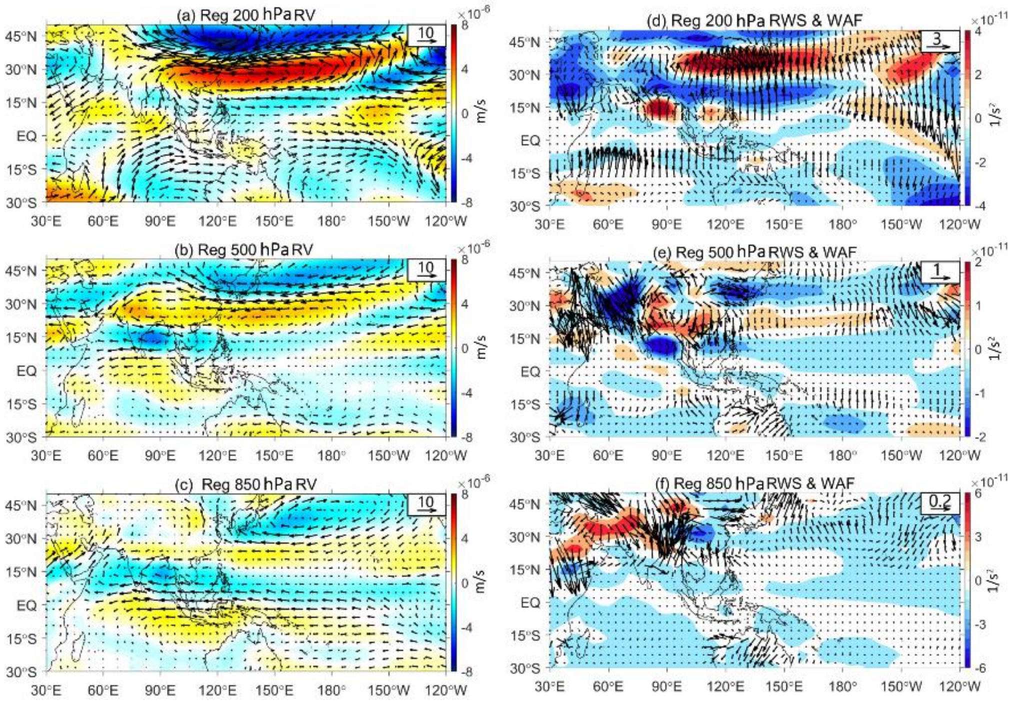

Figure 4 Rossby wave source diagnosis simulated (JJA) by transient CESM 1.2 since 800 ka. (a-c) Regression of JJA 200 hPa, 500 hPa and 850 hPa relative vorticity (RV, shadings; m/s) and wind vector anomalies (arrows; m/s) on the normalized PC1, respectively. (d-f) Regression of JJA Rossby wave source (RWS, shading; 1/s^2) and wave activity fluxes (WAF, arrows; m^2/s^2) at the same atmospheric levels. The stippling denotes values that are statistically significant at 90% confidence level.

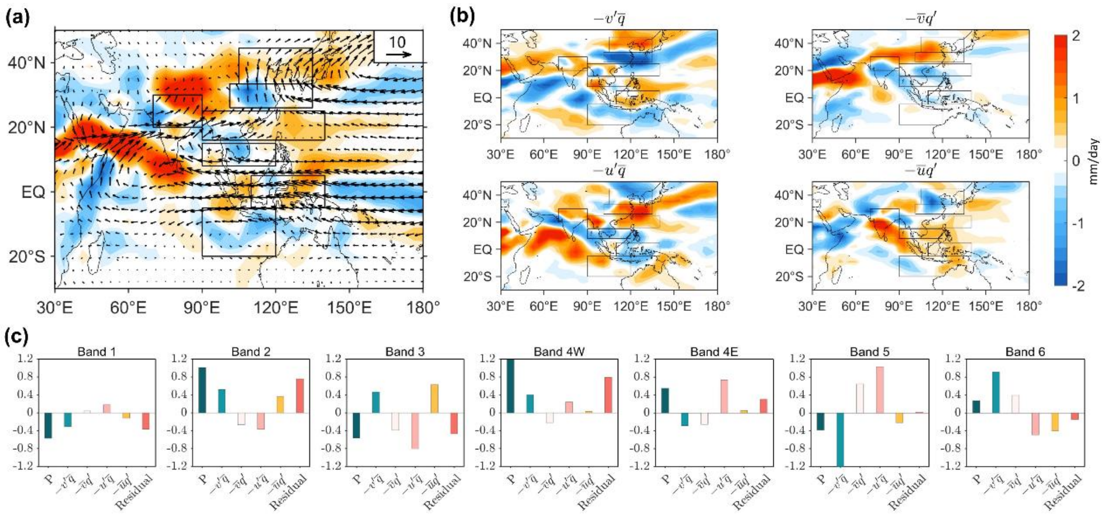

Figure 5 Water vapor budget diagnosis since 800 ka. (a) Regressed horizontal moisture fluxes (vectors: 106 kg/(m^2·s)) and divergence (shadings; mm/day) onto the normalized PC1 during JJA. (b) The first four terms (mm/day) of Eq. (4) from water vapor budget analysis. (c) Area-averaged regression coefficients of each term in (b) over the six precipitation bands, with Band 4 further divided into western (Band 4W) and eastern (Band 4E) sub-boxes. P and Residual in the x axis represent total precipitation and residual terms, respectively.

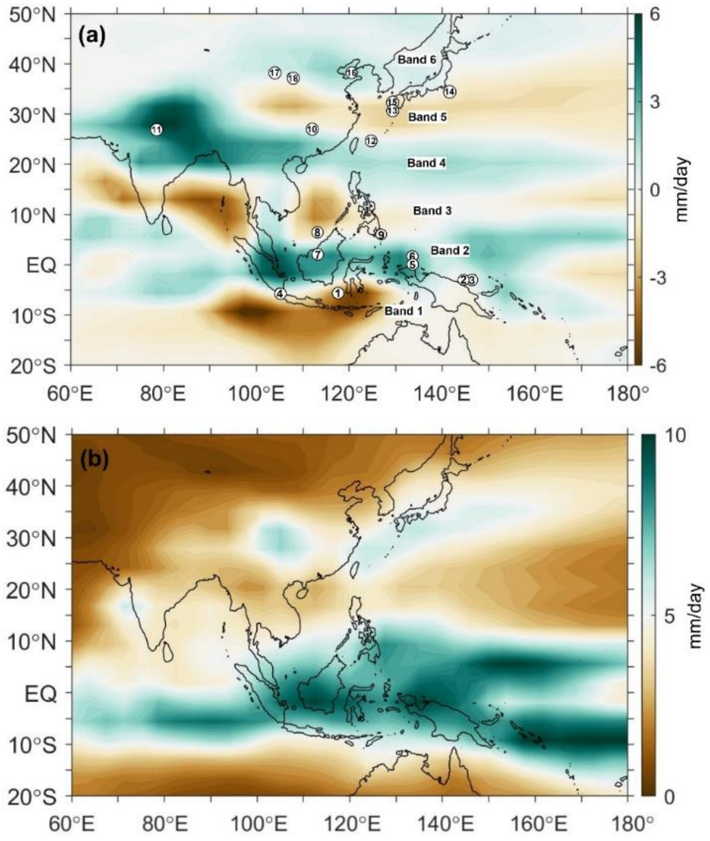

Figure S1 (a) Average summer (JJA) precipitation anomalies (mm/day) corresponding to the minimum minus maximum precession since 800 ka simulated by CESM 1.2 in the Asia-Pacific region. Band 1-6 indicate the hexapole banding areas. Circles and numbers represent the positions of the compiled precipitation records in Table S1 and Figures S2 and S3. (b) shows the mean precipitation (mm/day) distribution over 800 ka. Note that the color bars in (a) and (b) differ. Their comparison suggests that the magnitude of summer precipitation anomalies in the extratropics is close to the mean precipitation.

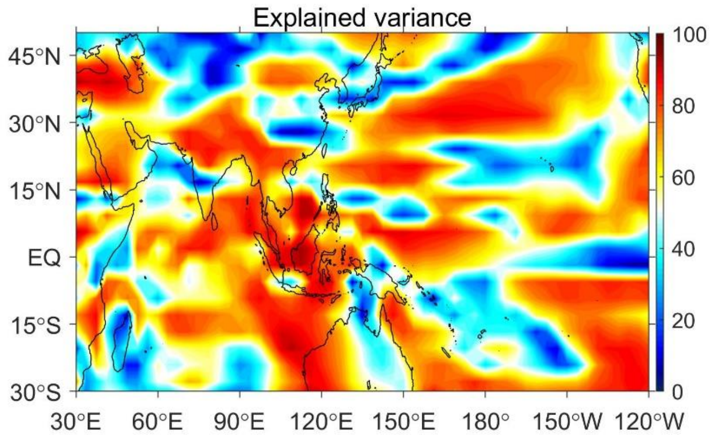

Figure S5 Explained variance (unit: %) of the 23-ka precipitation anomaly relative to the total precipitation anomaly.

Minor comments:

44: strike out “amounts”.

Corrected

46: add reference for statement on decline in precipitation.

Corrected

50–53: see comment on “learning from the past for the future”.

Thank you for raising this suggestion again. Please see the response and revision in Question 2 of the major comments.

58: maybe change “moisture” to “wet conditions”.

Corrected

60: briefly explain the reason for the spread in lags.

In the revised manuscript, we have conducted filtering and precession phase analysis on all 18 paleo-precipitation records collected (see Figures S1-4). The results show that the spread in lags does not apply to all the records, so we have removed this part of the discussion. The removal of these contents will not affect the core viewpoints of this manuscript.

62: clarify what is meant by “insignificant lag”.

Corrected

62–66: explain how this study contributes to addressing this lack of understanding; see major comment above.

Our study demonstrates that both the **modern Pacific-Japan (PJ) tripole pattern** and the **orbital-scale hexapole precipitation system** originate from a common dynamic explanation: **anomalous tropical insolation forcing** regulates the **meridional propagation of planetary Rossby waves**, establishing robust atmospheric teleconnections that redistribute moisture and organize precipitation from the tropics into the mid-to-high latitudes.

In essence, we propose that the **modern PJ pattern represents a spatially truncated manifestation of a broader, orbital-paced precipitation organization under contemporary boundary conditions**. This unified framework provides several key advances:

(1) **Theoretical Foundation for pattern Stability**: It explains the dynamical persistence of the PJ pattern across interannual to decadal timescales and establishes a **theory of modal stability** for this circulation regime. This offers critical theoretical support for mechanistic interpretation and long-term prediction of the modern PJ pattern.

(2) **Reconciling Paleoclimate Heterogeneity**: The framework helps reconcile apparent heterogeneity in past precipitation reconstructions across the Asia-Pacific. For example, it provides a coherent dynamic context for phenomena such as the tripole precipitation pattern observed in eastern China during the last

deglaciation (Zhang et al., 2018).

(3) **Anthropogenic warming Attribution**: The orbital-scale banded precipitation anomalies serve as a **natural variability benchmark** for PJ-type climate modes. This benchmark is essential for precisely quantifying the contribution of anthropogenic warming to the intensification of modern PJ-related extreme events.

We have incorporated this synthesizing discussion into the revised manuscript. For a detailed point-by-point response on the innovative aspects of this work, please also refer to our reply to **Major Comment 1**.

67: change "integrate" to "combine".

Corrected

69: explain why this record is essential for the overall narrative; see major comment above.

We have added the following sentence in the revised the manuscript to clear illustrate this at lines 81-84:

"The weathering proxy of this core, derived from the core region of the Indo-Pacific Warm Pool (IPWP), differs from marine precipitation proxies in controlling factors, thereby offering a new perspective for revealing the evolution of precipitation on surrounding islands (Yu et al., 2024)."

71: change "extracted" to "analyze".

Corrected

72: change "precipitation records" to "precipitation time series".

Corrected

Figure 1: design is somewhat unfortunate; it would fill a complete page in print. Consider redistributing subfigures. In 1a, the text (sediment locations) is not readable. Why do you show min–max, given that your record (1) suggests the opposite (e.g. positive response in band 1)? Please rearrange so 1a better follows your narrative. Your filtering method is nowhere explained; add details in the Methods section.

Follow your suggestions, we have split the original Figure 1 into two separate Figures 1 and 2. We also adjusted the precipitation record graph to be in Max-Min format (upper ocean heat content and insolation gradient from 30 °N to 30 °S) in Figure 2. We also added detailed Lanczos filtering (Duchon, 1979) in the Methods section. Please see the new Figures, Materials and Methods.

It is interesting to see that during high-precession periods surface solar insolation in the tropics is reduced, which appears to shape your overall pattern. This resonates well with studies suggesting a similar pattern under reduced solar activity based on climate reconstructions and state-of-the-art chemistry–climate modelling (Rimbu et al., 2024). Please consider discussing these findings, as they would strengthen your argument that naturally driven variability can indeed modulate long-term precipitation in this region.*

Thank you for your suggestion. We have included relevant comparative discussions in

the manuscript. Please see lines 564-571 in the revised manuscript.

95–98: here you explain the sediment record, but the composite maps show the exact opposite; please rearrange and clarify.

Corrected. Please see lines 107-159 in the revised manuscript.

118–145: justification that precession is the dominant component of orbital forcing is unnecessarily long; condense to 2–3 sentences and refer to the Supplementary Material for methodological details.

Corrected. We have moved some of the content of this paragraph to Materials and Methods and the Supplementary Material section according to your suggestion, while retaining only the description of the simulation results and the discussion on the effects of the three orbital cycles. Please see lines 165-195 in the revised manuscript.

218: why is this not shown? It would only add one figure to the Supplement and seems essential.

Added. Please see Figure S9.

220: clarify what is meant by “limited layers” (or correct the term if misspelled).

Thank you for pointing this out. We have deleted this term to avoid misunderstanding.

Figure 2: same quantities should be shown consistently in Figures 2 and 3. The colour bar in 2d is incorrect; it should be symmetric (e.g. −40 to 40) with the colour transition between blue and red at zero, otherwise it is very difficult to interpret. In general (as in 2c), it is conventional to show positive precipitation in blue and negative in red; please adjust.

Corrected. We have revised the figures to ensure full consistency in the displayed variables. We have expanded the content of Figures 2-3 into three new Figures 3-5 to present the analysis results for the same variables. Additionally, the colour bars have been corrected. We have adjusted the range and color scheme of the color bars (with the color scale for the precipitation field specifically optimized). Pleas also refer to the reply of **Major comments 3**.

Figure 3: see comments for Figure 2 and major comments above.

Corrected. Pleas also refer to the reply of above question and **the Major comments 3**.

238: consider replacing “combination” with “interaction”.

Corrected.

243: clarify what is meant by “annual wind field” (annual mean? climatology?).

We changed the “annual wind field” to “annual mean wind vector (m/s)”.

274: relabel subsection/title as “Topographic effects”.

Corrected

Figure 4: this figure can serve as an early version before submission but needs substantial revision. For example, the Rossby-wave-induced anomalies are currently

shown over the open ocean, but according to the manuscript they should be located over the continent to depict topographic effects.

Corrected. The schematic figure has been substantially revised to resolve potential misinterpretations. The revised schematic (new Figure 6) is not an idealized drawing but is directly based on the precipitation anomaly fields. It is intended to show the key features by highlighting the spatial organization of precipitation anomalies associated with Rossby wave induced circulation and moisture transport. Given the moisture source originates from the open western Pacific, the resulting precipitation anomalies extend over both continental Asia and the open ocean. The clarified figure is fully consistent with the proposed mechanism. Please see the new Figure:

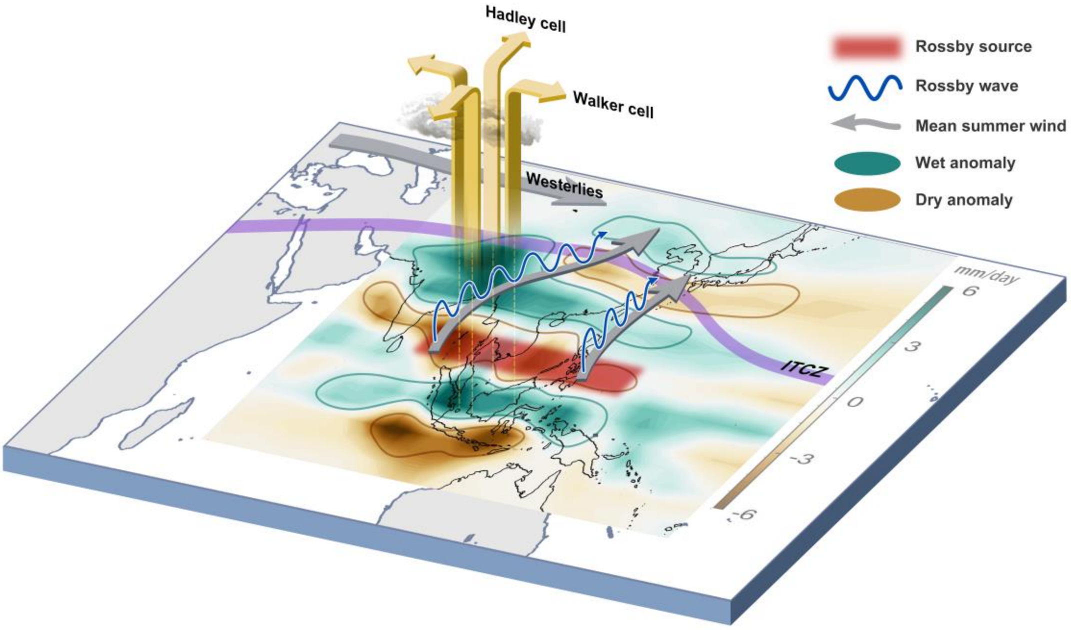

Figure 6 Schematic diagram of precipitation banding anomaly formation in the Asia-Pacific region. Precession-regulated insolation alters deep convection intensity (grey clouds) over the IPWP, generating a Rossby wave source (red shading) and upper-tropospheric (200 hPa) divergence (yellow arrows). The resulting meridionally propagating Rossby waves (blue curves) and moisture transport along the South Asian and western Pacific pathways (grey arrows) bridge tropical forcing to extratropical circulation, organizing coherent precipitation banding anomalies

316–317: you refer to the known PJ pattern here but need to show that the model can realistically capture it; see major comment above.

Added. Please refer to the reply of **the Major comment 2**.

References:

*Rimbu, N., Spiegl, T., Ionita, M., Doshi, S., & Lohmann, G. (2024). A synoptic scale perspective of solar forcing on extreme precipitation and floods over Europe during summer. Journal of Geophysical Research: Atmospheres, 129(21), e2024JD041952.

Reviewer #3 (Remarks to the Author):

This manuscript combines a new 150-kyr clay-mineral precipitation reconstruction from core MD98-2162 with an 800-kyr transient CESM1.2 simulation and proposes that orbital-scale hydroclimatic variability across the Asia–Pacific is regulated by meridionally propagating Rossby waves excited by precession-modulated IPWP convection. The authors identify a "hexapole" banding structure in modelled and reconstructed precipitation and interpret this as evidence for a planetary-wave–driven precipitation mechanism operating on orbital timescales.

The manuscript presents valuable new proxy data and an ambitious proxy–model comparison. However, the central physical mechanism — extension of synoptic-timescale Rossby wave dynamics to explain orbital-scale precipitation patterns, is not yet convincingly demonstrated. The evidence linking precession forcing to Rossby-wave energetics and propagation remains circumstantial, and several key diagnostics (moisture budget, stationary wave analysis, seasonal sensitivity, tests against alternative mechanisms) are missing. The manuscript therefore requires major revision before it can be considered for publication.

We sincerely thank you for the insightful and critical feedback on our manuscript. We fully acknowledge the validity of your concerns regarding the physical interpretation of extending synoptic-scale Rossby-wave dynamics to orbital-scale precipitation variability. We agree that precession forcing does not directly force Rossby waves, and that pattern correspondence alone is insufficient to establish a causal mechanism.

In response, we have substantially revised the manuscript to clarify the underlying physical framework and to incorporate multiple independent dynamical diagnostics. In the revised version, we explicitly clarify that transient synoptic Rossby waves cannot operate on orbital timescale. Instead, our focus is on the seasonally recurrent Rossby-wave response embedded in the summer mean state. We demonstrate that precession forcing acts by modulating IPWP convection and thermal background, thereby indirectly regulating the strength and propagation of stationary Rossby-wave responses that organize large-scale moisture redistribution.

To address these concerns, we have added comprehensive diagnostics—including Rossby wave source (RWS), wave activity flux (WAF), relative vorticity (RV), wave spectrum estimates, vertically integrated moisture flux and budget analyses (Figures 4-5), as well as seasonal sensitivity tests (Figures S12-13) and correlation of alternative mechanisms (Figure S11). Together, these revisions provide quantitative evidence that the observed banded precipitation anomalies arise from wave-mediated circulation and moisture transport under a precession-modulated mean state. Detailed responses to each of the reviewer's specific comments are provided below.

Major comments:

1. The central claim about meridionally propagating Rossby waves regulate precipitation on orbital timescales is not physically well-founded. Rossby waves are synoptic–seasonal atmospheric responses to instantaneous heating anomalies. Orbital forcing, by contrast, modulates seasonal mean states, not fast-timescale wave dynamics

directly. The manuscript does not establish how a fast atmospheric mode can be treated as the primary cause of slow climate variability. Without a clear theoretical framework linking mean-state changes to stationary Rossby wave structures, the interpretation risks being circular.

We appreciate the reviewer's concern regarding the apparent mismatch between fast Rossby-wave dynamics and slow orbital-scale climate variability. We fully agree that transient, synoptic-scale (days to weeks) propagation of individual Rossby waves cannot directly operate on orbital timescales.

Our central argument, however, addresses a different and more fundamental aspect: the **spatial organization** of precipitation anomalies, rather than its fast temporal variability. We do not invoke transient synoptic wave events operating on orbital timescales. Instead, Rossby waves are treated as **seasonally recurrent, quasi-stationary responses** embedded in the summer mean circulation, whose strength and structure are regulated by the precession-modulating slowly thermal background. Through stationary wave adjustment and repeated seasonal waveguiding, Rossby waves reorganize meridional moisture redistribution, imprinting a persistent, latitudinally banded precipitation anomalies when integrated over orbital timescales.

Within this framework, Rossby waves act as a **dynamical adjustment mechanism**: First, orbital precession does not act directly on fast wave dynamics. Instead, it modulates the interhemispheric insolation gradient (r = -0.90; Figure 2) and the associated deep convection and upper-ocean heat content over the IPWP. Second, this slowly varying thermal background regulates the **Rossby-wave response of the summer mean state**, including its source strength and meridional propagation characteristics. Third, through repeated seasonal stationary-wave adjustment and waveguiding, these circulation anomalies organize meridional moisture redistribution. When integrated over orbital timescales, this process yields a persistent, latitudinally banded precipitation pattern, rather than a snapshot of transient synoptic events.

To avoid circular reasoning, we now provide multiple independent diagnostics:

(1) Rossby Wave Source (RWS) and Wave Activity Flux (WAF) analyses identify the warm pool as the dominant wave source and demonstrate coherent meridional propagation (Figures 3-4).

(2) Moisture budget diagnostics further show that wave-induced meridional moisture advection dominates extratropical precipitation anomalies (Figure 5).

(3) Seasonal sensitivity analyses confirm that the mechanism is active only under the summer mean-state waveguide (Figures S12-S13).

(4) Sensitivity experiments in which warm-pool thermal forcing is suppressed show a marked weakening of both Rossby-wave activity and precipitation anomalies, demonstrating that tropical thermal forcing is a necessary precondition for the wave-mediated precipitation response (Hu et al., 2024).

We have integrated new corrections throughout the revised manuscript to elaborate on these points. Please refer to the new Figures and lines 252-686.

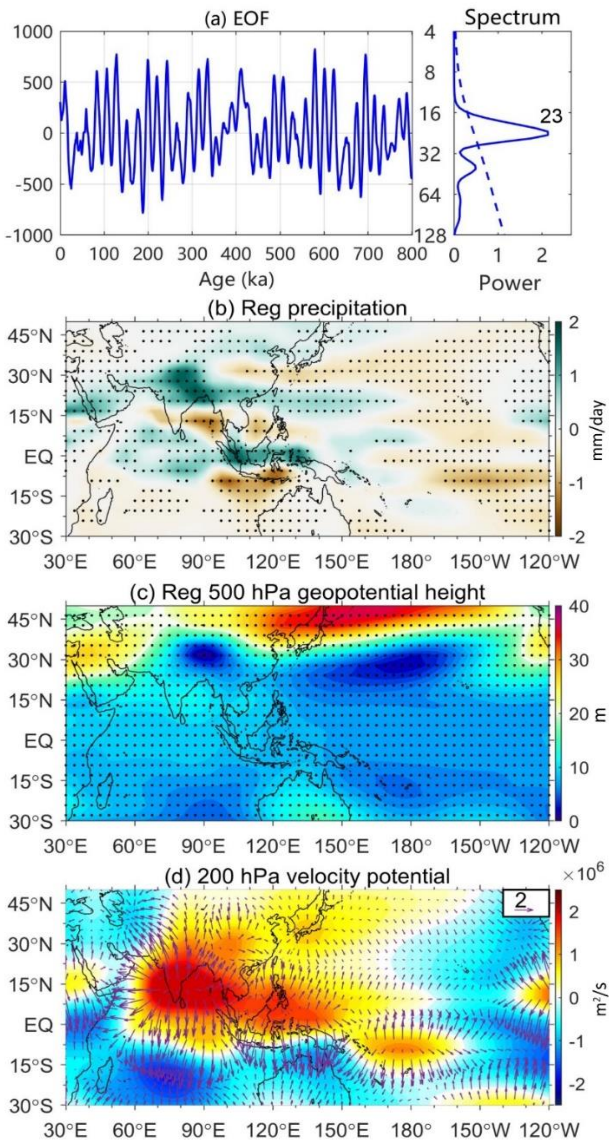

Figure 3 Precession impact on summer (JJA) precipitation and circulation changes since 800 ka. (a) Principal component (PC) timeseries associated with the first empirical orthogonal function mode of simulated JJA 500 hPa geopotential height anomalies and its power spectrum. Regressions of JJA (b) precipitation (mm/day), (c) 500 hPa geopotential height (m) and (d) 200 hPa velocity potential (shading; 10^6 m^2/s) and divergent winds (vectors; m/s) anomalies on the PC1 timeseries. The stippling in (c-f) indicate results that have passed the 95% confidence level.

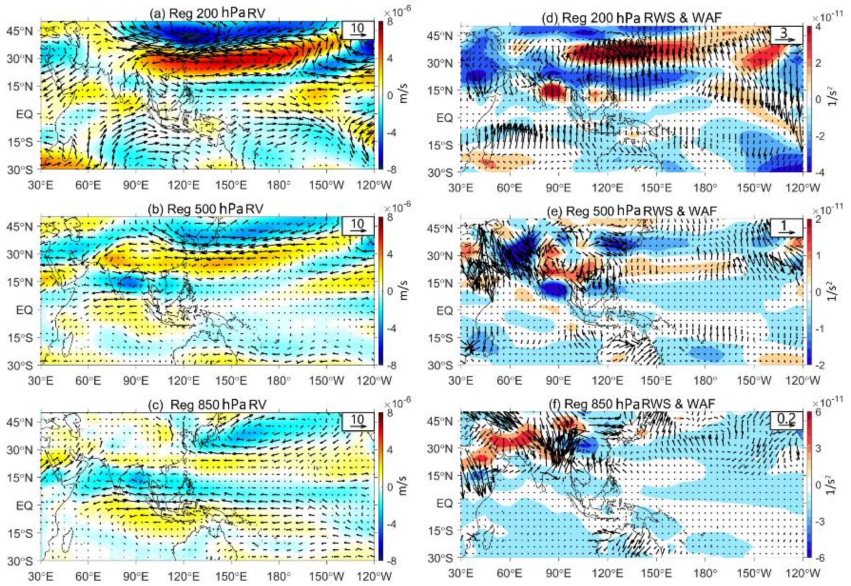

Figure 4 Rossby wave source diagnosis simulated (JJA) by transient CESM 1.2 since 800 ka. (a-c) Regression of JJA 200 hPa, 500 hPa and 850 hPa relative vorticity (RV, shadings; m/s) and wind vector anomalies (arrows; m/s) on the normalized PC1, respectively. (d-f) Regression of JJA Rossby wave source (RWS, shading; $1/s^2$) and wave activity fluxes (WAF, arrows; m^2/s^2) at the same atmospheric levels. The stippling denotes values that are statistically significant at 90% confidence level.

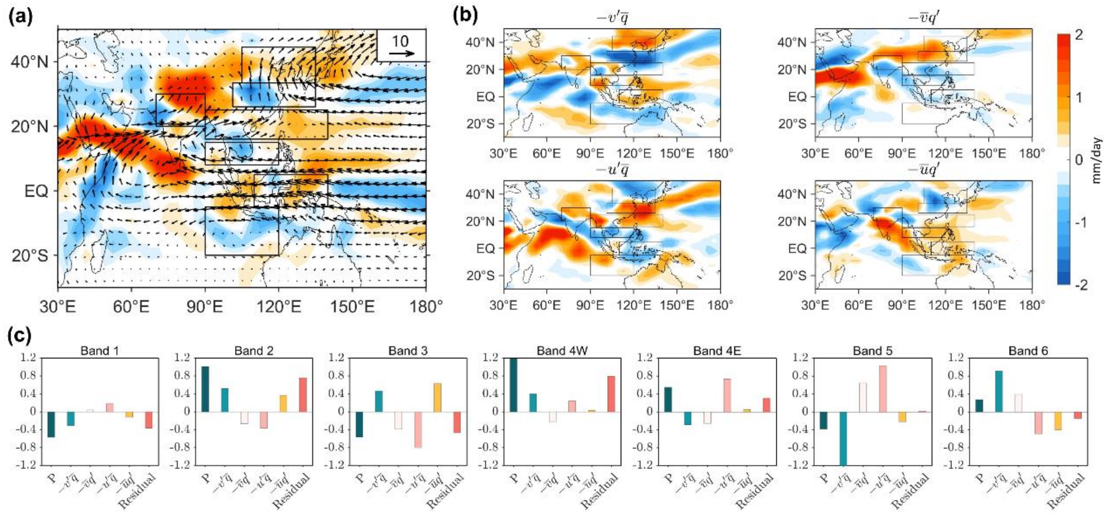

Figure 5 Water vapor budget diagnosis since 800 ka. (a) Regressed horizontal moisture fluxes (vectors: 10^6 kg/(m²·s)) and divergence (shadings; mm/day) onto the normalized PC1 during JJA. (b) The first four terms (mm/day) of Eq. (4) from water vapor budget analysis. (c) Area-averaged regression coefficients of each term in (b) over the six precipitation bands, with Band 4 further divided into western (Band 4W) and eastern (Band 4E) sub-boxes. P and Residual in the *x* axis represent total precipitation and residual terms, respectively.

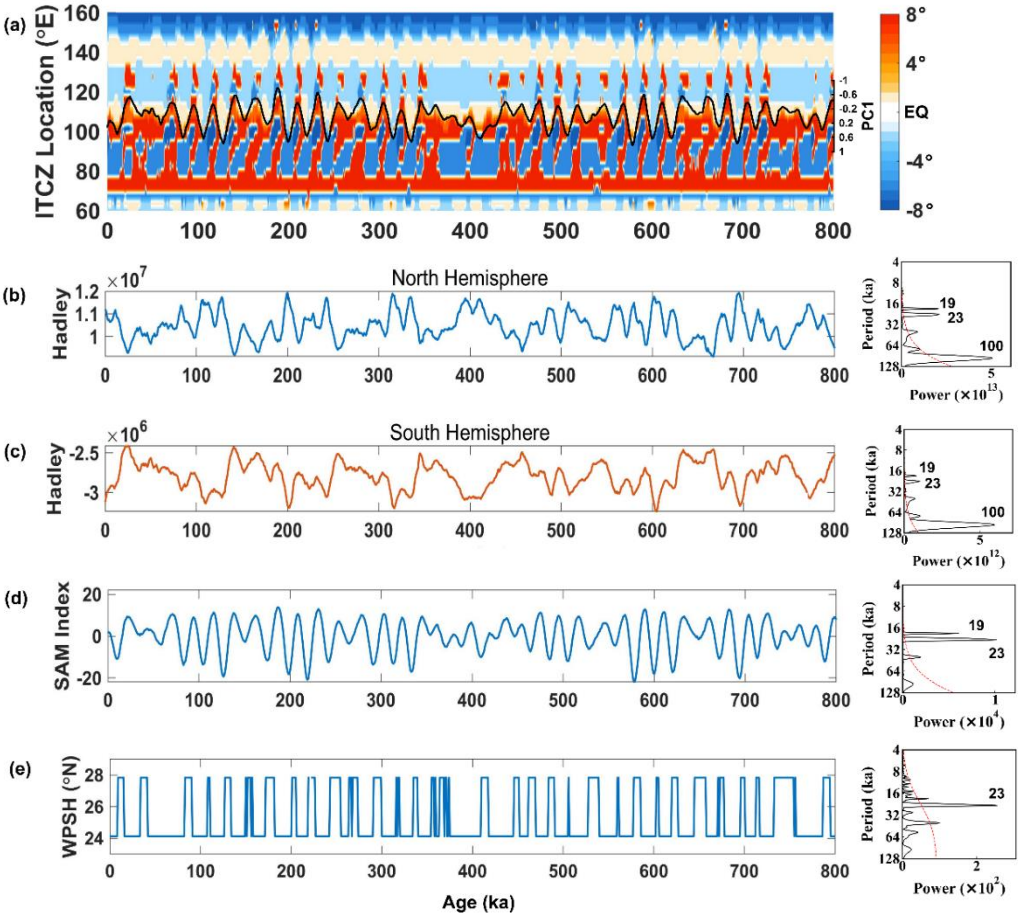

Figure S11 Relationships between summer (JJA) precipitation anomalies and potential atmospheric drivers from CESM1.2 simulations since 800 ka. (a) Latitudinal position of the ITCZ (unit: °); the black line denotes the time series of PC1. Intensities of the Hadley circulations in the (b) Northern Hemisphere and (c) Southern Hemisphere, respectively. (d) South Asian monsoon index. (e) Latitudinal extent (°) of the Western Pacific Subtropical High (WPSH). The right-hand panels present the periodicity analysis of the corresponding indices. Detailed definitions of these indices are provided in the Methods section.

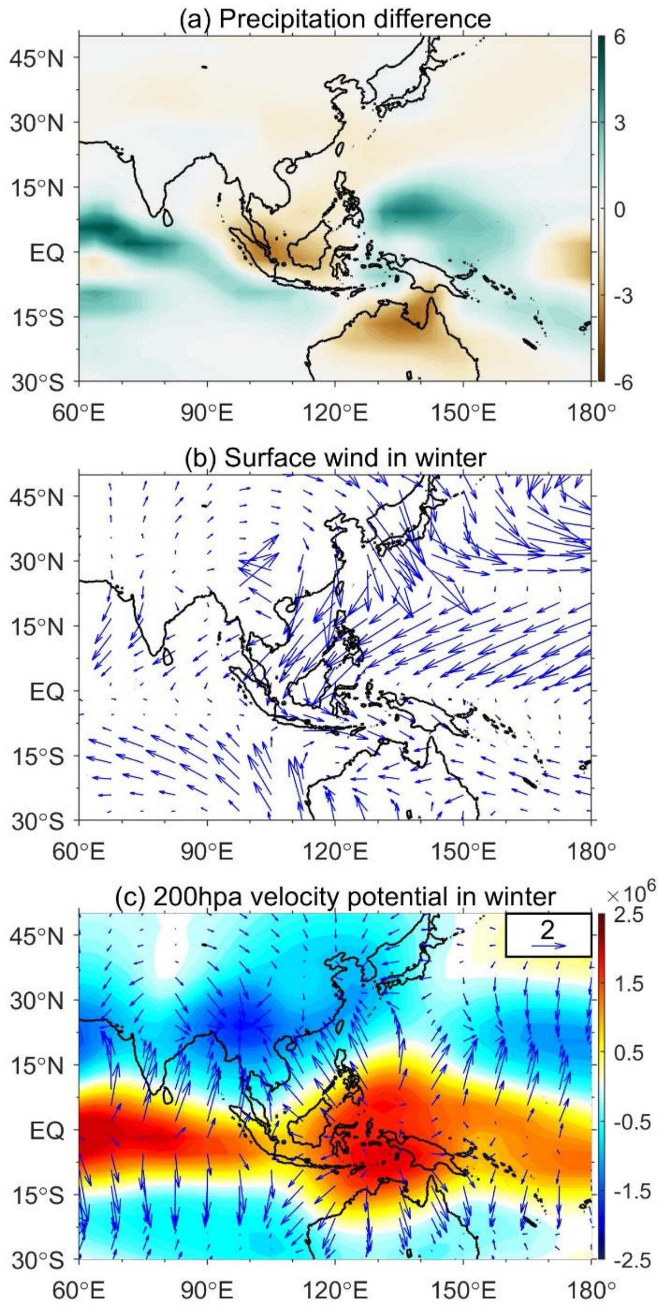

Figure S12 CESM simulation of precipitation and winds in winter (DJF). (a) The same as Figure 1 but for winter. (b) Surface wind stress (N m^{-2}) during winter. (c) 200-hPa velocity potential (shading; unit: 10^6 m^2 s^{-1}) and divergent wind (vectors). Positive velocity potential along the equator in winter are associated with strong precipitation, accompanied by the upper-level divergence. In contrast, the region of negative velocity potential between 15°N and 30°N reflects upper-level convergence.

2. Several well-established orbital-scale mechanisms may explain the latitudinal precipitation patterns: ITCZ migrations forced by hemispheric energy balance; land–sea thermal contrast and monsoon intensity changes; shifts in the westerly jet and Western Pacific Subtropical High; precession-controlled changes in tropical SST gradients and Walker circulation. These are briefly mentioned but not tested quantitatively. The current narrative assumes Rossby waves dominate without demonstrating that these alternative mechanisms are less significant.

We thank the reviewer for highlighting the importance of well-established orbital-scale mechanisms. We fully agree that land–sea thermal contrast, monsoon dynamics, ITCZ migration, tropical SST gradients, and large-scale circulation systems fundamentally shape the mean distribution of summer precipitation over the Asia–Pacific region.

In the revised manuscript, we now explicitly test these alternative mechanisms by examining their relationships with the banded precipitation anomalies, including the ITCZ latitude, Hadley circulation strength, South Asian monsoon intensity, and WPSH latitude (new Figure S10). We confirm the regional role of westerly jet influencing mid-latitude moisture advection in the midlatitudes (Figure 3). In addition, we clarify that the land–sea thermal contrast is a fundamental control on the **mean state** of regional precipitation and monsoon circulation (Cheng et al., 2022), rather than a direct forcing of the precipitation anomaly filed. While these factors exhibit precession variability, their impacts are **primarily regional or zonally confined** and cannot reproduce the **coherent, multi-latitude banded structure spanning 20°S–50°N**.

By contrast, diagnostics of RWS, WAF, and moisture budget (Figures 4-5) demonstrate that **Rossby wave–induced meridional circulation and moisture transport provide the dynamical linkage that integrates these regional forcings into a hemispheric-scale precipitation pattern**. Importantly, we do not interpret Rossby waves as a competing or alternative mechanism. Rather, they act as a **regulatory and integrative process**, redistributing moisture generated by tropical convection within the background circulation established by monsoons, ITCZ migration, and land–sea thermal contrast. In this sense, Rossby waves act as a dynamical organizer of precipitation patterns, **complementary to, rather than in competition with**, these alternative mechanisms. As verified by our water vapor budget analysis (Figure 5), meridional moisture advection term $-v'\bar{q}$ directly linked to Rossby waves contributes 59%–89% of precipitation anomalies in the 20°N–40°N band.

We have revised the manuscript accordingly to clarify this complementary mechanism. Please see lines 575-686.

3. The Methods section provides only a very brief description of the 800-kyr CESM1.2 transient simulation and refers readers to external publications for details. This level of description is inadequate for a study in which the model results constitute a major line of evidence supporting a new dynamical mechanism. Key external forcings, particularly ice-sheet geometry and topography, their source datasets, temporal resolution, and implementation strategy are not described. Also the treatment of CO_2 and other greenhouse gases, orbital forcing, coastline changes, and boundary condition interpolation is unclear. The manuscript also lacks essential information on model spin-up, drift behavior, and computational stability. As the model output forms a major component of the argument, some form of validation should be presented to demonstrate that the simulation realistically captures modern and orbital-scale monsoon dynamics and Rossby-wave patterns. Without transparent documentation of forcing fields, boundary-condition updates, and model performance, it is difficult to assess whether the simulated hexapole precipitation pattern reflects real climate dynamics or model-specific behavior. These issues must be addressed comprehensively

in the Methods section.

Thank you for your insightful comments and meticulous feedback. We agree that, given the central role of the transient CESM simulation in supporting our proposed mechanism, the Methods section requires substantially more detailed documentation.

In response, we have comprehensively revised and expanded the Materials and Methods section (Please see lines 879-902) to explicitly describe:

(1) all external forcings used in the transient simulation, including orbital parameters, greenhouse gas concentrations, ice-sheet geometry and topography, coastline changes, and their source datasets and temporal resolution;

(2) the strategy for boundary-condition updating;

(3) model spin-up procedure and numerical stability.

To address concerns regarding model fidelity and robustness, we further strengthen validation in two ways. First, the transient simulation framework follows (Yun et al., 2023), who demonstrated good agreement between CESM results and proxy records for large-scale monsoon evolution and orbitally paced hydroclimate variability. Second, to directly evaluate the dynamics most relevant to this study, we added a **new preindustrial (PI) control experiment** (fixed 285 ppm CO_2, present-day orbital configuration, with LGM land–sea mask; Figure S7). This experiment demonstrates that the model realistically reproduces the modern Pacific–Japan (PJ) pattern and its associated Rossby-wave teleconnection structure.

Importantly, we show that the orbital-scale Asia–Pacific precipitation banding shares consistent spatial characteristics with both the PI control and the modern PJ pattern, indicating that it reflects an intrinsic circulation mode across timescales, rather than a model-specific artifact. External forcings (e.g., ice-sheet configuration or SST anomalies) may primarily modulate the amplitude and position of precipitation anomalies, while the core dynamical linkage—tropical insolation forcing, Rossby-wave propagation, and low-to-high-latitude precipitation response—remains robust.

Together, the expanded methodological documentation and new validation experiments demonstrate that our modeling framework reliably captures the large-scale monsoon circulation, Rossby-wave teleconnections, and orbitally paced precipitation variability required to test the proposed mechanism.

"*Following the approach described in (*Yun et al., 2023*), we provide a description of the external forcings and boundary-condition updates applied in the 3Myr CESM1.2 transient simulation. Ice-sheet geometry and topography were prescribed from time-varying CLIMBER-2 simulation (*Willeit et al., 2019*) and remapped to the CESM grid at 100-yr intervals. The model did not include the ice-sheet freshwater forcing or calving from ice-sheets. This is because freshwater forcing originating from the ice sheets could possibly lead to an underestimation of the simulated Atlantic Meridional Overturning Circulation (AMOC) variability on orbital timescales. The land-sea mask and coastline are kept fixed at the LGM condition (21 ka). The 800 kyr Greenhouse-gas concentrations (CO_2, N_2O, CH_4) are prescribed based on the EPICA observations*

(Bereiter et al., 2015)*. To get a reliable climate sensitivity, the CO_2 forcing is scaled by a factor of 1.5. Greenhouse-gas forcing, Northern Hemisphere ice-sheet forcing, and orbital parameters (*Berger, 1978*) are updated every 100 model years. The model applied a relatively small orbital acceleration factor of 5 (for orbital, Greenhouse-gas, and ice sheet topography forcings), reducing orbital 3 million years into 600,000 CESM model years. The simulation was initialized from a fully spun-up 2300-yr preindustrial control run with minimal drift, ensuring numerical stability.*"

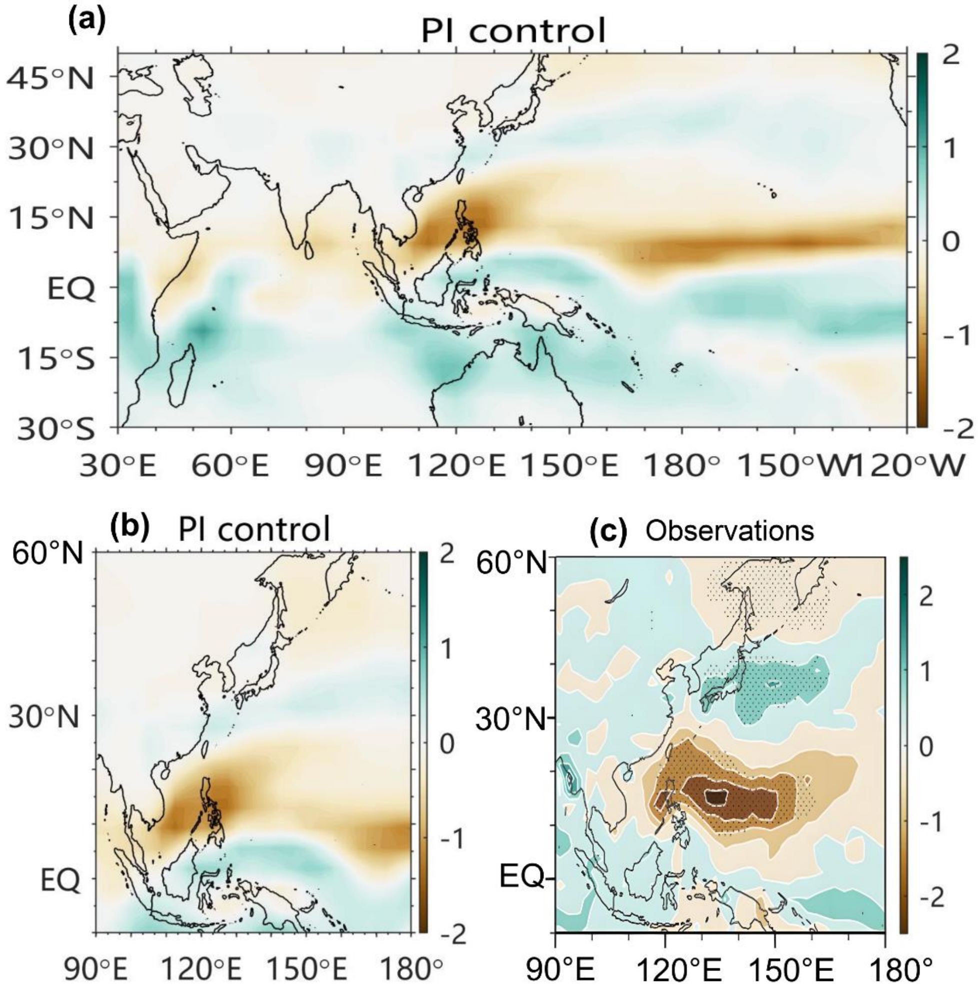

Figure S7 Structure of the Pacific-Japan (PJ) pattern. (a-b) Regressed summer precipitation anomalies using the identical methodology as in Figure 3c, based on the last 100-yr outputs derived from a preindustrial control simulation of the same model. The simulation is configured with fixed CO_2 concentration at 285 ppm, present-day orbital parameters and geographic configuration, but with the land-sea mask fixed at the Last Glacial Maximum (LGM) boundary. (b) shows the same pattern as (a) but focus on the core domain of the PJ pattern. (c) Regressed summer precipitation anomaly for 1979-2014 based on CMAP observational data (Xie and Arkin, 1997), illustrating the structure of the modern PJ pattern. This panel is from (Xie et al., 2016).

4. The six-band precipitation structure appears visually constructed and could be: (1) an artifact of model resolution; (2) related to the monsoon shear line or WPSH position, (3) or a zonal-mean precipitation response to orbital-scale ITCZ shift. No objective clustering or eigenmode analysis is shown. Without quantitative detection, the band

boundaries are subjective.

We deeply appreciate your meticulous evaluation and valuable suggestions. Below we respond point by point:

(1) Not as an artifact of model resolution

To test whether the model has the ability to accurately capture the banding precipitation, **we have added a PI control simulation** and conducted a multi-source comparison. As shown in the aforementioned Figure S7, the summer precipitation anomalies in the PI control and the transient orbital simulation exhibit strong spatial consistency with (i) the observed modern PJ pattern and (ii) independent proxy compilations spanning 18 cross-latitude records (Figures S1-4). This agreement across different timescales, forcings, and data sources indicates that the banded structure is a robust large-scale signal rather than an artifact of model resolution.

In addition, our study covers a broad Asia-Pacific domain (20°S–50°N, 60°E–180°, Figure 1). The estimated meridional Rossby wavelength associated with the precipitation anomalies exceeds 20° of latitude (Figure 4a), which is substantially larger than the model's horizontal resolution of 3.75°. Therefore, CESM has sufficient spatial resolution to resolve the identified precipitation bands and associated circulation anomalies.

(2) WPSH and (3) ITCZ shift

We agree that the WPSH and ITCZ migration are essential components of the Asia–Pacific summer climate system. In the revised manuscript, we explicitly test these mechanisms by examining the relationships between the banded precipitation anomalies and multiple established climate indices, including WPSH latitude, ITCZ latitude (Figure S11). The result shows that their impacts are primarily regional or zonally confined. In particular, ITCZ-related signals are largely restricted to the tropics, and WPSH-related influences mainly affect subtropical precipitation, but none of them reproduces the coherent, alternating wet–dry structure extending continuously from 20°S to 50°N.

We therefore interpret these mechanisms as shaping the background circulation and regional precipitation distribution, rather than serving as the primary organizing process of the full six-band pattern anomalies. This conclusion motivates the need for an additional dynamical linkage capable of coherently connecting tropical and midlatitude precipitation anomalies, which we demonstrate is provided by Rossby wave–mediated meridional circulation and moisture transport in the revised analyses.

(4) Objectivity of band boundaries

Importantly, the band boundaries are not subjectively drawn. The six-band pattern emerges consistently from multiple objective diagnostics, including: (i) the consistent **EOF decomposition** (i.e., eigenmode) of both summer precipitation anomalies (Figure R) and 500hPa geopotential height (Figure 2), (ii) **phase alignment** with relative vorticity and moisture convergence anomalies (Figure 4-5). The precipitation bands emerge objectively from the leading EOF modes, which represents a continuous, large-

scale eigenmode rather than a set of discrete regions. The band boundaries arise naturally from statistically defined features of this mode.

Although the precise latitudinal positions of these transitions are not sharp, reflecting the inherently continuous nature of large-scale atmospheric variability, the **anti-phase relationship between adjacent bands is statistically robust**. This alternating structure is consistently reproduced across independent diagnostics, including EOF analyses, large-scale circulation fields, and moisture convergence patterns (Figures 3-5). Consequently, the identified banded structure reflects a low-order meridional mode of the climate system, rather than an arbitrary or visually imposed segmentation of the precipitation field. Please see the new Figures 3-5 and lines 255-686.

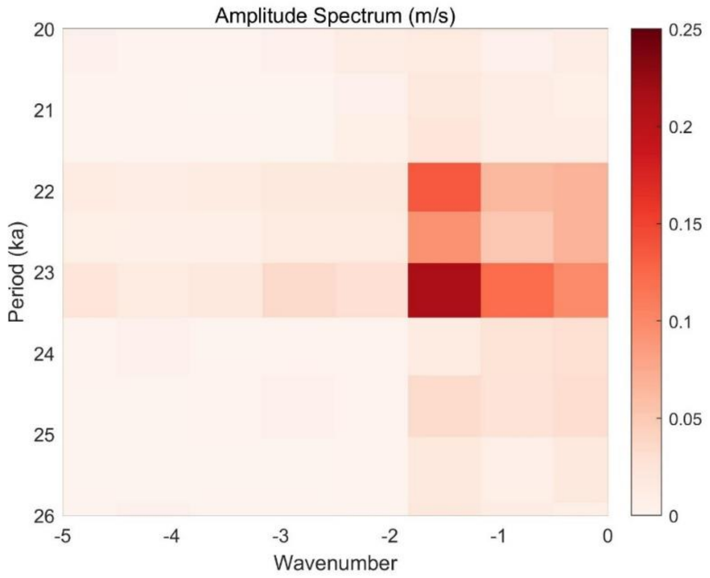

Figure S10 Rossby wave number-period spectral energy analysis of the 200 hPa JJA meridional wind anomaly (v') over 100°-120°E, EQ-60°N since 800 ka, calculated using a two-dimensional fast Fourier transform (FFT). The spectrum highlights dominant large-scale meridional variability on orbital timescales. Given the regional and zonally inhomogeneous nature of the circulation anomalies, these features are interpreted as low-order meridional wave structures rather than a single well-defined harmonic mode, consistent with the RWS–WAF diagnostics in the main text.

5. Many marine and terrestrial records used have tuning uncertainties of 2–5 kyr, comparable to the precession cycle phase. But Fig. 1b interprets sub-kiloyear lead/lag patterns across latitude bands. These phase relationships are likely not statistically meaningful.

We apologize for the lack of clarity in our chronological description in the original manuscript. We acknowledge the importance of robust chronology, especially for precession-scale studies. Please allow us to elaborate below.

The chronologies of all paleoclimate sediment cores analyzed in our study are appropriate for investigating orbital-precession variability. Specifically, the chronologies of the 18 paleo-precipitation records are primarily based on:

(1) U/Th dating with negligible errors (Cheng et al., 2016);

(2) AMS ^{14}C dating with age uncertainties ranging from several years to a few hundred years (Heaton et al., 2020);

(3) tuning of $\delta^{18}O$ profiles to the LR04 benthic stack (Lisiecki and Raymo, 2005), which provides an uncertainty of ~2–5 kyr for the past 1 Myr—an order of magnitude smaller than the precession period (~23 kyr).

Moreover, our study focuses on the most recent part of the last ~160 ka, for which the actual tuning uncertainty does not exceed ~2 kyr (Lisiecki and Raymo, 2005). Such tuned chronological frameworks have been widely and effectively adopted in previous studies to constrain phase relationships with the precession cycle, such as Figure 3e in (Jian et al., 2022), Figure 4 in (Clemens et al., 1996) etc.

To further address this point, we have applied filtering and precession-phase analysis to all 18 compiled paleo-precipitation records (see Figures S1–4). The results demonstrate that records within the same precipitation Band exhibit essentially the same precession phase, even though they are based on independent chronologies and diverse proxies. This high consistency provides direct confirmation that the observed precession-phase difference between Bands reflects a genuine climatic signal rather than an artifact of chronological uncertainties.

We also check throughout the manuscript to make sure that we did not discuss any sub-kyr variability. We added several sentences in the **Estimation of errors** section to illustrate it. Please see lines 1010-1016.

6. Clay mineral ratios (smectite/illite+chlorite) depend on lithology, erosion intensity, transport path length, and vegetation cover, not solely on precipitation. The manuscript overstates the linearity of this proxy–climate relationship.

We agree with your assessment that clay mineral ratios can be influenced by a combination of factors including lithology, erosion intensity, transport distance, and vegetation cover, and are not a sole function of precipitation.

However, within the **specific context of tropical island arcs in Southeast Asia** that are the focus of our proxy network, multiple studies have demonstrated that precipitation intensity becomes the dominant control on these ratios (Wu et al., 2012; Yu et al., 2024; Yu et al., 2023). This is due to a unique set of regional characteristics:

(1) **High-Energy Erosional Environment**: These islands are characterized by steep topography, short river systems, substantial altitudinal gradients, and the absence of extensive alluvial plains. This setting fosters a highly efficient physical erosion regime.

(2) **Precipitation-Driven Erosion Link**: In such a regime, increased precipitation directly enhances both physical erosion rates and sediment transport efficiency within the watersheds. This reduces the residence time of sediment in soil profiles, limiting the extent of chemical weathering.

(3) **Lithological Consequence**: The dominant basaltic lithology in these arcs, combined with the hot and humid climate, is particularly conducive to the formation

of smectite through weathering. Under the prevailing high physical erosion, the delivery of detrital smectite—a mineral indicative of less advanced chemical weathering—becomes closely tied to precipitation-modulated erosion.

Consequently, variations in illite and chlorite primarily reflect background sedimentation, whereas changes in the smectite/(illite+chlorite) ratio are interpreted as reflecting fluctuations in smectite input governed by physical erosion, which is itself precipitation-controlled.

We have added a brief discussion in the manuscript to clarify this regional framework and justify the application of clay mineral ratios as a precipitation proxy in our study area. Please see lines 107-131.

7. There is no breakdown of precipitation anomalies into dynamic moisture advection, thermodynamic moisture changes, vertical velocity anomalies, stationary eddy contributions. Without this, the driver of precipitation changes cannot be identified.

We agree. In response, we have substantially expanded the moisture budget analysis to explicitly decompose the precipitation anomalies. In our moisture budget, the precipitation anomalies are defined as seasonal-mean (JJA) composites of precession-minimum minus precession-maximum. As a result, transient synoptic eddies are effectively filtered out, and the diagnosed circulation anomalies primarily represent stationary components.

We have performed comprehensive **a vertically integrated water vapor budget** diagnose (Eqs. 3-4; Figure 5), in which precipitation anomalies are decomposed into:

(1) Dynamic moisture advection associated with anomalous circulation acting on the mean moisture field $-\nabla \cdot (V'\overline{q})$;

(2) Thermodynamic moisture effects arising from mean circulation acting on anomalous moisture $-\nabla \cdot \left(\overline{V}q'\right)$; and

(3) Vertical velocity related contributions are diagnosed through the anomalous moisture convergence linked to large-scale ascent and descent (Figure 5a).

The revised diagnostics show that the precipitation anomalies are primarily dominated by circulation-induced moisture redistribution, rather than local thermodynamic amplification alone. The horizontal moisture flux and its divergence collectively delineate two meridional pathways of moisture transport modulated by these waves (Figure 5a). In particular, meridional dynamic moisture advection $-\nabla \cdot (v'\overline{q})$ associated with stationary Rossby-wave–induced circulation anomalies (v') accounts for approximately 59-89% of the anomalous precipitation over the mid-latitude Asia–Pacific region (Figures 5b-c).

The newly added quantitative budget analyses identify the dominant driver of the precipitation anomalies and demonstrate the organizing role of stationary wave modulated circulation in redistributing moisture from tropical to extratropical regions. Please see lines 431-535.

8. To claim a Rossby-wave mechanism, the manuscript must isolate stationary wave components, their amplitude modulation by precession, and the Rossby source function. None of these are analyzed.

Thank you for your constructive feedback. We agree that a Rossby-wave mechanism requires explicit diagnosis. In the revised manuscript, we now address all these aspects quantitatively.

(1) **Stationary wave components:**

In our work, the precipitation anomalies are defined as seasonal-mean (JJA) composites of precession-minimum minus precession-maximum. As a result, transient synoptic eddies are effectively filtered out, and the diagnosed circulation anomalies primarily represent stationary wave components. They are now explicitly diagnosed using the newly added relative vorticity, RWS and WAF (Figures 4).

(2) **Amplitude modulation by precession**:

We assessed the contribution of precession-forced precipitation anomalies to the total precipitation anomalies. The magnitude of summer precipitation anomalies in the extratropic (~6 mm/day) is comparable to the climatological mean (< 6.75 mm/day; Figure S1), indicating a significant signal. Within our study region (90°E–140°E), precession accounts for over 50% of the variance at most grid points, with contributions exceeding 80% in some areas (Figure S5). This result robustly demonstrates that precession is the dominant factor modulating precipitation anomalies in this region.

(2) **Rossby wave source and propagation**:

The spatial pattern of the leading EOF1 of summer precipitation (Figure S8c) closely matches the EOF1 obtained by regressing this precipitation anomalies onto the 500 hPa atmospheric circulation field (Figure 3). This coherence indicates that the banded precipitation anomalies are **governed by large-scale atmospheric circulation**, rather than local processes. The corresponding relative vorticity field clearly delineates alternating cyclonic and anticyclonic anomalies, identifying the positive and negative **phases of a planetary-scale Rossby wave** pattern (Figures 4a-c).

We explicitly diagnose the Rossby Wave Source (RWS), which reveals a pronounced upper-tropospheric (200 hPa) RWS anomaly over the IPWP (Figure 4d), identifying it as the primary wave-generation region. Consistent with this source, the WAF vectors clearly trace the subsequent meridional propagation of wave energy from the tropics into the Asia–Pacific midlatitudes (Figure 4d), completing the dynamical linkage between tropical forcing, Rossby-wave adjustment, and the observed banded precipitation anomalies.

Together, these diagnostics establish a clear dynamical chain linking precession-modulated tropical forcing, stationary Rossby wave generation and propagation, and the resulting banded precipitation anomalies.

We have added new analysis and refined the concluding statement to precisely capture the mechanism's role. Please see lines 397-417.

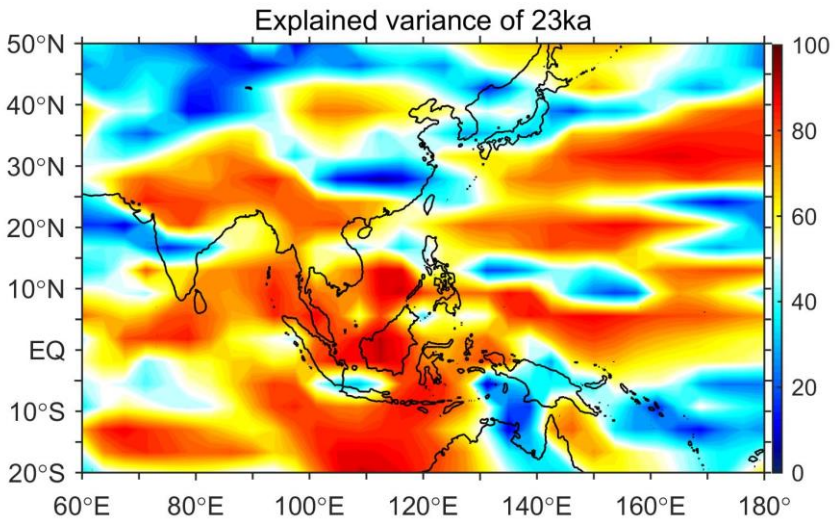

Figure S5 Explained variance (unit: %) of the 23-kyr precipitation anomaly relative to the total precipitation anomaly.

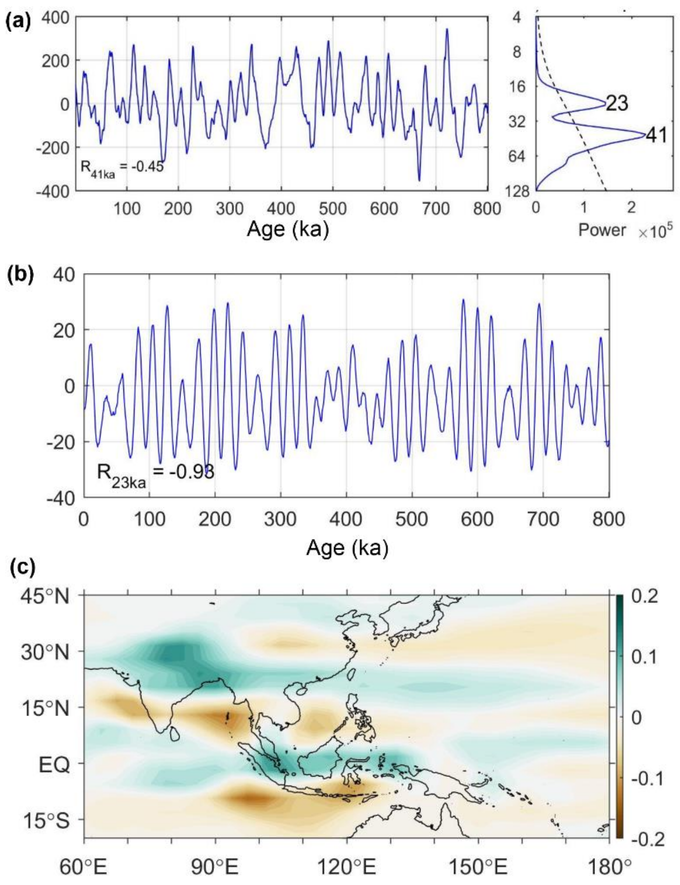

Figure S8 (a) As Figure 3a but for EOF2. The variance explained by EOF2 is relatively low at only 12.2%. (b-c) The leading mode of EOF analysis on simulated precipitation anomalies. The explained variance is 55.4%.

9. A simple correlation analysis of banding vs ITCZ latitude, Hadley cell strength, Western Pacific Subtropical High latitude, and South Asian monsoon index would reveal whether Rossby waves are truly dominant. This is not done.

We agree. In the revised manuscript, we have added a correlation analysis of the banded precipitation anomalies and climate indices, including ITCZ latitude, Hadley cell strength, WPSH latitude, and the South Asian monsoon index (new **Figure S10**).

Our results show that although these mechanisms exhibit precession variability, their impacts on precipitation are primarily regional confined and cannot reproduce the coherent, multi-latitude banded structure spanning 20°S–50°N.

Importantly, Rossby waves are **not presented as an alternative or competing mechanism** for band shaping. Instead, they act as a critical meridional moisture transport and dynamical adjustment process that **organizes and integrates** the precipitation patterns initially shaped by these background climate drivers, ultimately producing the observed six-band structure.

Please also refer to the response of **Major Comment 2**.

Specific comments

1. The title asserts a Rossby-wave–regulated mechanism at orbital scale, but the paper does not yet provide causal evidence. Consider softening the statement in the title until mechanistic diagnostics are strengthened.

Corrected. We changed the title to "Rossby wave-modulated orbital precipitation anomalies in the Asia-Pacific region" to soften the statement. We also provide new evidences for the relationship between the Rossby wave and other atmospheric circulation systems.

2. Abstract over-claims: "planetary wave propagation constitutes the primary determinant." This is not demonstrated quantitatively.

We have changed this sentence to "Our findings demonstrate that planetary wave propagation links together the IPWP atmospheric convection with the other atmospheric circulation system, which ultimately modulates the banding precipitation anomalies across the Asia-Pacific region.".

3. Figure 1 and proxy compilation, the band definition appears qualitative. A more objective classification would strengthen the identification of the hexapole pattern. Using only "representative records" from each band risks confirmation bias. The full dataset of 18 records should be analyzed statistically to determine whether banding exists independently of prior expectations.

We agree with your opinion. We have conducted filtering, precession correlation, correlation analysis and error assessment analysis on all the complied 18 paleo-precipitation records (Figure S1-4). The results showed that multiple records in the same Band exhibited basically the same phase differences compare the precession, although these records were based on independent chronologies and different proxies. In addition to the above theoretical uncertainty determination, the newly added Figures S1-4 enables us to have a more direct confirmation that this is a genuine difference in the precession phase of different Bands, rather than an error in the chronologies.

4. Phase relationships in Fig. 1b require statistical confidence intervals; tuning

uncertainties can reach several kyr and must be acknowledged explicitly.

Added. We have conducted filtering, precession correlation, correlation analysis and error assessment analysis on all the complied 18 paleo-precipitation records (Figure S1-4).

5. Interpretation of S/(I+C) as precipitation intensity requires clearer justification. Weathering is influenced by lithology, vegetation, and erosion processes—not only rainfall.

Corrected. Please refer to **Major Comment 6**.

6. EOF1 explains 86.5% of variance in 500 hPa geopotential height. This likely corresponds to large-scale monsoon intensity variations, or Hadley cell expansion/contraction, or shifts in seasonal thermal contrast. Rossby waves are patterns on top of mean-state circulation — not equivalent to EOF1. Interpreting EOF1 as a Rossby-wave diagnostic is not justified.

We agree with the reviewer that EOF1 of geopotential height does not, by itself, constitutes a Rossby-wave diagnostic and may primarily reflect large-scale background variability such as monsoon intensity, Hadley circulation strength, or land–sea thermal contrast. We therefore do not interpret EOF1 as direct evidence of Rossby waves.

In the revised manuscript, the EOF analysis is used only to demonstrate that the banded precipitation anomalies are dominated by coherent, large-scale circulation variability rather than by local processes. Specifically, the close correspondence between the leading EOF of summer precipitation anomalies (Figure S8b-8c) and the regressed 500 hPa circulation field (Figure 3) indicates strong dynamical control on the precipitation pattern.

Identification of Rossby-wave dynamics is not based on EOF analysis, but instead relies on independent dynamical diagnostics, including relative vorticity, RWS, WAF, and moisture budget analyses (Figures 4–5). These diagnostics explicitly resolve wave phase, source regions, propagation pathways, and quantitative contributions, and therefore provide the physical basis for interpreting the banded precipitation anomalies as a manifestation of stationary Rossby-wave adjustment for the summer mean circulation.

Thus, the revised manuscript now clearly distinguishes the **background circulation state** from the **Rossby wave perturbations**, offering a robust and complete physical explanation for the orbital-scale precipitation banding. Please see the new Figures 3-5 and lines 308-686.

7. The patterns in Figs. 2c–f (precipitation, geopotential height, vorticity) match known monsoon variability structures. They are not uniquely attributable to Rossby wave propagation. Moisture advection and vertical motion anomalies are not isolated.

We sincerely appreciate your insightful comment. We fully agree that monsoon dynamics is also the fundamental drivers shaping the spatial distribution of summer precipitation across the Asia-Pacific region.

In the revised manuscript, we therefore clarify that Rossby waves are not proposed as an alternative mechanism to the monsoon system. Instead, they are interpreted as a stationary dynamical response embedded within the summer mean monsoon circulation, acting to organize and redistribute moisture meridionally.

To address the reviewer's concern regarding attribution, we have explicitly isolated the circulation and moisture components associated with stationary wave activity:

(1) Because the analysis is based on seasonal-mean (JJA) precession-composited anomalies, transient synoptic eddies are largely filtered out, and the diagnosed anomalies represent stationary components. We further decompose precipitation anomalies using a vertically integrated moisture budget, separating dynamic moisture advection by anomalous winds $-\nabla \cdot (V'\overline{q})$ from thermodynamic effects $-\nabla \cdot (\overline{V}q')$ and vertical motion related convergence (Figure 5). This decomposition explicitly quantifies the contribution of wave-induced anomalous circulation to moisture transport, independent of the mean monsoon moisture supply.

(2) Independent diagnostics of relative vorticity, Rossby Wave Source, and wave activity flux (Figure 4) explicitly identify wave generation regions and meridional propagation pathways, which cannot be inferred from monsoon variability alone.

Together, these diagnostics demonstrate that while the monsoon establishes the background circulation and moisture supply, the superimposed stationary Rossby wave response provides the specific alternating wet–dry meridional structure observed in the six-band precipitation pattern.

We have revised the manuscript throughout to clearly distinguish the foundational role of the monsoon background from the modulating role of stationary Rossby waves. Please see the new Figures 4-5 and lines 384-686.

8. Wave activity flux arrows in Fig. 2g merely show that waves propagate under the resulting circulation anomaly. They do not demonstrate that waves are the source of the anomaly, or that waves are the mechanism mediating orbital-scale changes. The analysis is descriptive rather than diagnostic.

We fully agree with your comment and apologize for the original analysis being descriptive rather than diagnostic. To address this, we have supplemented the revised manuscript with wave source diagnostics and moisture budget analyses to establish a complete physical chain linking orbital forcing to wave generation, propagation, and precipitation response. Specifically, the revised manuscript includes the following new diagnostics:

(1) **Wave source identification**:

By calculating relative vorticity advection (RWS), we identified the wave source in the IPWP region (Figure 4, positive phase of IPWP).

(2) **Propagation pathway**:

Using wave activity flux (WAF) diagnostics, we trace the meridional propagation of

Rossby wave energy away from the diagnosed IPWP source region toward the subtropics and midlatitudes, establishing a physically consistent link between the tropical forcing and extratropical circulation response.

(3) **Moisture transport and convergence**:

We further diagnose vertically integrated moisture fluxes and convergence/divergence fields to track how the wave-induced circulation redistributes moisture and induces ascent and descent along the propagation pathway (Figure 5a).

(4) **Contribution Quantification**:

A vertically integrated moisture budget analysis decomposes precipitation anomalies into dynamic and thermodynamic components, showing that meridional dynamic moisture advection associated with stationary wave-induced circulation anomalies accounts for approximately 59–89% of the midlatitude precipitation anomalies (Figures 5b–c).

These diagnostics move the analysis beyond a descriptive interpretation of wave patterns and demonstrate that Rossby waves act as a dynamical mediator which transports low-latitude moisture to mid-latitudes and regulates orbital-scale precipitation anomalies. We have added the above information in the revised manuscript. Please see the new Figures 3-5 and lines 308-686.

9. Sections on land–sea contrast dismisses its role too quickly; these mechanisms remain well-established drivers in the literature.

We agree with the reviewer that land–sea thermal contrast is a well-established and fundamental driver of large-scale monsoon circulation and summer precipitation, and we did not intend to downplay its role. In the revised manuscript, we have clarified this point and revised the relevant sections to explicitly acknowledge land–sea contrast as a first-order control on the background mean state of regional precipitation.

Our interpretation of Rossby waves is not meant to replace or compete with land–sea contrast. Rather, Rossby waves are treated as a dynamical adjustment and organizing process embedded within the mean circulation established by land–sea thermal contrast, monsoon dynamics, and ITCZ migration. Within this framework, land–sea contrast provides the primary thermal forcing that sets up the background circulation, while Rossby waves regulate how this forcing is communicated meridionally and expressed as a coherent banded precipitation structure observed in our results.

We have revised the manuscript accordingly to better reflect this hierarchy of mechanisms and to align our interpretation with the established literature on land–sea contrast–driven monsoon variability. Please see lines 611-635.

10. For the seasonality, good that authors acknowledge winter vs summer differences. But there is no mechanistic diagnosis of how seasonal mean changes shift stationary wave characteristics.

We thank the reviewer for this important point. In the revised manuscript, we explicitly diagnose how seasonal-mean circulation changes regulate stationary Rossby-wave

characteristics. We have added relative vorticity, RWS and WAF diagnostics for boreal winter in the transient CESM simulation (new Figures S12–S13), together with an expanded discussion in the Seasonal Sensitivity section. Please see lines 636-686.

The diagnostics show that, in winter, positive RWS anomalies are weak near ~30°N, in contrast to the coherent tropical source in summer. Consistently, WAF vectors indicate predominantly zonal wave propagation in the midlatitude, reflecting a winter mean-state waveguide that suppresses meridional energy propagation there. In addition, weak low-level (850 hPa) relative vorticity anomalies and reduced moisture transport inhibit the downward coupling of upper-level wave signals to surface precipitation.

As a result, although midlatitude Rossby-wave activity is present in winter, the altered seasonal mean state prevents effective meridional wave adjustment and moisture redistribution, leading to a weakened or absent latitudinal precipitation banding. These new diagnostics provide a mechanistic explanation for how seasonal-mean circulation changes modulate Rossby wave structure and its hydrological impacts.

11. Conceptual figure 4 is plausible but schematic and not quantitatively demonstrated. If Rossby waves are key, authors should show anomalous heating centers, Rossby source function, and stationary wavenumber fields.

We have comprehensively revised this conceptual Figure to directly integrate quantitative dynamical diagnostics. The new Figure 6 now overlays anomalous diabatic heating associated with IPWP deep convection, the RWS, and WAF, thereby quantitatively demonstrating how anomalous convection excites Rossby wave and how their energy propagates meridionally to redistribute moisture and modulate large-scale precipitation patterns. The conceptual framework is thus directly supported by physically quantitative dynamical diagnostics, rather than schematic illustration alone.

In response to the suggestion regarding stationary wavenumber, we additionally provide the meridional scale of the Rossby-wave response at 200 hPa associated with the dominant precession (23 kyr) variability (Figure S10). This result indicates a low-order meridional response, with characteristic spatial scales broadly comparable to meridional wavelengths of ~20°–60° latitude, consistent with planetary-scale stationary Rossby waves.

We emphasize, however, that the hexapole precipitation banding examined here is a regional, zonally inhomogeneous feature confined to the Asia–Pacific sector, with non-uniform spacing between anomaly centers imposed by the background monsoon circulation and mid-latitude westerly jet. These characteristics limit the applicability of formal hemispheric Fourier decomposition or wavenumber decompositions. Accordingly, the meridional scale estimate here is presented as a qualitative support, while the primary physical evidence for a Rossby wave mechanism is provided by the diagnosed relative vorticity, RWS, WAF, vertically-integrate moisture flux and budget analyses.

We therefore focus on diagnostics that are physically appropriate for a zonally asymmetric, regionally confined Rossby wave response, rather than on idealized wavenumber decompositions.

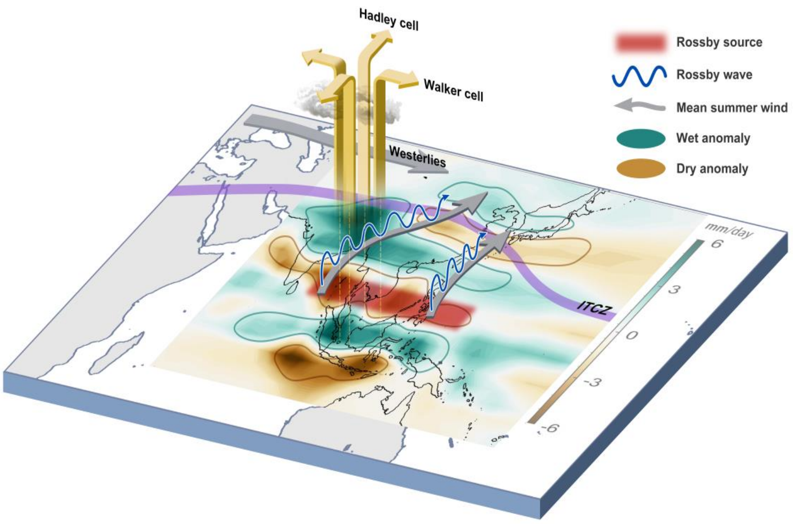

Figure 6 Schematic diagram of precipitation banding anomaly formation in the Asia-Pacific region. Precession-regulated insolation alters deep convection intensity (grey clouds) over the IPWP, generating a Rossby wave source (red shading) and upper-tropospheric (200 hPa) divergence (yellow arrows). The resulting meridionally propagating Rossby waves (blue curves) and moisture transport along the South Asian and western Pacific pathways (grey arrows) bridge tropical forcing to extratropical circulation, organizing coherent precipitation banding anomalies

12. Wave activity flux formula is standard, but use of only 200 hPa level may miss low- to mid-tropospheric interactions. Insufficient detail on seasonal filtering and frequency filtering. No moisture budget diagnostics are performed.

We agree with your viewpoint. We have made following corrections:

(1) We have added the wave activity flux (WAF) analysis results for the 500 hPa and 850 hPa pressure levels (Figure 4). The results show consistent meridional propagation and circulation signatures across the troposphere, indicating that the diagnosed wave response is not confined to a single upper-level layer.

(2) Using the Lanczos filtering method (Duchon, 1979), we extracted the 23-kyr period signal, explicitly labeled the season selection (JJA or DJF).

(3) We also added vertically integrated moisture flux, divergence, and budget diagnostic analyses (Figure 5), decomposing precipitation anomalies into dynamic and thermodynamic contributions. These diagnostics demonstrate that circulation-induced moisture advection dominates the precipitation response, quantitatively linking the Rossby wave induced circulation anomalies to the banded precipitation structure.

The above analyses demonstrate that the banded precipitation anomalies are not a local feature, but a robust response of the precipitation field to large-scale atmospheric circulation changes. Rossby waves act as an important organizing and redistributive process, mediating meridional moisture transport under a precession-modulated summer mean state. The consistency of this mechanism across orbital-scale composites, pre-industrial simulations, and the modern PJ pattern further supports its robustness across different climatic backgrounds. Please see the new Figures 4-5 and lines 384-686.

References:

Aldrian, E., Susanto, R., 2003. Identification of three dominant rainfall regions within Indonesia and their relationship to sea surface temperature. International Journal of Climatology: A Journal of the Royal Meteorological Society 23(12), 1435–1452.

Beck, J.W., Zhou, W., Li, C., Wu, Z., White, L., Xian, F., Kong, X., An, Z., 2018. A 550,000-year record of East Asian monsoon rainfall from 10Be in loess. science 360(6391), 877–881.

Bereiter, B., Eggleston, S., Schmitt, J., Nehrbass-Ahles, C., Stocker, T.F., Fischer, H., Kipfstuhl, S., Chappellaz, J., 2015. Revision of the EPICA Dome C CO2 record from 800 to 600 kyr before present. Geophysical Research Letters 42(2), 542–549.

Berger, A., 1978. Long-term variations of daily insolation and Quaternary climatic changes. Journal of the Atmospheric Sciences 35(12), 2362–2367.

Chamley, 1989. Clay Sedimentology. Springer.

Cheng, H., Edwards, R.L., Sinha, A., Spötl, C., Yi, L., Chen, S., Kelly, M., Kathayat, G., Wang, X., Li, X., Kong, X., Wang, Y., Ning, Y., Zhang , H., 2016. The Asian monsoon over the past 640,000 years and ice age terminations. Nature 534(7609), 640–646.

Cheng, H., Li, H., Sha, L., Sinha, A., Shi, Z., Yin, Q., Lu, Z., Zhao, D., Cai, Y., Hu, Y., Hao, Q., Tian, J., Kathayat, G., Dong, X., Zhao, J., Zhang, H., 2022. Milankovitch theory and monsoon. The Innovation 3(6). doi.org/10.1016/j.xinn.2022.100338.

Clemens, S.C., Murray, D.W., Prell, W.L., 1996. Nonstationary Phase of the Plio-Pleistocene Asian Monsoon. Science 274(5289), 943.

Duchon, C.E., 1979. Lanczos filtering in one and two dimensions. Journal of Applied Meteorology (1962-1982), 1016–1022.

Heaton, T.J., Köhler, P., Butzin, M., Bard, E., Reimer, R.W., Austin, W.E., Ramsey, C.B., Grootes, P.M., Hughen, K.A., Kromer, B., 2020. Marine20—the marine radiocarbon age calibration curve (0–55,000 cal BP). Radiocarbon 62(4), 779–820.

Hu, P., Chen, W., Chen, S., Yang, R., Wang, L., Liu, Y., 2024. Revisiting the Linkage Between the Pacific–Japan Pattern and Indian Summer Monsoon Rainfall: The Crucial Role of the Maritime Continent. Geophysical Research Letters 51(3), e2023GL106982. doi.org/https://doi.org/10.1029/2023GL106982.

Huyghe, P., Guilbaud, R., Bernet, M., Galy, A., Gajurel, A.P., 2011. Significance of the clay mineral distribution in fluvial sediments of the Neogene to Recent Himalayan Foreland Basin (west-central Nepal). Basin Research 23(3), 332–345.

Jian, Z., Wang, Y., Dang, H., Mohtadi, M., Rosenthal, Y., Lea, D.W., Liu, Z., Jin, H., Ye, L., Kuhnt, W., 2022. Warm pool ocean heat content regulates ocean–continent moisture transport. Nature 612(7938), 92–99.

Kissel, C., Laj, C., Kienast, M., Bolliet, T., Holbourn, A., Hill, P., Kuhnt, W., Braconnot, P., 2010. Monsoon variability and deep oceanic circulation in the western equatorial Pacific over the last climatic cycle: Insights from sedimentary magnetic properties and sortable silt. Paleoceanography 25(3).

Kosaka, Y., Nakamura, H., 2010. Mechanisms of meridional teleconnection observed between a summer monsoon system and a subtropical anticyclone. Part I: The Pacific–Japan pattern. Journal of Climate 23(19), 5085–5108.

Laskar, J., Robutel, P., Joutel, F., Gastineau, M., Correia, A., Levrard, B., 2004. A long-term numerical solution for the insolation quantities of the Earth. Astronomy & Astrophysics 428(1), 261–285.

Lisiecki, L., Raymo, M., 2005. A Pliocene-Pleistocene stack of 57 globally distributed benthic $\delta^{18}O$ records. Paleoceanography 20, PA1003.

Wang, B., Liu, J., Kim, H.-J., Webster, P.J., Yim, S.-Y., 2012. Recent change of the global monsoon precipitation (1979–2008). Climate Dynamics 39(5), 1123–1135. doi.org/10.1007/s00382-011-1266-z.

West, A., Galy, A., Bickle, M., 2005. Tectonic and climatic controls on silicate weathering. Earth and Planetary Science Letters 235(1-2), 211–228. doi.org/https://doi.org/10.1016/j.epsl.2005.03.020.

Willeit, M., Ganopolski, A., Calov, R., Brovkin, V., 2019. Mid-Pleistocene transition in glacial cycles explained by declining CO₂ and regolith removal. Science Advances 5(4), eaav7337. doi.org/doi:10.1126/sciadv.aav7337.

Wu, J., Liu, Z., Zhou, C., 2012. Late quaternary glacial cycle and precessional period of clay mineral assemblages in the western pacific warm pool. Chinese Science Bulletin 57(28), 3748–3760. doi.org/10.1007/s11434-012-5277-x.

Xie, P., Arkin, P., 1997. Monitoring large-scale precipitation over the globe.

Xie, S.-P., Kosaka, Y., Du, Y., Hu, K., Chowdary, J.S., Huang, G., 2016. Indo-western Pacific ocean capacitor and coherent climate anomalies in post-ENSO summer: A review. Advances in Atmospheric Sciences 33(4), 411–432. doi.org/10.1007/s00376-015-5192-6.

Yaglom, A.M., 2004. An introduction to the theory of stationary random functions. Courier Corporation.

Yamamoto, M., Ichikawa, Y., Igarashi, Y., Oba, T., 2005. Late Quaternary variation of lignin composition in core MD01-2421 off central Japan, NW Pacific. Palaeogeography, Palaeoclimatology, Palaeoecology 229(3), 179–186. doi.org/https://doi.org/10.1016/j.palaeo.2005.06.021.

Yu, Z., Ruan, J., Song, L., Yun, K.-S., Tang, X., Colin, C., Wilson, D.J., Dang, H., DiNezio, P.N., Chang, F., 2024. Late Pleistocene island weathering and precipitation in the Western Pacific Warm Pool. npj Climate and Atmospheric Science 7(1), 91.

Yu, Z., Tang, X., Colin, C., Wilson, D.J., Zhou, X., Song, L., Chang, F., Zhang, S., Bassinot, F., Wan, S., 2023. Millennial-scale precipitation variability in the Indo-Pacific region over the last 40 kyr. Geophysical Research Letters n/a(n/a), e2022GL101646. doi.org/https://doi.org/10.1029/2022GL101646.

Yun, K.S., Timmermann, A., Lee, S.S., Willeit, M., Ganopolski, A., Jadhav, J., 2023. A transient coupled general circulation model (CGCM) simulation of the past 3 million years. Clim. Past 19(10), 1951–1974. doi.org/10.5194/cp-19-1951-2023.

Zhang, H., Griffiths, M.L., Chiang, J.C., Kong, W., Wu, S., Atwood, A., Huang, J., Cheng, H., Ning, Y., Xie, S., 2018. East Asian hydroclimate modulated by the position of the westerlies during Termination I. Science 362(6414), 580–583.

Response to Comments

Reviewer #3:

The authors have made a serious and substantive effort to address the major concerns. Compared to the original submission, the revised manuscript is much stronger, especially in clarifying that Rossby waves are not acting on orbital timescales, but as seasonally recurrent stationary responses. They also added Rossby wave source (RWS), wave activity flux (WAF), and moisture budget diagnostics. The Methods section now is expanded to describe forcing, acceleration, and spin-up. Following the comments, they explicitly tested alternative mechanisms (ITCZ, Hadley, WPSH, SAM), and softened some of the more problematic causal language.

Response: We sincerely thank you for the insightful feedback, which has significantly improved the quality and clarity of our work. We are glad that our revisions, especially the clarification that Rossby waves act as seasonally recurrent stationary responses rather than orbital-scale processes, have been recognized by you. Below we respond to your main concerns and clarify our interpretations based on additional reasoning and evidence.

Several non-trivial issues remain and should be resolved before the manuscript is accepted for publication. See below.

1. The expanded Methods section is an improvement, but it raises new concerns that are not discussed critically enough. (1) Ice-sheet geometry and topography are prescribed from CLIMBER-2 and remapped every 100 years, but freshwater fluxes from ice sheets are explicitly excluded, despite their known influence on large-scale circulation. The justification ("to avoid underestimating AMOC variability") is not convincing without sensitivity testing. (2) The land–sea mask is fixed at LGM conditions for the entire 800 kyr simulation. This is a very strong assumption for a study claiming to diagnose pan-Asian hydroclimate mechanisms. The implications for monsoon pathways, coastlines, and moisture transport are not discussed. I understand it won't be possible to redo the simulation, but the authors need to provide a clear discussion of the limitations introduced by fixed LGM coastlines, absence of meltwater forcing, and use of a reduced-complexity ice-sheet product (CLIMBER-2). At minimum, the authors should explicitly state how these choices might bias Rossby wave propagation, moisture pathways, and band spacing and amplitude. At present, the Methods describe what was done, but not whether it matters for the conclusions.

Response: We agree with you that the experimental design involves several idealizations, including (i) the absence of freshwater forcing from ice sheets, (ii) the use of a fixed LGM land–sea configuration, and (iii) the prescribed ice-sheet geometry from a reduced-complexity model. These simplifications may influence the simulated climate system in several ways:

(1) Freshwater forcing can affect ocean circulation, especially the variability of the

Atlantic Meridional Overturning Circulation (AMOC) (e.g., Rahmstorf, 2002; Stouffer et al., 2006), thereby modulating the amplitude of climate variability.

(2) The fixed land–sea mask may alter regional moisture transport pathways and the surface heating distributions, potentially affecting local precipitation intensity.

(3) The prescribed ice-sheet geometry may influence the background atmospheric circulation through large-scale forcing (e.g., Cook and Held, 1988; Roe and Lindzen, 2001), but with little sensitivity to small-scale ice-sheet details (e.g., Liakka and Nilsson, 2010).

We emphasize, however, that these factors are expected to primarily influence the amplitude and regional expression of climate anomalies, rather than the large-scale dynamical mechanism that is the focus of this study. The mechanism we propose is fundamentally atmospheric and involves Rossby wave excitation by tropical heating over the Indo-Pacific Warm Pool, followed by propagation along the large-scale circulation waveguide. These processes are governed by large-scale circulation structure and planetary wave dynamics, which are not fundamentally altered by the absence of meltwater forcing or by regional coastline details.

Consequently, while regional precipitation intensity, band positioning, and local moisture convergence may be affected, the large-scale linkage among tropical convection, Rossby wave propagation, and meridional moisture transport is expected to remain robust.

We have revised the manuscript to explicitly clarify these limitations and their potential impacts, and to avoid overinterpretation of regional-scale details. Accordingly, our conclusions focus on the robustness of the large-scale dynamical mechanism, while regional features are interpreted with appropriate caution.

Please see Lines 628-637: "*Several limitations of the experimental design should be considered. The absence of freshwater forcing from ice sheets may affect AMOC variability (Rahmstorf 2002; Stouffer., et al. 2006), while the use of a fixed LGM land–sea mask and prescribed ice-sheet geometry may influence regional moisture pathways and precipitation intensity (Cook and Held 1988; Roe and Lindzen 2011). However, these factors primarily affect the amplitude and regional expression of precipitation anomalies. The large-scale atmospheric circulation and planetary wave responses identified in this study are mainly controlled by tropical heating and background circulation, and are therefore expected to be robust. Consequently, these limitations do not qualitatively alter the dynamical mechanism proposed here, although regional details should be interpreted with caution.*"

References:

Cook, K. H., & Held, I. M. (1988). Stationary waves of the ice age climate. Journal of Climate, 1(8), 807–819.

Roe, G. H., & Lindzen, R. S. (2001). The mutual interaction between continental-scale ice sheets and atmospheric stationary waves. Journal of Climate, 14(7), 1450–1465.

Liakka, J., & Nilsson, J. (2010). The impact of topographically forced stationary waves on local ice-sheet climate. Journal of Glaciology, 56(199), 534–544.

Stouffer, R. J., et al. (2006), Investigating the causes of the response of the thermohaline circulation to past and future climate changes, Journal of Climate, 19, 1365–1387.

Rahmstorf, S. (2002), Ocean circulation and climate during the past 120,000 years, Nature, 419, 207–214.

2. The added PI control and PJ-pattern comparison (Figure S7) is helpful, but validation is still incomplete. The PI control uses LGM land–sea mask, not modern geography. Validation is largely limited a to spatial similarity of the PJ pattern and qualitative agreement with CMAP precipitation. There is no validation of mean monsoon rainfall magnitude, seasonal cycle realism, vertical circulation structure, jet position and strength, moisture transport strength. Because the central claim depends on subtle circulation–moisture interactions, even moderate biases in monsoon strength, subtropical jets, and background shear, could strongly affect the diagnosed Rossby-wave pathways. The manuscript still lacks a concise validation statement explaining what the model does well, what it does not, and why this does not invalidate the conclusions.

Response: Thank you for this insightful comment and for emphasizing the importance of comprehensive model validation. In response, we have substantially strengthened the evaluation of the PI control simulation by explicitly assessing the realism of precipitation, circulation, and moisture fields that are directly relevant to the proposed mechanism (Figures S8-9, S19).

The model reproduces the large-scale spatial distribution of boreal summer (JJA) monsoon precipitation and the overall spatial organization of tropical convection over the Indo-Pacific region, consistent with CMAP observations, although some regional biases exist (Figure S8a–b). The model also faithfully reproduces the seasonal cycle of monsoon circulation and precipitation (Figure S9).

In the upper troposphere, the simulated 200 hPa subtropical jet exhibits realistic latitude (~35–40°N) and strength (~30–35 m s^{-1}) comparable to ERA5, with a modest poleward shift (Figure 8Sc–d). At lower levels (850 hPa; Figure 8Se–f), the model captures the observed southwesterly monsoon over the Indian Ocean and the western North Pacific, and, in combination with the upper-level easterlies, reproduces the observed vertical wind structure.

Importantly, vertically integrated moisture transport magnitudes and pathways are broadly consistent with ERA5 reanalysis (Figure S19), supporting a physically consistent representation of moisture supply to tropical convection and meridional Rossby propagation.

Our analysis focuses on large-scale dynamical structures: subtropical jet, background circulation, and meridional moisture transport, all of which are important to Rossby wave propagation and teleconnection dynamics. Although the PI experiment uses an

LGM rather than modern land–sea mask and contains modest mean rainfall biases, these do not alter the qualitative dynamical relationships or Rossby wave pathways central to our conclusions.

We have added ERA5 analysis data and the following new figures, revised the manuscript to clarify these points and moderated overly strong statements accordingly.

Please see Lines 180-184: "*The PI control also realistically reproduces the large-scale boreal summer monsoon circulation and precipitation: IPWP rainfall pattern agrees well with CMAP observations (Figure S8), the 200 hPa subtropical jet (35–40°N, ~30–35 m/s), 850 hPa southwesterly monsoon flow, and the monsoon seasonal cycle are also well represented (Figure S9), despite modest regional amplitude biases. These results further confirm the model's capability to realistically simulate the band-shaped precipitation anomaly*"
Lines 506-510: "*The model used in this study realistically captures large-scale features (subtropical jet, seasonal cycle of monsoon, moisture transport). Regional precipitation biases and LGM boundary conditions may affect local details, but not the large-scale coupling of tropical heating, Rossby wave propagation, and meridional moisture transport.*"
Lines 623-627: "*Vertically integrated moisture transport magnitude and pathways are broadly consistent with ERA5 (Figure S19). While modest biases exist and an LGM land–sea mask is employed, the large-scale dynamical features critical to Rossby-wave propagation and moisture–circulation interactions are faithfully captured.*"

Line 587-590: "*Atmospheric fields (winds, humidity, geopotential height) are from ERA5 reanalysis (Hersbach et al., 2020), provided by ECMWF at 0.25° resolution with 37 pressure levels. ERA5 is employed to diagnose circulation, moisture transport, and Rossby wave dynamics, owing to its high accuracy and dynamical consistency.*"

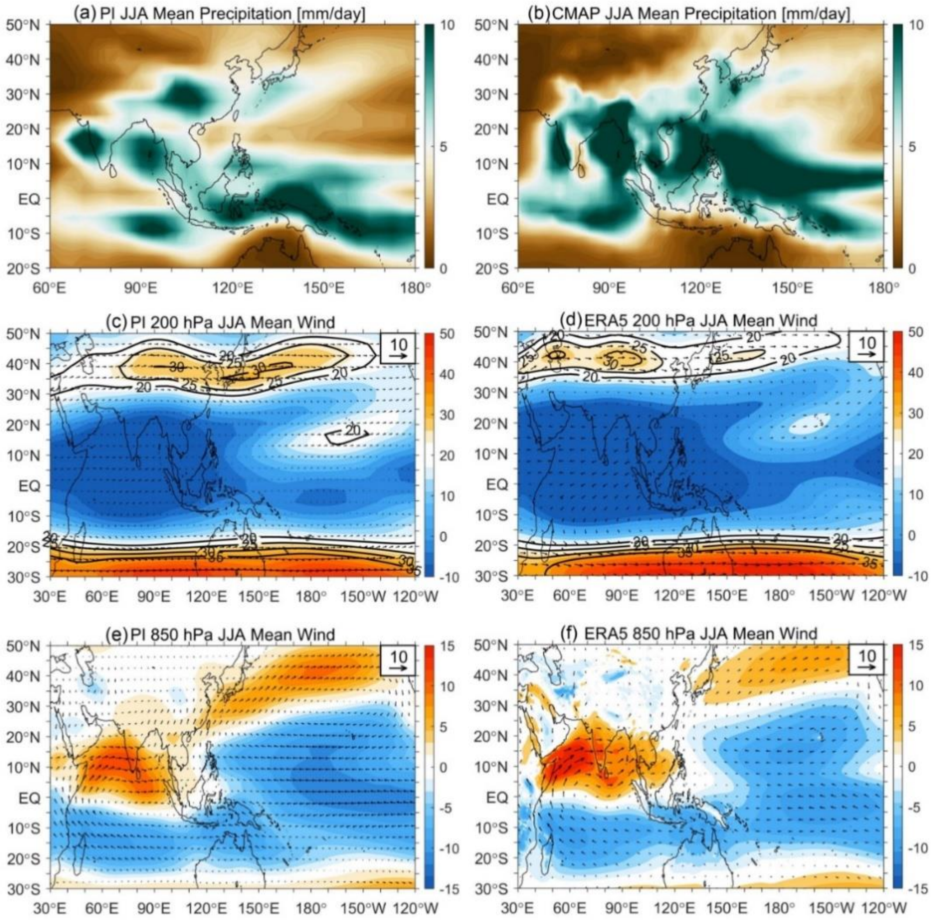

Figure S8 Validation of monsoon precipitation and circulation in the PI control simulation. JJA mean precipitation (units: mm/day) in the (a) PI control simulation and (b) CMAP observations. (c) JJA mean horizontal winds at 200 hPa in the PI control (shadings: zonal current; units: m/s; contours denote zonal wind with intervals of 5 m/s) and (d) ERA5 reanalysis. (e-f) are the same as (c-d) but at 850 hPa.

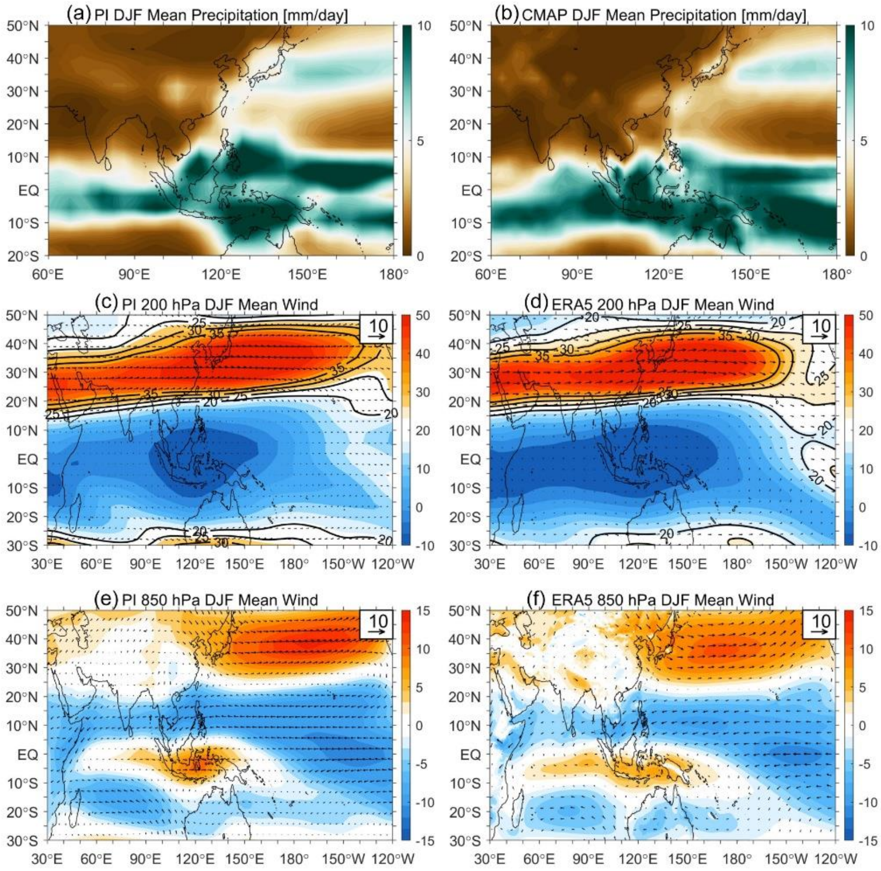

Figure S9 The same as Figure S8, but for winter.

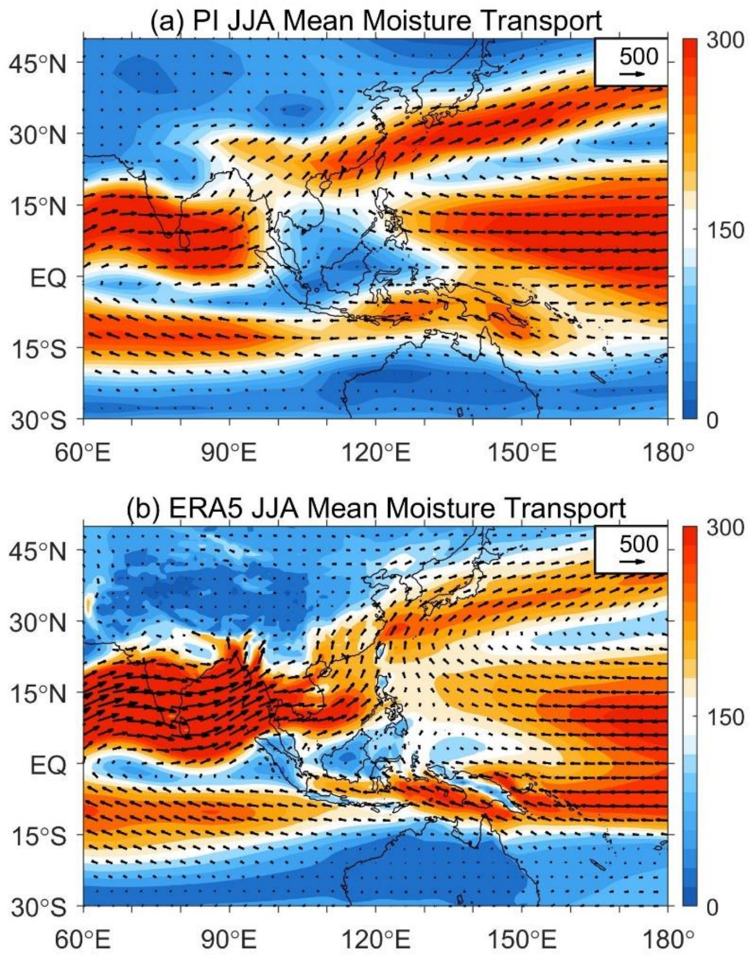

Figure S19 Validation of monsoon moisture transport in the PI control simulation. JJA mean vertically integrated moisture transport (vectors) and its magnitude (shadings; units: kg/m/s) in the (a) PI control and (b) ERA5.

3. The authors now present a coherent diagnostic chain: IPWP convection → RWS → WAF → moisture advection → precipitation bands. This is a major improvement. However, all diagnostics are still regression-based and internally consistent, but not causal. No new sensitivity experiments are actually performed in this study. The argument relies heavily on analogy with the modern PJ pattern and previously published sensitivity experiments (Hu et al., 2024). The authors need to provide at least one of the following: a simple idealised perturbation within this study (e.g. suppressed IPWP heating), or a quantitative comparison showing that removing Rossby-wave-related circulation terms collapses the banding pattern. Without this, the Rossby-wave mechanism remains well-supported but not uniquely demonstrated.

Response: We thank the reviewer for this insightful and constructive comment. We agree that our original regression-based diagnostics, while internally consistent, did not fully establish the causal and essential role of Rossby wave dynamics, rather than mere statistical correlation.

To address this point, we have added a strong-weak composite analysis based on an objective measure of upper-tropospheric Rossby-wave activity (Figure S13). We construct a projection index by projecting the anomalous geopotential height onto a reference Rossby wave pattern, following Wallace and Gutzler (1981) and Barnston and Livezey (1987). The reference pattern is defined as the regression of 200 hPa geopotential height onto the precession index (the dominant wave mode linked to IPWP convection). Projections are performed over the subtropical waveguide region (15°–50°N, 60°–180°E), with cosine latitude weighting:

$$I(t) = \frac{\sum_{x,y} Z'(x,y,t) Z_{ref}(x,y) \cos\phi}{\sum_{x,y} Z_{ref}(x,y)^2 \cos\phi}$$

where Z' is the geopotential height anomaly, Z_{ref} is the reference pattern, and ϕ is latitude. The index is normalized by its standard deviation. This index provides a measure of the amplitude of the associated wave response. Strong and weak Rossby wave phases are defined as periods when the normalized index exceeds ±1 standard deviation and lies within ±0.5 standard deviation, respectively.

During strong Rossby wave phases, a well-organized Rossby wave source (RWS) and coherent wave activity flux (WAF) propagation are evident along the subtropical waveguide, accompanied by pronounced banded precipitation anomalies across pan-Asia (Figure S13). In contrast, during weak phases, the wave structure and WAF propagation are largely weakened and lose their spatial coherence, and the banded precipitation structure fully collapses.

Quantitative analysis further confirms that precipitation anomalies within each precipitation band are strong and spatially coherent during strong Rossby-wave phases, but weak and insignificant during weak phases (Figure S13g). The contrast effectively serves as a dynamical "with vs. without" comparison, showing that reducing (or removing) Rossby wave activity leads to a collapse of the banded precipitation structure, which rigorously validates the unique and necessary role of Rossby wave propagation in organizing the observed precipitation bands.

We have incorporated these new analyses and figures into the revised manuscript and supplementary file, respectively.

Please see Lines 360-369: "*During strong Rossby wave phases, pronounced WAF propagation along the subtropical waveguide accompanies clear, coherent banded precipitation anomalies across pan-Asia (Figure S13). During weak phases, the wave structure and organized precipitation bands are largely suppressed. Quantitatively, each band precipitation anomalies are strong and spatially coherent under active Rossby wave conditions but weak and insignificant during weak periods. This strong–weak phase contrast further demonstrates that reducing (or removing) Rossby wave activity leads to a collapse of the banded precipitation structure, which rigorously validates the essential role of Rossby wave propagation in organizing the observed precipitation bands*."

Lines 698-710: “***Projection of Rossby-wave activity***

To quantify the amplitude of the upper-tropospheric Rossby wave pattern, we construct a projection index by projecting the anomalous geopotential height onto a reference Rossby wave pattern, following Wallace and Gutzler (1981) and Barnston and Livezey (1987). The reference pattern is defined as the regression of 200 hPa geopotential height onto the precession index (the dominant wave mode linked to IPWP convection). Projections are performed over the subtropical waveguide region (15°–50°N, 60°–180°E), with cosine latitude weighting:

$$I(t) = \frac{\sum_{x,y} Z\prime(x,y,t) Z_{ref}(x,y) \cos\phi \emptyset}{\sum_{x,y} Z_{ref}(x,y)^2 \cos\phi} \qquad (5)$$

where Z' *is the geopotential height anomaly,* Z_{ref} *is the reference pattern, and* ϕ *is latitude. The index is normalized by its standard deviation. This index provides a measure of the amplitude of the associated wave response. Strong and weak Rossby wave phases are defined as periods when the normalized index exceeds ±1 standard deviation and lies within ±0.5 standard deviation, respectively.*”

References:

Wallace, J. M., and D. S. Gutzler, 1981: Teleconnections in the geopotential height field during the Northern Hemisphere winter. Monthly Weather Review, 109, 784–812.

Barnston, A. G., and R. E. Livezey, 1987: Classification, seasonality and persistence of low-frequency atmospheric circulation patterns. Monthly Weather Review, 115, 1083–1126.

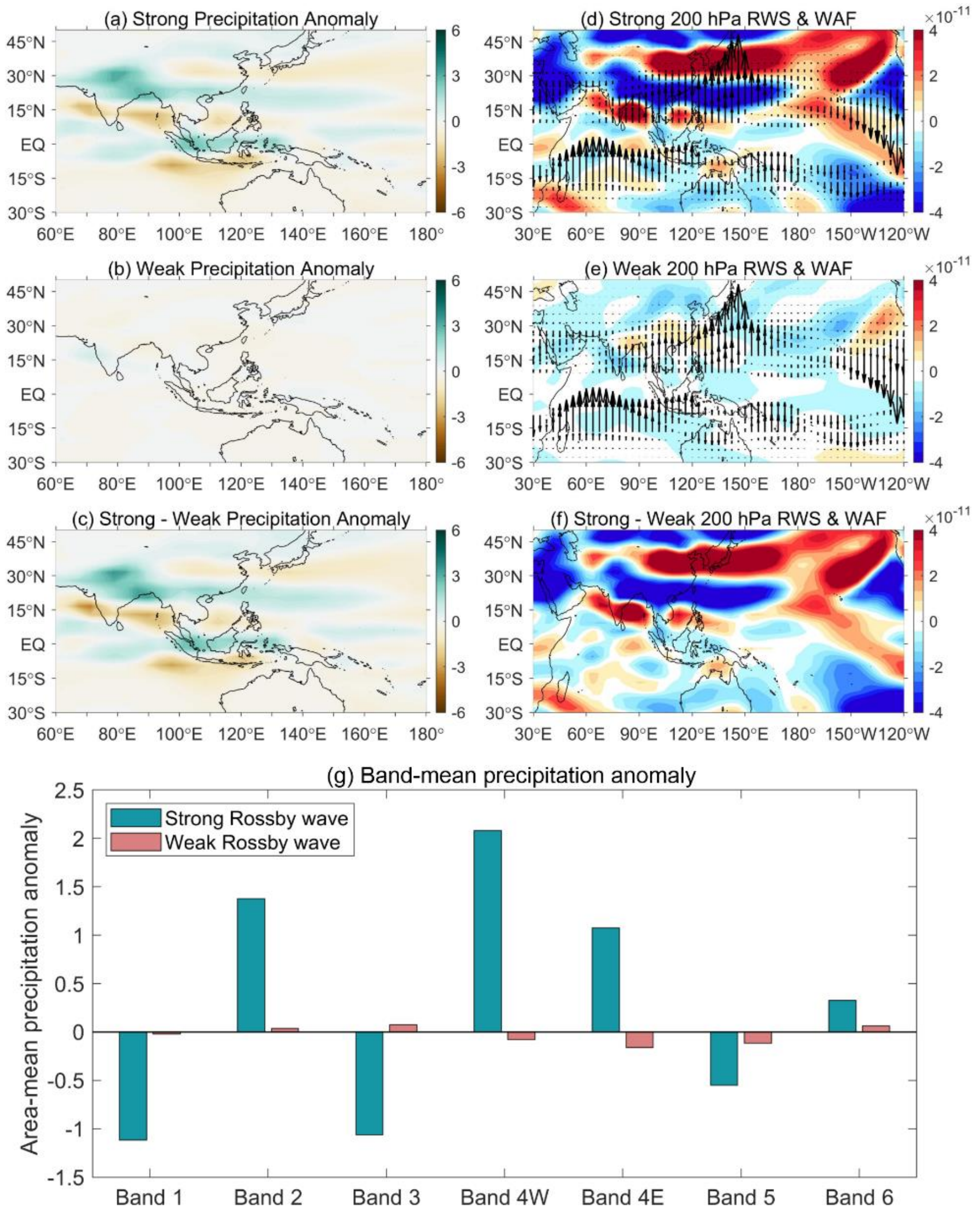

Figure S13 Rossby-wave modulation of banded precipitation. Precipitation anomaly during (a) strong Rossby-wave phases, (b) weak Rossby-wave phases and (c) difference between strong and weak phases (strong minus weak), respectively. The same as (a-c) but for Rossby wave source (RWS; shading) and wave activity flux (WAF; vectors) at 200 hPa. (g) Area-mean precipitation anomalies averaged over the predefined bands (Bands 1–6) for strong (green) and weak (red) Rossby-wave phases.

4. The authors now argue that bands emerge from EOFs, boundaries are not sharp, and the pattern is a low-order meridional mode. This is reasonable, but EOFs are not equivalent to physical modes, and boundaries are still defined post hoc for budget analysis, the six-band structure is not shown to be statistically optimal, robust to domain choice, and stable across different filtering choices. A brief sensitivity test by changing

latitude bins, or showing that the moisture-budget dominance does not depend on exact band definition, would be helpful. Without this, skepticism about "hexapole specificity" may remain.

Response: We thank the reviewer for raising the important concern regarding the physical interpretation of the banded structure and its sensitivity to methodological choices. We address this issue by focusing on dynamical consistency and robustness to alternative band definition.

The banded precipitation pattern is not defined a priori, but it instead guided by the observed large-scale meridional structure of precipitation anomalies, with each band chosen to roughly enclose a coherent anomaly center within the alternating meridional pattern. While EOFs are not strict physical modes, their high spatial coherence and dynamical consistency supports a physically meaningful interpretation. As shown in Response to Comment 3, this mode is dynamically linked to atmospheric Rossby waves forced by tropical convection.

To evaluate robustness to band definition, **we perform two sensitivity tests**: (i) shifting all bands meridionally by ±3.75° (one grid spacing), and (ii) applying 12° meridional smoothing (Figures S14-15). The large-scale alternating banded structure is qualitatively in both tests, and the overall moisture budget balance remains consistent across all configurations. In particular, the meridional moisture advection term ($-v^{'}\overline{q}$) maintains a close relationship with precipitation anomalies, indicating that **the primary dynamical interpretation is not sensitive to reasonable variations in band definition**.

While area-mean amplitudes in Bands 5-6 exhibit sensitivity to latitudinal shifts (Figure S15), this behavior has a clear physical basis: these bands lie within regions of strong meridional gradients of alternating-signed precipitation anomalies. Southward shifts increase the contribution of opposite-signed anomalies from adjacent bands, causing partial cancellation and reduced area means. Importantly, precipitation and the $-v'\overline{q}$ term weaken simultaneously, indicating the sensitivity reflects spatial sampling of a continuous structure, not a failure of the proposed dynamical mechanism.

The purpose of these sensitivity tests is to confirm stability of the large-scale structure and dynamical linkage, not local amplitude invariance. Sensitivity near band edges further supports that the precipitation pattern reflects a continuous meridional structure, rather than artificially discrete zones.

Together, these results demonstrate that tropical convection excites Rossby waves, whose propagation organize meridional moisture transport, forming a robust, large-scale banded precipitation pattern with consistent dynamical signatures. The six-band structure thus represents a convenient discretization of an underlying continuous mode, and our conclusions do not depend on arbitrary band definition.

We have added the following content and figures in the revised manuscript and supplementary file.

Please see Lines 370-385: "*The banded precipitation pattern is not defined a priori, but each band is chosen to roughly enclose a coherent anomaly center within the alternating meridional pattern. To evaluate robustness to band definition, we perform two sensitivity tests: (i) shifting all bands meridionally by ±3.75° (one grid spacing), and (ii) applying 12° meridional smoothing (Figures S14-15). The close correspondence between meridional moisture advection ($-v'\bar{q}$) and precipitation anomalies is preserved across both tests, indicating the core dynamical linkage is insensitive to reasonable variations in band definition. Area-mean amplitude sensitivity in Bands 5–6 arises from their location within strong meridional anomaly gradients, where small shifts cause partial cancellation of opposite-signed signals (Figure S15c and e). The simultaneous weakening of precipitation and $-v'\bar{q}$ reflects spatial sampling of a continuous structure, not a breakdown of the underlying mechanism. These results demonstrate the banded pattern is a robust, large-scale dynamical structure driven by Rossby wave induced moisture transport, not an artifact of band definition. The six-band configuration is thus a convenient discretization of a continuous meridional mode, and our dynamical conclusions are independent of exact band boundary choices.*"

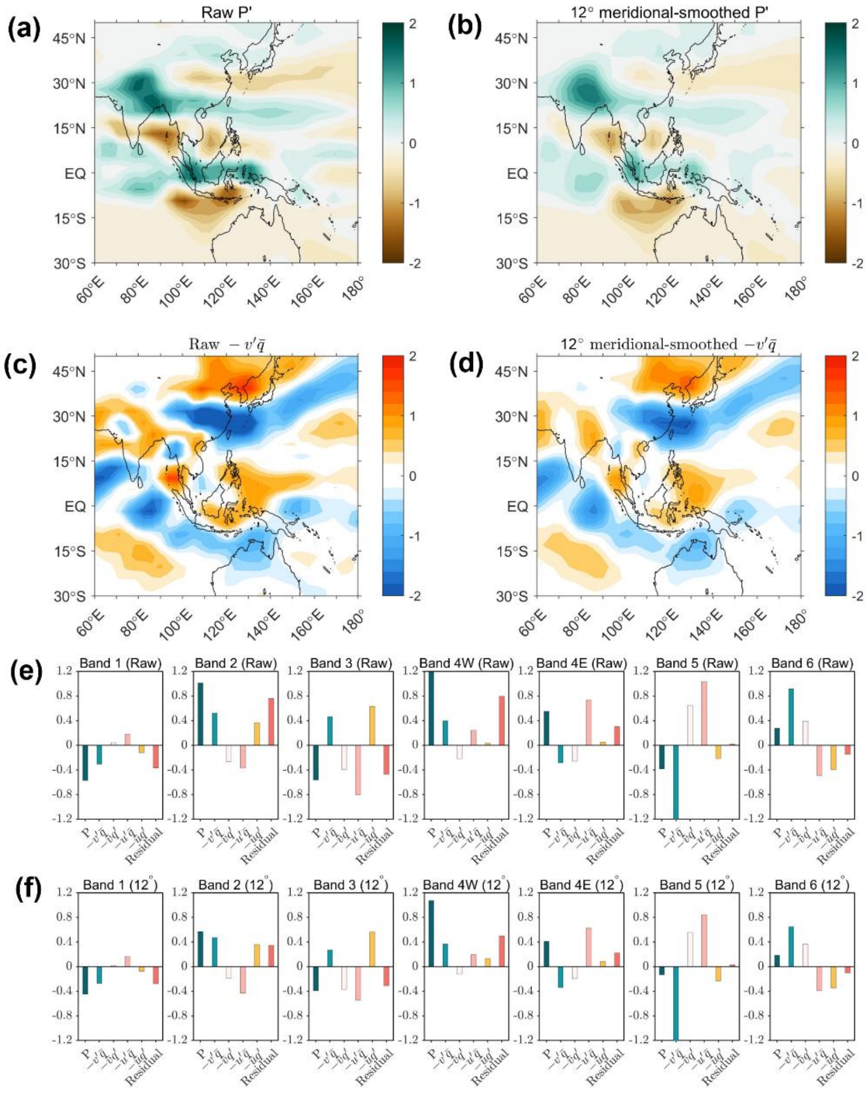

Figure S14. Robustness of banded precipitation structure and meridional moisture advection ($-v'\overline{q}$) under meridional smoothing. Spatial patterns of regressed (top) precipitation anomalies and $-v'\overline{q}$ associated with Rossby wave propagation for (left) the original fields and (right) fields after applying a 12° meridional smoothing. (e) The same as Figure 5c for the original (raw) fields and (f) fields after applying a 12° meridional smoothing.

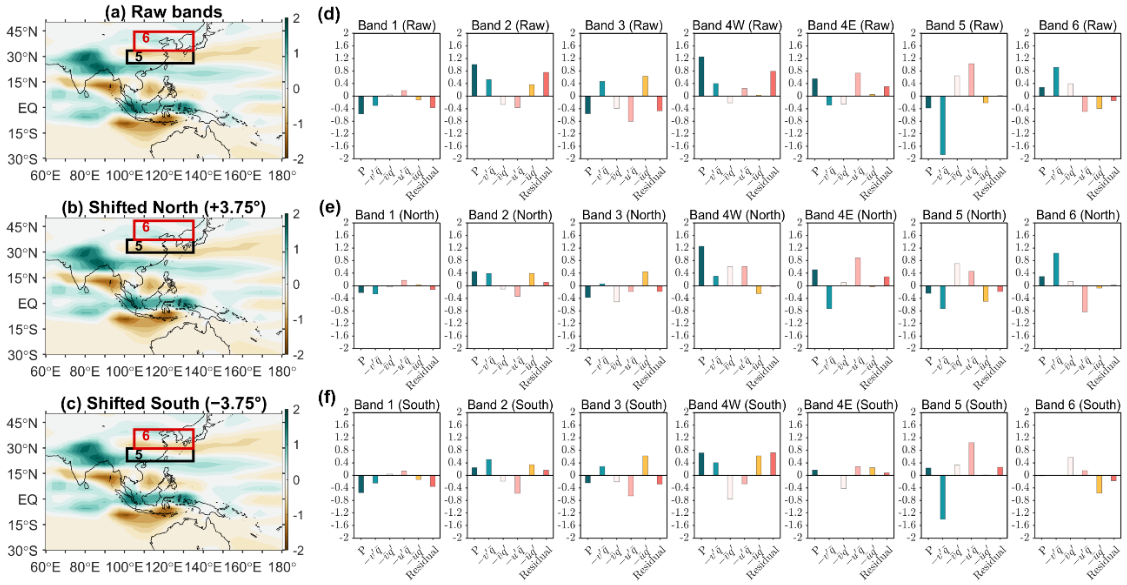

Figure S15. Sensitivity of moisture budget decomposition to meridional shifts of band boundaries. Regressed precipitation anomalies with diagnostic boxes for the (a) original bands, and after shifting all bands northward (+3.75°; b) and southward (−3.75°; c), corresponding to one grid spacing. Labels denote Bands 5 and 6, whose area-mean are sensitive to (c and e) southward shifts because of incorporating opposite-signed anomalies from adjacent regions. (d–f) Area-mean precipitation (P) and individual moisture budget terms for each band under the original, north-shifted, and south-shifted configurations, respectively.

5. The authors correctly clarify that they do not interpret sub-kyr variability, phase analysis is restricted to precession scale, and consistency across records supports robustness. This is largely satisfactory. However, the phase-wheel presentation (Figure 2) still visually invites over-interpretation. The text occasionally implies tighter phase coherence than the data strictly allow. A short statement explicitly cautioning against over-interpreting phase leads/lags would further strengthen credibility.

Response: We appreciate the reviewer's positive and careful feedback. We agree that the phase-wheel diagram (Figure 2) could risk over-interpretation, and that some phrasing may imply stronger phase coherence than supported by the data. **We have added a brief, explicit statement in the text to caution against over-interpreting precise phase leads/lags, emphasizing that our interpretation focuses on precession-band coherence rather than high-precision timing.** These changes could further improve the clarity and robustness of our presentation.

Please see lines 140-150: "*Although individual proxy indicators inherently exhibit distinct limitations, the coherent, latitudinally precession phase progression evident across 18 independent precipitation records (including marine sediments, speleothems, loess, and pollen sequences), collectively reinforces the credibility of our findings. We*

note that the phase-wheel diagram (Figure 2) is intended to illustrate the broad pattern of precession-band phase relationships across records, rather than to support sub-kyr and/or precise interpretation of individual phase leads or lags. The general latitudinal trend in the composite appears consistent across multiple records, but the absolute timing of each record is subject to chronological uncertainties and site-specific biases (Materials and Methods). Readers are cautioned against over-interpreting small differences in phase angles."

6. The revised manuscript still occasionally suggests that the mechanism provides a "unified framework" for orbital hydroclimate variability. This remains too strong, given the reliance on a single model framework, strong boundary-condition assumptions, and lack of ensemble or inter-model confirmation. This should be clearly framed as a proposed mechanism, not a definitive explanation.

Response: We sincerely appreciate the reviewer's careful and constructive comments. We fully agree that the phrase "unified framework" is too strong given the reliance on a single model, boundary condition assumptions, and the absence of ensemble or inter-model comparisons.

We have revised the manuscript to remove such overstated statements and clearly frame our mechanism as **a proposed dynamical interpretation** rather than a definitive or unified explanation. **Corresponding revisions have been made in the Abstract, Results, and Implications sections to ensure a more cautious and accurate presentation.**

These changes clarify the scope of our conclusions and align the interpretation with the level of evidence provided in this study.

Please see Lines 386-395: "*Based on the aforementioned results, we propose that the precession-controlled hexapole latitudinal banding of summer precipitation anomaly could be interpreted as an atmospheric circulation adjustment associated with meridionally propagating Rossby waves (Figure 6). Anomalous convective activity in the IPWP is inferred to act as a Rossby wave source, generating two poleward-propagating wave trains along the South Asian and western Pacific corridors, guided by the summer monsoon circulation. By their influence on large-scale circulation and moisture transport, these Rossby waves appear to modulate the redistribution of tropical moisture and contribute to organize latitude-dependent precipitation bands (Figure 5).*"

Lines 491-502: "*Notably, the role of Rossby waves in shaping precipitation anomaly appears to be organizing, rather than competing with, and thus complementary to other climate factors considered in our study. The hexapole banded structure in both proxy records and model simulations is consistent with the large-scale adjustment and spatial organization of precipitation anomalies associated with Rossby wave dynamics. By modulating atmospheric circulation and moisture transport pathways, Rossby wave provides a dynamically consistent mechanism linking regionally distinct precipitation responses into a coherent latitudinal banding pattern. The absence of Rossby wave-*

mediated moisture transport is associated with substantially reduced mid-latitude precipitation and a greater confinement of precipitation anomalies to low-to-subtropical regions."

Lines 512-536: "*Both the modern PJ tripole pattern and the orbital-scale hexapole system appear to be closely linked to anomalous tropical insolation forcing. This forcing is inferred to modulate the Rossby wave source function and its meridional propagation, establishing teleconnections that shape precipitation anomalies in mid- and high-latitudes. On orbital timescales, the precipitation banding anomalies over the Asia-Pacific exhibit spatial characteristics largely consistent with those of both the pre-industrial and the modern PJ pattern (Figures 1 and S7). This consistency suggests that the modern PJ pattern is a spatially truncated manifestation of a broader, planetary-scale precipitation organization operating at orbital timescales under the modern climate state. When Rossby waves excited by tropical thermal forcing propagate meridionally, their pathways are further modulated by the summer monsoon circulation and other background atmospheric flows, contributing to distinct precipitation characteristics through interactions with other climatic factors (Figure 5). This integrated mechanism offers several key advances. First, it provides a dynamical perspective on the persistence of the PJ pattern across interannual to decadal timescales, consistent with its observed modal stability. Second, it offers a physically consistent interpretation for spatial heterogeneities in paleo-precipitation reconstructions across the Asia-Pacific, including the tripole pattern over eastern China during the last deglaciation (Zhang et al., 2018). Third and critically, orbital-scale precipitation banding anomalies may serve as a physically grounded reference for natural PJ-type variability, which is essential for assessing the contribution of anthropogenic warming to the intensity and frequency of recent and future PJ-related extreme events.*"

If these remaining issues are clearly and transparently addressed, I would be comfortable recommending acceptance after minor-to-moderate revision.

Response: We sincerely appreciate your positive evaluation. We have carefully and transparently addressed all remaining issues to further improve the clarity and rigor of our work. We hope that these revisions will satisfy you. We will continue to actively listen to any further comments and suggestions you may have. Thank you very much for your time and effort.

Reviewer #4:

Dear Editor, dear Authors,

Upon reading the manuscript, I identified flaws in the analysis, interpretation, and conclusions drawn from the data. As shown below, they concern the interpretation of the latitudinal spatial trend in phase relationships between the paleo-climate records and the astronomical solution for precession/insolation shown in Figure 2. These flaws preclude publication.

1) Rossby waves owe their existence to the stratification of the fluid and the variation with the latitude of the Coriolis frequency $f = 2\Omega\sin(\varphi)$ (Ω is the rotation rate of the Earth and φ is the latitude). Since f=0 along the equator (this should be considered a singularity in the equations of movement of Rossby waves), imagining a Rossby wave crossing the equator makes no physical sense (Figure 6). The equator can only trap Rossby and Kelvin waves.

Response: We sincerely appreciate your reading and comments on our manuscript. Your professional insights into the dynamics of Rossby waves and orbital forcing are highly valuable and have prompted us to clarify our physical framework more explicitly. We fully respect your expertise and share your emphasis on rigorous dynamical consistency.

We would like to gently clarify that our interpretation does **not** violate fundamental Rossby wave dynamics, and the apparent divergence arises from a slight mismatch in conceptual understanding. We fully agree that the Coriolis parameter ***f*** vanishes at the equator and that classical extratropical Rossby waves cannot propagate across it. This fundamental dynamical constraint is fully respected in our analysis.

We **do not propose or imply any cross-equatorial propagation of Rossby waves**. In our interpretation, tropical convective heating over the Indo-Pacific Warm Pool acts as a forcing that separately excites Rossby-wave responses in each hemisphere, with Rossby-wave propagation confined within each hemisphere along their respective waveguides. Therefore, the meridionally organized circulation anomalies shown in **Figure 6 represent only the Northern Hemisphere** and should not be interpreted as a single Rossby wave crossing the equator.

This framework follows well established atmospheric dynamics.

- Convective heating over the IPWP excites Matsuno-Gill type Rossby waves and induces circulation anomalies that extend into the subtropics and mid-latitudes (Matsuno 1966; Gill 1980).
- Localized convective heating generates upper-tropospheric divergence, which acts as a Rossby wave source and drives poleward propagating anomalies along the background circulation (Sardeshmukh and Hoskins 1988).
- Such convectively forced Rossby waves organize meridional teleconnection patterns similar to the modern Pacific–Japan (PJ) pattern arises (Nitta 1987; Kosaka and Nakamura 2010; Hu et al.2024).

- Rossby wave Source (RWS) and activity flux (WAF) diagnostics confirm the poleward propagation along the circulation waveguides, organizing coherent precipitation structures (Takaya and Nakamura 2001), consistent with our results (Figures 3-5).

Importantly, our interpretation further grounded in three foundational dynamical principles:

(i) Rossby waves propagate on synoptic to seasonal timescales and redistribute energy (Hoskins and Karoly, 1981),

(ii) the climatological stationary wave pattern represents the time-mean outcome of recurrent transient eddy activity (Lorenz, 1967; Hoskins et al., 1983).

(iii) In paleoclimate studies, orbital forcing is known to modulate stationary wave background configurations, which in turn shape large-scale precipitation patterns (PMIP; e.g., Braconnot et al., 2007; Otto-Bliesner et al., 2017).

Building on these foundations, **our study goes one step further** by explicitly linking seasonal to orbital-timescale atmospheric variability. The simulated precipitation anomalies reflect the cumulative effect of seasonally recurring Rossby wave activity modulated by slow orbital forcing through changing background circulation. This perspective provides a dynamical bridge between fast atmospheric variability and orbital-timescale hydroclimate change, which has not been explicitly articulated in previous studies.

To avoid potential misunderstanding, we have revised the manuscript to explicitly clarify this point, emphasizing that the Figure 6 only represent the Northern Hemispheric responses, not dynamical cross-equatorial propagation of Rossby waves. We have also highlighted this information in the main text and figure cations.

Please see lines 26-48: "*Orbital-scale dynamics of Indo-Pacific Warm Pool (IPWP), Earth's dominant heat and moisture source, exert far-reaching yet incompletely understood influences on global hydroclimate. Here, by synthesizing sedimentary proxies with transient simulations, we identify a coherent, banded precipitation anomaly across the Asia-Pacific region that cannot be explained by regional dynamics alone. We show that seasonal deep convection over the IPWP excites planetary Rossby waves. On orbital timescales, their integrated effects are consistent with precessional forcing, while precession simultaneously modulates the large-scale atmospheric background state that influences their propagation. Guided by summer circulation, these waves propagate poleward and reorganize large-scale moisture transport and precipitation across the mid- and high-latitudes. The cumulative wave response and background-state modulation provide a robust dynamical linkage between tropical convection and extratropical hydroclimate on orbital timescales. This mechanism offers a physically consistent interpretation for how low-latitude orbital forcing imprints hydroclimate variability across Pan-Asia via planetary-wave dynamics.*"

Please see lines 259-262: "*Rossby wave activity is locally excited by anomalous deep*

convection over the IPWP, and propagates strictly poleward within each hemisphere separately, with no cross-equatorial propagation involved."

Please see lines 426-429: "*Rossby wave activity is locally excited by deep convection over the IPWP and propagates poleward independently within each hemisphere, with no cross-equatorial wave propagation is invoked or implied. Only Northern Hemisphere results are presented here.*"

References:

Hoskins, B. J., and D. J. Karoly (1981) The steady linear response of a spherical atmosphere to thermal and orographic forcing. Journal of the Atmospheric Sciences, 38, 1179–1196

Lorenz, E. N. (1967) The Nature and Theory of the General Circulation of the Atmosphere. World Meteorological Organization, Geneva.

Hoskins, B. J., I. N. James, and G. H. White (1983) The shape, propagation and mean-flow interaction of large-scale weather systems. Journal of the Atmospheric Sciences, 40, 1595–1612.

Otto-Bliesner et al. (2017) Climate variability and change since the Last Glacial Maximum: an overview of PMIP4 simulations. Climate of the Past.

Braconnot et al. (2007) Results of PMIP2 coupled simulations of the Mid-Holocene and Last Glacial Maximum. Climate of the Past, 3, 261–277.

Kosaka, Y. & Nakamura, H (2010) Mechanisms of meridional teleconnection observed between a summer monsoon system and a subtropical anticyclone. Part I: The Pacific–Japan pattern. Journal of Climate 23, 5085–5108.

Pinault J-L (2025) The Milankovitch Theory Revisited to Explain the Mid-Pleistocene and Early Quaternary Transitions, Atmosphere, 16, 702.

Nitta, T (1987) Convective Activities in the Tropical Western Pacific and Their Impact on the Northern Hemisphere Summer Circulation. Journal of the Meteorological Society of Japan. Ser. II 65, 373–390

Matsuno, T (1966) Quasi-Geostrophic Motions in the Equatorial Area. Journal of the Meteorological Society of Japan. Ser. II 44, 25–43.

Gill, A. E (1980) Some simple solutions for heat-induced tropical circulation. Quarterly Journal of the Royal Meteorological Society 106, 447–462.

Sardeshmukh, P. D. & Hoskins, B. J (1988) The generation of global rotational flow by steady idealized tropical divergence. Journal of Atmospheric Sciences 45, 1228–1251.

Hu, P. et al. (2024) Revisiting the Linkage Between the Pacific–Japan Pattern and Indian Summer Monsoon Rainfall: The Crucial Role of the Maritime Continent. Geophysical Research Letters 51, e2023GL106982

Takaya, K. & Nakamura, H (2001) A Formulation of a Phase-Independent Wave-Activity Flux for Stationary and Migratory Quasigeostrophic Eddies on a Zonally Varying Basic Flow. Journal of the Atmospheric Sciences 58, 608–627.

2) The orbital forcing of the climate system can only be exerted with Rossby waves of very long apparent wavelength to be in line with their period. On the other hand, the variations in solar irradiance resulting from orbital cycles are so small that a positive feedback loop is necessarily involved in the amplification process. This is why the concept of gyral Rossby waves was introduced, as these waves act as a mediator between variations in solar irradiance and the climate response (the resonance occurs at 24,576 yrs).

Response: We agree with you that orbital-scale variability requires long-wavelength, low-frequency dynamics and that insolation changes alone are too weak to drive climate signals without positive feedback.

However, our interpretation does **not** require "orbital Rossby waves". Instead (also see Response to Comment 1):

- Rossby waves themselves remain **fast, synoptic–seasonal phenomena**;
- Orbital forcing (precession) slowly **modulates the mean background** state, including IPWP tropical convection, upper-ocean thermal conditions, and the strength and position of the summer monsoon circulation and associated waveguide;
- These fast wave responses are **repeatedly and seasonally excited by tropical heating and time-integrated over orbital cycles**, leaving a persistent, large-scale precipitation pattern.

Therefore, this response is **not produced by a single long-period wave, but by the cumulative effect of many fast wave events** whose amplitude, frequency, and spatial structure are systematically modulated by orbital forcing. This concept—**fast dynamics modulated by slow forcing**—is widely used in paleoclimate dynamics and does not require the existence of "orbital-period Rossby waves."

In this mechanism:

(i) Orbital precession does not act directly on fast wave dynamics. Instead, it modulates the interhemispheric insolation gradient (r = -0.90; Figure 2) and the associated deep convection and upper-ocean heat content over the IPWP.

(ii) This slowly varying thermal background regulates the **Rossby-wave response of the summer mean state**, including its source strength and meridional propagation characteristics.

(iii) Through repeated seasonal stationary-wave adjustment and waveguiding, these circulation anomalies organize meridional moisture redistribution. When integrated over orbital timescales, this process yields a persistent, latitudinally banded precipitation pattern, rather than a snapshot of transient synoptic events.

We support this interpretation with four independent diagnostics:

(1) Rossby Wave Source (RWS) and Wave Activity Flux (WAF) analyses identify the warm pool as the dominant wave source and coherent meridional propagation (Figures

3-4).

(2) Moisture budget diagnostics further show that wave-induced meridional moisture advection dominates extratropical precipitation anomalies (Figure 5).

(3) Seasonal sensitivity analyses confirm that the mechanism operates only under the summer mean-state waveguide (Figures S17-18).

(4) Warm-pool forcing suppression experiment (Hu et al., 2024 and strong-weak Rossby contrast (Figure S13) verify the essential role of Rossby waves driven by tropical thermal forcing to the banded precipitation pattern.

We greatly respect the pioneering work on gyral Rossby waves as a mediator of orbital forcing. This concept addresses long-period oceanic Rossby waves and is highly important for understanding slow climate feedbacks. However, our study focuses on **atmospheric Rossby waves** excited by tropical convection, guided by summer monsoon flow at each hemisphere. This mechanism does not require resonance at orbital frequencies, but instead relies on modulation of the statistical properties of fast atmospheric variability. **These two mechanisms describe different components of the climate system (atmospheric vs. oceanic; fast-response wave propagation vs. long-period oceanic gyre waves) and are complementary, not contradictory.** Both illustrate the importance of wave dynamics in translating orbital forcing into hydroclimate variability.

We note that the reference suggested by the reviewer elegantly introduces the concept of gyral Rossby waves, which we agree is likely highly relevant for explaining the Mid-Pleistocene Transition (MPT) and the Early Quaternary Transition (EQT) (Pinault et al., 2022; 2025). However, this innovative line of work lies beyond the scope of our study, which focuses on atmospheric Rossby waves. Future studies are needed to explore these topics in greater detail.

We made a series of revisions in the main text to avoid mismatch in conceptual understanding and to explicitly state the role of timescale separation and cumulative effects in linking orbital forcing to atmospheric Rossby wave–mediated hydroclimate variability.

We added additional content to make this argument clear.
Please see lines 396-399: *"In contrast to invoking long-period Rossby waves operating directly at orbital timescales (Pinault, 2025), we focus on fast, seasonal-scale atmospheric Rossby waves that are repeatedly excited by tropical convection and accumulate over orbital timescales through stable spatial superposition, as supported by both paleoclimate reconstructions and transient simulations in this study.*"
Please see lines 413-418: *"This interpretation differs fundamentally from resonant oceanic gyral Rossby waves that mediate orbital signals via long-period feedbacks (Pinault, 20222; 2025). Atmospheric and oceanic wave processes act on distinct timescales within different components of the climate system, rendering them complementary rather than contradictory. Together, they highlight the diverse roles of*

wave dynamics in translating orbital forcing into regional hydroclimate variability."

References:

Pinault J-L (2022) A Review of the Role of the Oceanic Rossby Waves in Climate Variability, J. Mar. Sci. Eng. 10, 493.

Pinault J-L (2025) The Milankovitch Theory Revisited to Explain the Mid-Pleistocene and Early Quaternary Transitions, Atmosphere, 16, 702.

Hu, P. et al. (2024) Revisiting the Linkage Between the Pacific–Japan Pattern and Indian Summer Monsoon Rainfall: The Crucial Role of the Maritime Continent. Geophysical Research Letters 51, e2023GL106982

Therefore, the phase-shifts observed in Figure 2 probably result either from diagenesis processes in the case of sediments, or from maturation processes within the epikarst in the case of speleothems. Under no circumstances can they be justified based on exogenous phenomena.

Pinault J-L, A Review of the Role of the Oceanic Rossby Waves in Climate Variability, J. Mar. Sci. Eng. 2022, 10, 493.

Pinault J-L, The Milankovitch Theory Revisited to Explain the Mid-Pleistocene and Early Quaternary Transitions, Atmosphere 2025, 16, 702.

Response: Thank you for point out that diagenesis (sediments) or epikarst maturation (speleothems) could impacted the phase shifts among records. We acknowledge that such local processes can influence individual records.

However, the coherent, latitudinally systematic phase progression across 18 independent precipitation records (marine sediments, speleothems, loess, pollen) and fully coupled climate simulations are difficult to reconcile with diagenetic or epigenetic processes. The pattern matches the dynamical structure of meridionally propagating Rossby wave trains, with phase delays consistent with wave propagation speed and distance from the heat source (Materials and Methods, Figures S1-4 and Table S1).

We have added sentences in the revised manuscript to illustrate this point.

Please see lines 137-147: "*Although individual proxy indicators inherently exhibit distinct limitations, the coherent, latitudinally precession phase progression evident across 18 independent precipitation records (including marine sediments, speleothems, loess, and pollen sequences), collectively reinforces the credibility of our findings. We note that the phase-wheel diagram (Figure 2) is intended to illustrate the broad pattern of precession-band phase relationships across records, rather than to support sub-kyr and/or precise interpretation of individual phase leads or lags. The general latitudinal trend in the composite appears consistent across multiple records, but the absolute timing of each record is subject to chronological uncertainties and site-specific biases (Materials and Methods). Readers are cautioned against over-interpreting small differences in phase angles*."

Response to Comments

Reviewer #3:

The manuscript has been substantially improved following revision, and I appreciate the authors' serious efforts in addressing the previous concerns. In particular, the clarification that Rossby waves are treated as seasonally recurrent atmospheric responses rather than orbital-timescale waves themselves significantly improves the physical consistency of the proposed mechanism. The addition of Rossby wave source (RWS), wave activity flux (WAF), moisture budget diagnostics, and the strong–weak Rossby-wave composite analyses provides much stronger dynamical support for the interpretation. The expanded discussion of alternative mechanisms (ITCZ, Hadley circulation, WPSH, monsoon circulation), together with the new sensitivity analyses on band definitions, also strengthens the robustness of the conclusions.

I also appreciate the authors' efforts to improve the Methods section, especially regarding the transient simulation setup, forcing implementation, and validation against modern circulation and moisture transport fields. The newly added validation figures and discussion help demonstrate that the model reasonably captures the large-scale circulation structures relevant to the proposed mechanism.

Overall, I find that my major scientific concerns have now been sufficiently addressed, and the manuscript is now much clearer, more balanced, and physically better justified. While some limitations remain inherent to the experimental design (e.g., fixed land–sea mask, absence of freshwater forcing, and lack of dedicated sensitivity experiments), the authors now discuss these limitations appropriately and avoid overextending the interpretation.

I therefore support publication after minor revision. Before final acceptance, I recommend the authors carefully clean the manuscript text, as the current version still contains a number of editing artifacts, duplicated wording, figure-number inconsistencies, and grammatical issues that appear to result from track-change merging. These are primarily editorial issues and do not affect the scientific merit of the study.

Response: We thank the reviewer for the positive assessment and constructive suggestions. We have carefully cleaned the manuscript and corrected editing artifacts, duplicated wording, figure-number inconsistencies, and grammatical issues introduced during track-change merging. These revisions were editorial in nature and did not alter the scientific questions, results, or conclusions of the study.

Reviewer #4:

Dear Editor, dear Authors,

Many thanks to the authors for their work clarifying the extratropical response of the upper atmosphere to equatorial Rossby waves resulting from deep convection processes. In particular, thank you for emphasizing the overlap of two timescales: one seasonal, the other of orbital origin.

However, ambiguity remains regarding the formation of extratropical Rossby waves moving poleward. This is not possible within the framework of the generally accepted theory of Rossby waves, which assumes that they are sensitive to latitudinal variations in the Coriolis parameter. But I cannot blame the authors because this is not always clear in the literature, unless redefining the concept of Rossby wave. I rather suggest the terminology " Extratropical divergence-convergence structures".

So, this is a minor remark, and I leave the authors to their own judgment. The response of the upper atmosphere in the form of concentric waves around transient equatorial Rossby waves has been documented by several authors and the corresponding articles are well referenced in the manuscript.

I believe the manuscript meets the journal's criteria. In particular, it is innovative and of major interest to the field and related fields. The methodology is rigorous and clearly explained.

Response: We sincerely thank the reviewer for the thoughtful and constructive comments regarding the interpretation and terminology of Rossby-wave dynamics. We agree that the original wording could lead to ambiguity under the classical framework of freely propagating Rossby waves.

Following the reviewer's suggestion, we have carefully revised the manuscript to clarify that our interpretation refers to Rossby wave-related atmospheric circulation responses, wave activity dispersion, and large-scale teleconnection adjustment, rather than freely propagating orbital-timescale Rossby waves themselves. Specifically, **we have replaced expressions** like "meridionally propagating Rossby waves" **with more precise terminology**: *Rossby wave activity, Rossby wave-mediated circulation response, poleward extension of Rossby wave activity, and large-scale meridional teleconnections.* No scientific conclusions were changed.

We appreciate the reviewer's suggestion, which has helped us substantially improve the precision and clarity of the dynamical interpretation throughout the manuscript.

Rossby wave-regulated orbital precipitation in the Asia-Pacific region

Z. Yu et al. (submitted for review to *Nature Communications*)

This paper presents a new dataset from the last ~150 kyr that adds to a growing body of Pleistocene paleoclimate information for the Indo-pacific warm pool and adjacent continental areas, particularly Asia. The authors also conduct climate modeling to contextualize anomalous observations that are thought to be not well explained by traditional forcing mechanisms of Indo-Pacific climate. My expertise on modeling is limited and therefore cannot comment on that aspect of the study.

The study attempts to understand why there are leads and lags in paleoclimate records from equatorial, to tropical, and finally subtropical Asia. The leads and lags are defined from correlations with the intensity of summer insolation modulated by the orbital cycle of climatic precession (20 kyr). An ultimate goal of the research is to further elucidate the underlying controls on the Asian monsoon so that we are better equipped to study changes in the monsoon that may arise from anthropogenic impacts.

The authors offer a novel and innovative hypothesis that the (1) offsets between the astronomical solutions, and the (2) spatial differences between the paleo-monsoon records are a function of Rossby waves that facilitate transport of moisture outward from the Indo-Pacific warm pool. Rossby waves deliver different rainfall amounts according to the latitude along a poleward (meridional) transect.

Minor concerns

- Line 43: remove the article “the” from “… the hydrological circulation…”

- The acronym OHC is not defined in the paper. [ocean heat conditions?]

- Fig.1b: is “More rainfall” with the arrow just for record (3) or does this apply for all six records? Which direction on the Y axis for the other five records also indicates more rainfall?

Slapdash concerns

Figure 4: incomplete/not labeled correctly according to the figure caption; no blue arrows or red arrows for example.

Major concerns

1. To what extent do all the paleoclimate sediment cores have the same level of chronostratigraphy?
    - Are the offsets due to the empirical error associated with chronology of each record?
    - Six different records with six different climate proxy methods
        - Why should they all behave the same?
        - How about repeating the experiment using the same proxy method for all six rainfall bands? Are the same results resolved?

The problem with the authors interpretation of smectite is that it is not simply warm, humid tropical climate equals greater smectite concentrations, as they posit. In tropical coastal environments that weather basalt rock (Eggleton et al., 1987) smectite in an early stage/less intense weathering product. The main weathering product is mixtures of kaolinite and Ferric oxyhydroxides, which increase under warm, humid climates. Smectite formation from basalt is favored by wet-dry climates (i.e., monsoons) that have a pronounced dry season, i.e., a net deficit of effective moisture (Kantor and Schwertmann, 1974; Deepthy and Balakrishnan, 2005; Zaarur et al., 2024). This is why, to me, it is not unexpected that the author’s smectite/(illite-chlorite) ratio gets bigger under higher precession. Maxima in precession are associated

with minima in tropical insolation and thus dry monsoons. Therefore, elevated smectite is correlating with dryness as one would expect and is not anomalous as the author's would lead us to believe.

2. The "Remarkable" phase shift phenomenon is **an overstatement of the data**
    - So-called trends in phase angles are defined by an outlier in each case
        - The speleothem and 10Be phase angles are effectively the same; the Eu/Eu* record is practically in-phase with precession; I don't see a meridional trend, just the Eu/Eu* record is an outlier as compared to the other two record.
        - Similarly, the pollen record is an outlier as compared to the new clay record and the XRF record.
        - There are no trends just outliers

3. The six rainfall bands **are not uniquely correlated to Rossby waves**.
    - The main areas of intensified rainfall are the south Asian landmass and the Maritime Continent.
    - Weaker rainfall areas are present over the surrounding ocean waters
    - The most parsimonious explanation for the data is that the authors have reproduced the rainfall patterns for the two largest precipitation "hotspots" of Asia. This is clearly an artifact of the seasonality of the monsoon.
    - It would appear that of the hypotheses, the authors model data supports the hypothesis that the spatial differences in precipitation are due to land-sea thermal contrasts as proposed by Cheng et al. (Cheng et al., 2022). So I am not understanding why this simpler explanation of monsoonal dynamics is supplanted by the more complex argument of Rossby waves?

References

Cheng, H., Li, H., Sha, L., Sinha, A., Shi, Z., Yin, Q., Lu, Z., Zhao, D., Cai, Y., Hu, Y., Hao, Q., Tian, J., Kathayat, G., Dong, X., Zhao, J., Zhang, H., 2022. Milankovitch theory and monsoon. The Innovation 3, 100338.

Deepthy, R., Balakrishnan, S., 2005. Climatic control on clay mineral formation: Evidence from weathering profiles developed on either side of the Western Ghats. J Earth Syst Sci 114, 545–556.

Eggleton, R. A., Foudoulis, C., Varkevisser, D., 1987. Weathering of Basalt: Changes in Rock Chemistry and Mineralogy. Clays and clay miner. 35, 161–169.

Kantor, W., Schwertmann, U., 1974. MINERALOGY AND GENESIS OF CLAYS IN RED-BLACK SOIL TOPOSEQUENCES ON BASIC IGNEOUS ROCKS IN KENYA. Journal of Soil Science 25, 67–78.

Zaarur, S., Matmon, A., Rotshtein, R., 2024. Old basalts, young soils - Age constraints for the Golan Heights plateau volcanic soils. Earth Surf Processes Landf 49, 1796–1806.
